# Dual mobility versus standard cups in total hip replacement for displaced femoral neck fractures (Duality): an international, multicentre, randomised, controlled, superiority trial

**DOI:** 10.1016/S0140-6736(26)00759-2

**Published:** 2026-07-25

**Authors:** Nils P Hailer, Xavier L Griffin, Sebastian Mukka, Tatevik Ghukasyan Lakic, Duncan Appelbe, Olof Sköldenberg, Sarah E Lamb, Ollie Östlund, Olof Wolf, DUALITY Investigators, Krister Arlinger, James Berstock, Rasmus Bjerre, Frida Boström, Christopher Buckle, Hans-Peter Bögl, Hanne Carlsen, Ann-Charlotte Claesson, Owen Diamond, Doug Dunlop, Gunnar Flivik, Kieran Gallagher, Matt Gee, Sam Heaton, Christian Hellerfelt, Anders Isacsson, Saif Ul Islam, Nicole Jessen, Theophilus Joachim, Per-Erik Johanson, Kamal Kadum, Benjamin Kapur, Andrew Kelly, Lotta Kettil, Johan Kärrholm, Paul Magill, Maria Mannberg, Andrew McAndrew, Rory Middleton, Maziar Mohaddes, Sebastian Mukka, Michael Möller, Elin Nemlander, Aaron Ng, René Notelid, Maja Notini, Linnea Nyström, Konstantinos Papadopoulos, William Poole, Mike Reed, Ian Dos Remedios, Gareth Roberts, Cecilia Rogmark, Ola Rolfson, Marshall Sangster, Jörg Schilcher, Monica Sjöholm, Ayman Sorial, Daniel Stam, Jonas Sundkvist, Gustav Trehn, Konstantinos Tsitskaris, Girish Vashista, Nils von Wachenfelt, Rathan Yarlagadda, Vasileios Zampelis

**Affiliations:** a Department of Surgical Sciences/Orthopaedics & Hand Surgery, Uppsala University, Uppsala, Sweden; b Bone and Joint Health, Blizard Institute, Queen Mary University of London, London, UK; c Department of Diagnostics and Intervention (Orthopaedics), Umeå University, Umeå; d Uppsala University, Uppsala Clinical Research Center, Uppsala, Sweden; e Nuffield Department of Orthopaedics, Rheumatology and Musculoskeletal Sciences, University of Oxford, Oxford, UK; f Karolinska Institute, Department of Clinical Sciences at Danderyd Hospital, Stockholm, Sweden; g Department of Public Health and Sports Sciences, Exeter Medical School, St Luke's Campus, University of Exeter, Exeter, UK

## Abstract

**Background:**

Dislocation is the most common early surgical complication in patients with hip fractures treated with total hip replacement (THR). Dual mobility total hip replacement (DM-THR) was developed to increase joint stability, but no randomised trial has investigated its efficacy and safety. We aimed to compare the risk of dislocation in patients receiving DM-THR versus conventional THR.

**Methods:**

We included people aged 65 years or older with a displaced femoral neck fracture attending 20 Swedish and 24 UK hospitals in this pragmatic, registry-based, randomised superiority trial. Participants eligible for THR were randomly allocated in a 1:1 ratio to either DM-THR or standard THR with the use of remote, web-based, country-specific sequences. Neither participants nor members of the direct clinical care teams were masked, but research investigators and statisticians were masked until the trial analysis was completed. The primary outcome was dislocation of the index joint, treated with closed reduction or open surgery within 1 year, analysed in the modified intention-to-treat population including all consenting participants receiving any type of hip replacement and excluding those with major protocol deviations. This trial is registered with ClinicalTrials.gov (NCT03909815) and ISRCTN11895196.

**Findings:**

Between Jan 9, 2020, and April 2, 2024, 2934 eligible patients were screened, of whom 1334 were not enrolled, mainly due to surgeon's preference (n=560), because they had already been treated at the time of screening (n=257), or because informed consent was not obtained (n=213). 1600 participants (64% female, 36% male) were thus randomly assigned to either DM-THR (n=798) or THR (n=802). After exclusion of 34 participants due to prespecified protocol deviations, 1566 participants (97·9%) contributed to the modified intention-to-treat analysis. A dislocation, the primary outcome, occurred in ten (1·3%) of 779 participants in the DM-THR group and in 33 (4·2%) of 787 in the THR group (adjusted hazard ratio 0·27, 95% CI 0·13−0·56; p<0·0001).

**Interpretation:**

The use of DM-THR substantially reduced the risk of dislocation and of any surgical complication among people treated with THR after a displaced femoral neck fracture. DM-THR can thus be recommended for this group of patients.

**Funding:**

The Swedish Research Council and the National Institute for Health and Care Research.

## Introduction

Hip fractures in people older than 60 years are among the most common injuries globally, annually affecting more than 14 million individuals.^1^ The global impact of hip fractures is estimated at 1·75 million disability-adjusted life-years lost and represents 1·4% of the total direct health-care expenditure in established market economies.^2^ Femoral neck fractures in which the bone fragments are displaced represent approximately half of all hip fractures. These fractures are treated with a hip replacement; patients who are independently mobile, have few comorbidities, and are cognitively intact might be suitable for a total hip replacement (THR) in which both the ball and the socket of the hip joint are replaced with metal and plastic implants. In the UK and the Nordic countries, about 20% of patients who undergo hip replacements for a displaced femoral neck fracture receive THR.^3^, ^4^

The standard type of THR involves a single joint between a relatively small ball and the socket, which is associated with a risk of dislocation, when the ball disengages with the socket. This is the most common early surgical complication^5^ and requires closed or open surgical reduction. This complication often leads to revision surgery, impaired quality of life with the anxiety of dislocating again, and imposes a substantial resource burden.^6^, ^7^, ^8^ Dislocation and revision for dislocation are associated with poorer outcomes.^9^

**Research in context**

**Evidence before this study**

Total hip replacement (THR), in which both the ball and the socket of the hip are replaced, is recommended for older, active individuals with a displaced fracture of the femoral neck. The most common surgical complication after THR for hip fracture is dislocation, occurring in 6–10% of patients. Dual mobility (DM)-THR, in which the small ball of the THR is encased in a much larger plastic ball, creating two articulations, was developed to reduce dislocation risk. We performed a search between Dec 17, 2018 and July 10, 2020, and then repeated the search iteratively throughout the trial, of MEDLINE, Embase, Web of Science, the Cochrane Database of Systematic Reviews, Cochrane Central Register of Controlled Trials, Database of Abstracts of Reviews of Effects, Health Technology Assessment database, Epistemonikos, Proquest Dissertations and Theses, and National Technical Information Service, for evidence comparing the risk of dislocation in DM-THR with conventional THR. We used combinations of the terms “(femoral) fracture”, “hip (s)”, “(trans-) cervical”, “intracapsular”, “arthroplasty”, “replacement”, “hip prosthesis”, ”joint prosthesis”, and “randomised controlled trial”, with Boolean operators and truncation as appropriate. Multiple non-randomised studies and two small randomised trials suggest that DM-THR might be associated with a reduced dislocation risk—but increased risk of prosthetic joint infection. A Cochrane review published in 2022 identified few, small randomised trials and encouraged researchers to investigate the efficacy of DM-THR. To date, no pivotal trial has provided definitive evidence of the efficacy and safety of DM-THR compared with standard THR.

**Added value of this study**

The Duality trial randomly allocated 1600 participants aged 65 years or older treated with THR for a displaced femoral neck fracture at 20 hospitals in Sweden and 24 in the UK to either DM-THR or standard THR. 98% of participants provided data for the primary intention-to-treat analysis. The study found a statistically significant and clinically important reduced risk of dislocation for DM-THR after 1 year. The risk of any surgical complication was also reduced in the DM-THR group; there were no differences between groups regarding other secondary outcomes.

**Implications of all the available evidence**

The Duality trial provides evidence that DM-THR reduces the risk of the most common surgical complication for patients with a displaced femoral neck fracture treated with a THR. This information is important for clinicians and policy makers alike, and implementation into clinical practice is both feasible and desirable to improve care of older adults with this common and hazardous injury.

Dual mobility (DM)-THR is an alternative design of replacement intended to reduce the risk of dislocation. With this implant, the hip joint is still replaced, but the small ball of the THR is encased in a much larger plastic ball, creating two articulations. This implant design improves the stability of the replacement by increasing the range of movement of the joint before dislocation. Large non-randomised studies provide conflicting evidence about whether DM-THR is associated with a reduced risk of dislocation.^10^, ^11^, ^12^, ^13^, ^14^ DM-THR is associated with an increased risk of prosthetic joint infection or wear of the plastic THR components,^15^, ^16^ and costs about twice that of a standard THR.

The aim of this randomised trial was to compare the risk of dislocation after DM-THR versus standard articulation THR in the treatment of active patients aged 65 years or older with an acute, displaced femoral neck fracture.

## Methods

### Study design and participants

Duality was an international, pragmatic, registry-based, randomised superiority trial conducted in Sweden and the UK. The trial protocol was developed in Sweden in 2019, and a UK study arm was subsequently planned with the use of the same protocol with minor amendments to facilitate local implementation (appendix 2 pp 1–26, and appendix 3 pp 1–28). In the UK the trial was conducted within the WHiTE Platform,^17^ where additional outcomes are routinely collected that were not required for the international protocol and so are not reported here: modified New Mobility Score and residential status. The trial was conducted in 20 hospitals in Sweden and 24 in the UK. Eligible Swedish patients were identified by the Swedish Fracture Register trial platform based on patient age and fracture classification when they were admitted to local emergency departments.^18^ In the UK, eligible patients were recruited directly from participating hospitals.

The trial was conducted by two national management committees chaired by the national lead investigators; oversight was provided by independent steering committees and data safety monitoring committees in both countries. The trial was conducted in accordance with the Declaration of Helsinki, and the study protocol was approved by the Swedish Ethical Review Authority (reference 2019–01137) and NHS Research Ethics Committee (reference 20/SC/0452). All participants provided informed consent. The study protocol describing the Swedish study arm has been published;^19^ the final clinical study protocol with all relevant amendments, including the addition of the UK study arm, was published on the trial homepage (appendix 1 p 22). A single statistical analysis plan (appendix 4 pp 1–48) was finalised and signed by the trial statistician before analysis and any unmasking.

All patients aged 65 years or older with an acute displaced femoral neck fracture and considered suitable for THR by the admitting physician were screened. Suitability for THR was based on patients being independently mobile, having few medical comorbidities, and being cognitively intact, but, ultimately, the shared decision for a THR rather than an alternative treatment was based on clinical judgement. Patients with cognitive impairment (as assessed by the admitting physician), a delayed diagnosis (>7 days after trauma), pathological or stress fracture, or a fracture adjacent to a previous ipsilateral hip implant, and people previously included in the study, were excluded. Reasons for exclusion of patients after initial screening were summarised along the categories: surgeon's preference, already treated, absence of informed consent, not suitable, lack of clinical resources (usually indicating that both acetabular implants were not available on that specific date at that site, or a lack of resources to conduct clinical trials during the COVID-19 pandemic), and other. The category surgeon's preference was available only in the UK arm of the trial, indicating that surgeons felt no equipoise and chose their preferred implant for this specific patient, without additional reasons given. Patient and public involvement was embedded during the planning of the trial, in Sweden by patient representatives in the steering committee of the Swedish Arthroplasty Register and in the UK through the Bone and Joint Health patient partner panel.

### Randomisation and masking

Participants were randomly assigned to receive either a DM-THR or a standard THR in a 1:1 ratio, stratified by country, with the use of remote, web-based, country-specific sequences, each generated by independent statisticians who were not involved in the statistical analysis. Neither participants nor members of the direct clinical care teams were masked, but the latter were not involved in participant follow-up. Research investigators and statisticians were masked until the trial analysis was completed.

### Procedures

Before the start of the trial, both treatments had been in regular use at each participating site, as verified by each country's registry data. All surgeons operating on trial participants were familiar with the implants they used. The choice of specific implants, fixation of components, surgical approach, perioperative care, and rehabilitation were left to the discretion of each site's clinical team according to local practice. The study protocol required sites monitored to ensure that co-interventions were consistent across treatment groups.

### Outcomes

In Sweden, details of the surgical procedure, complications, and re-operations were collected from the Swedish Arthroplasty Register, the Swedish National Patient Register, and hospital medical chart review. Data collection in the UK was completed by direct participant follow-up, the National Joint Registry of England and Wales, and hospital medical chart review. Excellent coverage and completeness of these data sources have been ascertained in previous studies and in annually performed validations.^4^, ^20^, ^21^

The primary outcome was dislocation of the index replacement, treated with manipulation under anaesthesia or sedation, also termed closed reduction, or with re-operation to perform open reduction of the joint within 1 year. The medical charts of participants were individually reviewed to confirm the outcome event and the laterality of that event when a contralateral THR was present, expected in approximately 20% of participants.

Secondary outcomes were death at 90 days and at 1 year; all-cause re-operation of the index replacement; prosthetic joint infection of the index replacement; health-related quality of life measured with the EQ-5D-5L; a composite of dislocation and mortality; and any surgical complication, a composite of dislocation, prosthetic joint infection, or re-operation, all over 1 year. The composite of dislocation and mortality, prespecified in our statistical analysis plan, was a composite of our main outcome measures of efficacy and safety. A formal health economic analysis is planned as a separate publication.

### Statistical analysis

We assumed an incidence of dislocation with standard THR (control) of 7% within 1 year and a hazard ratio (HR) of 0·5 between treatments.^11^, ^22^, ^23^ Based upon our simulations, assuming a constant risk with random censoring due to death assumed to occur exponentially at 10% per annum, a total sample of 1600 participants would detect the target difference with an α of 0·05 and 88% power. We additionally explored other plausible scenarios: if the control group's 1-year event rate had been 8% rather than 7%, a sample size of 1600 would have yielded 91% power, and, in contrast, we would have retained 80% power to detect a slightly more modest effect size corresponding to an HR up to 0·57.

**Figure 1:**
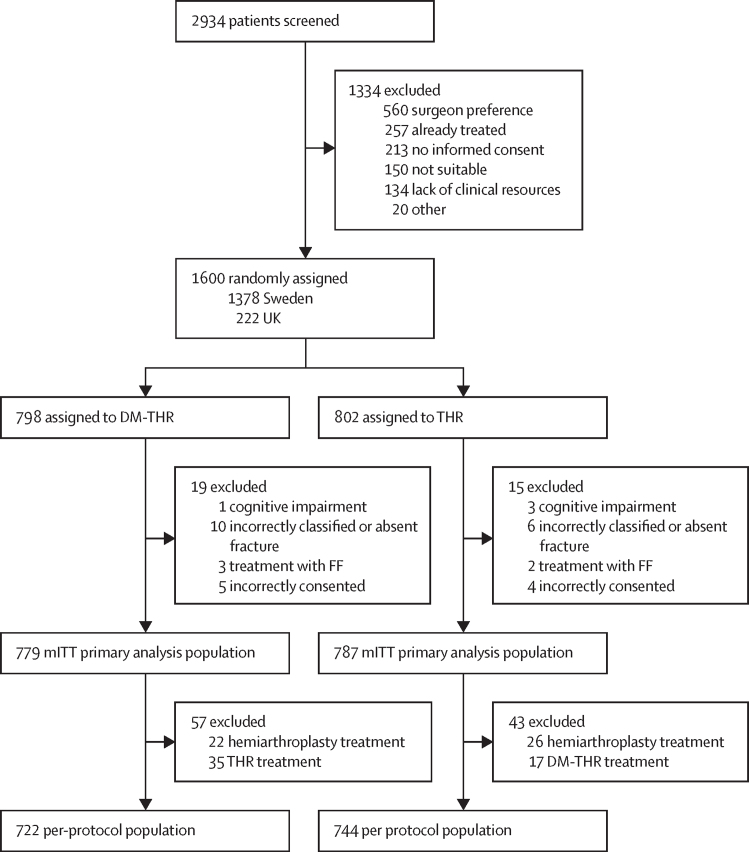
Trial profile DM-THR=dual mobility total hip replacement. FF=fracture fixation. mITT=modified intention-to-treat. THR=total hip replacement.

The modified intention-to-treat (mITT) population included all participants receiving any type of hip replacement, including hemiarthroplasty, a type of hip replacement in which no cup is implanted (table 1, appendix 1 pp 10, 12–13). Participants undergoing fixation of the fracture with plates and screws instead of replacement who could not experience the primary outcome, as well as participants with other major protocol deviations (eg, dementia, incorrectly documented consent, incorrect hip fracture type, or absence of any hip fracture), were excluded from the mITT population. In all analyses using the mITT population, participants were presented in the treatment group to which they were originally randomly assigned. Baseline characteristics, outcomes, and treatment adherence were reported with standard statistical summaries. Kaplan–Meier plots and event rate tables were used to describe all time-to-event outcomes by randomised treatment. Primary efficacy analyses were performed with the use of Cox proportional hazards regression models, adjusted for age (included as a linear covariate on the log-hazard scale), sex, surgical approach (lateral and posterior), and country. Treatment effects were presented as HR with 95% CIs and two-sided p values for the null hypothesis. The proportional hazards assumption was evaluated through visual inspection of Schoenfeld residuals. Sensitivity analyses were performed in the same population including only randomised treatment, and both adjusted and unadjusted models were fitted to the per-protocol population from which participants who had crossed over to the alternative treatment arm and individuals who had received hemiarthroplasties were excluded (figure 1). Supplementary analyses prespecified in the statistical analysis plan included subgroup analyses by age, sex, surgical approach (divided into the two categories lateral and posterior), femoral neck length, country, American Society of Anaesthesiologists score, and BMI, by fitting an interaction term between the subgroup and treatment. Responses from the EQ-5D-5L questionnaires were mapped onto the UK preference-based tariffs, with the use of the Hernandez–Alava mapping model to derive utility.^24^ The utility was analysed with a linear regression model with robust standard errors**,** adjusted for the baseline measurement. All data management and statistical analyses were performed with SAS (version 9.4). The trial was registered with NCT03909815 and ISRCTN11895196.

**Table 1:** Participant demographical characteristics by randomised treatment (modified intention-to-treat population)

		**DM-THR (n=779)**	**THR (n=787)**
Age, years		76·1 (6·0)	76·1 (6·1)
Sex			
	Female	492 (63·2%)	518 (65·8%)
	Male	287 (36·8%)	269 (34·2%)
Country			
	Sweden	669 (85·9%)	676 (85·9%)
	UK	110 (14·1%)	111 (14·1%)
Fracture type			
	Femoral neck fracture	752 (96·5%)	738 (93·8%)
	Basocervical fracture	27 (3·5%)	49 (6·2%)
BMI		24·8 (4·2)	24·7 (4·1)
ASA score			
	1	63 (8·1%)	73 (9·3%)
	2	440 (56·5%)	444 (56·4%)
	3	257 (33·0%)	251 (31·9%)
	4*	9 (1·2%)	11 (1·4%)
	Missing	10 (1·3%)	8 (1·0%)
Surgical approach			
	Direct lateral	369 (47·4%)	402 (51·1%)
	Posterior	409 (52·5%)	383 (48·7%)
	Missing	1 (0·1%)	2 (0·3%)
Type of replacement, n (%)			
	Cemented THR	609 (78·2%)	657 (83·5%)
	Hybrid THR	144 (18·5%)	94 (11·9%)
	Reverse hybrid THR	2 (0·3%)	2 (0·3%)
	Cementless THR	2 (0·3%)	8 (1·0%)
	Cemented hemiarthroplasty	22 (2·8%)	26 (3·3%)
Cup diameter (mm)		50 (38–66)	50 (42–64)
Femoral head diameter† (mm)		28 (22–54)	32 (22–52)
Femoral neck length			
	Standard	590 (75·7%)	597 (75·9%)
	Extended	155 (19·9%)	137 (17·4%)
	Extra extended	6 (0·8%)	15 (1·9%)
	Missing	28 (3·6%)	38 (4·8%)
EQ-5D-5L utility score‡		0·82 (0·20)	0·81 (0·23)
EQ-5D-5L VAS score§		77·3 (19·4)	76·6 (20·4)

Data are mean (SD), number (%), or median (range). Percentages might not total 100 because of rounding. The modified intention-to-treat population included all randomly assigned participants, except 34 who were randomly assigned without properly documented consent or were unable to consent, had other fracture types than those specified in the inclusion criteria, or were treated with internal fixation, preventing them from experiencing the primary outcome of reduction of a hip replacement. ASA=American Society of Anesthesiologists. DM-THR=dual mobility total hip replacement. THR=total hip replacement. VAS=visual analogue scale.

* One participant with ASA score 5 was grouped together with ASA score 4.

† Head diameters larger than 36 mm are due to inclusion of participants treated with hemiarthroplasty in the modified intention-to-treat analysis.

‡ Utility scores on the EQ-5D-5L questionnaire range from −0·594 to 1, with higher scores indicating better quality of life. Scores were calculated according to the UK index valuation. Data were available for 372 participants in the DM-THR group and 352 participants in the THR group.

§ Scores on the VAS of the EQ-5D-5L questionnaire range from 0 to 100, with higher scores indicating better quality of life. Baseline data were available for 373 participants in the DM-THR group and 357 participants in the THR group.

### Role of the funding source

The funders of the study had no role in study design, data collection, data analysis, data interpretation, or writing of the report.

## Results

Recruitment began on Jan 9, 2020 in Sweden and on Dec 6, 2022 in the UK, and was completed on April 2, 2024; final follow-up was 1 year later. Of the 2934 patients who were screened, 1334 were not enrolled. The main reasons for patients not being enrolled were surgeon preference (n=560), screening occurring after index surgery (n=257), and not consenting (n=213; figure 1). 1600 participants were thus randomly assigned, with 798 allocated to DM-THR and 802 to THR. Of these, five participants were treated with fracture fixation rather than replacement so could not experienced the primary outcome, and these individuals were excluded together with 29 participants with specified protocol deviations. Of the 1600 randomly assigned participants, 1566 (97·9%) were thus included in the mITT population and contributed to the primary analysis (figure 1, table 1). Participants had a mean age of 76·1 years (SD 6·0), 1010 (64·5%) were women, and 884 (56·4%) had an American Society of Anaesthesiologists score of 2. Table 1 shows baseline characteristics in the two groups.

35 participants crossed over from DM-THR to THR, 17 participants crossed over from THR to DM-THR, and 48 participants underwent hemiarthroplasty (figure 1, appendix 4 p 10). Surgical care was well balanced between groups; a posterior approach was used in 792 (50·6%) of all participants, cemented cup fixation in 1270 (81·1%), and cemented stem fixation in 1552 (99·1%) of all participants (table 1).

**Figure 2:**
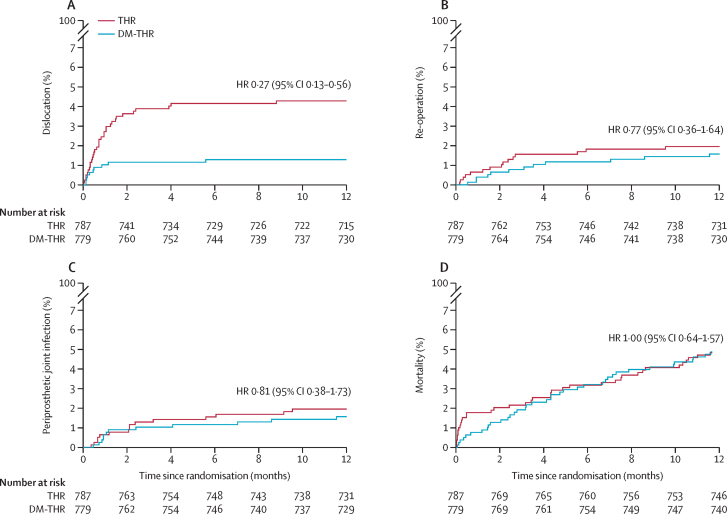
Cumulative incidence of dislocation and secondary outcomes within 1 year after randomisation Kaplan–Meier estimates for the cumulative incidence of dislocation (A), any re-operation (B), prosthetic joint infection (C), and mortality (D), in the modified intention-to-treat population according to randomised treatment within 1 year after randomisation. Numbers at risk are given below each panel, and HRs derived from adjusted Cox regression models are given with 95% CIs. DM-THR=dual mobility total hip replacement. HR=hazard ratio. THR=total hip replacement.

**Figure 3:**
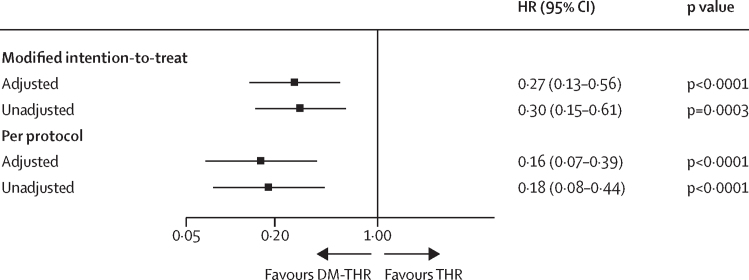
Adjusted and unadjusted HRs for the risk of dislocation within 1 year after randomisation, in the modified intention-to-treat and per-protocol populations Effects are HRs derived from Cox proportional hazard models for DM-THR as compared with THR with 95% CI. Models are adjusted for age, sex, surgical approach, and country. DM-THR=dual mobility total hip replacement. HR=hazard ratio. THR=total hip replacement.

Ten (1·3%) of 779 participants in the DM-THR group and 33 (4·2%) of 787 in the THR group required closed or open reduction of the index replacement within 1 year (figure 2A). The adjusted HR for dislocation (DM-THR *vs* THR) was 0·27 (95% CI 0·13-0·56; p<0·0001; table 2, figure 3). Analyses of the per protocol population were concordant with this finding (adjusted HR 0·16; 0·07-0·39; appendix 1 p 18), as were unadjusted analyses of the mITT and the per protocol populations (figure 3).

**Table 2:** Trial outcomes (modified intention-to-treat population)

	**DM-THR (n=779)**	**THR (n=787)**	Adjusted HR*(95% CI)	**p value**
**Primary outcome**				
Dislocation within 1 year	10 (1·3%)	33 (4·2%)	0·27 (0·13 to 0·56)	<0·0001
Dislocation treated with closed reduction	9 (1·2%)	25 (3·2%)	NA	NA
Dislocation treated with open reduction	1 (0·1%)	8 (1·0%)	NA	NA
**Secondary outcomes**				
Prosthetic joint infection within 1 year	12 (1·5%)	15 (1·9%)	0·81 (0·38 to 1·73)	0·59
Re-operation within 1 year	12 (1·5%)	15 (1·9%)	0·77 (0·36 to 1·64)	0·49
Any surgical complication within 1 year†	23 (3·0%)	48 (6·1%)	0·45 (0·27 to 0·74)	0·001
Mortality within 90 days	15 (1·9%)	17 (2·2%)	0·86 (0·43 to 1·74)	0·68
Mortality within 1 year	38 (4·9%)	38 (4·8%)	1·00 (0·64 to 1·57)	1·0
Dislocation or mortality within 1 year	48 (6·2%)	69 (8·8%)	0·66 (0·46 to 0·95)	0·026
EQ-5D-5L utility score,‡ mean (SD)	0·77 (0·20)	0·77 (0·20)	0·00 (−0·03 to 0·03)§	0·79
EQ-5D-5L VAS score,¶ mean (SD)	75·6 (17·8)	74·7 (17·9)	0·87 (0·66 to 1·14)‖	0·31

DM-THR=dual mobility total hip replacement. HR=hazard ratio. NA=not available. OR=odds ratio. THR=total hip replacement. VAS=visual analogue scale.

* The adjusted HR is for DM-THR as compared with THR with 95% CI; adjusted Cox proportional hazard models include age, sex, surgical approach, and country.

† Any surgical complication indicates that at least one of dislocation, prosthetic joint infection, or re-operation was recorded for a participant within 1 year. Not all dislocations and prosthetic joint infections resulted in re-operations, and some patients had more than one of these complications.

‡ Mean (SD) utility scores on the EQ-5D-5L questionnaire range from −0·594 to 1, with higher scores indicating better quality of life. Scores calculated according to the UK index valuation.

§ Data presented as mean difference (95% CI).

¶ Mean (SD) scores on the VAS of the EQ-5D-5L questionnaire range from 0 to 100, with higher scores indicating better quality of life.

‖ Results from proportional odds logistic regression model; odds ratios for having lower values in VAS (ie, worse outcome) in DM-THR compared with THR. Model includes baseline VAS score and randomised treatment. Baseline score was entered as numeric variable modelled as restricted cubic spline with four knots placed at 5, 35, 65, and 95 percentiles.

A re-operation of the index replacement for any indication was undertaken in 12 (1·5%) participants in the DM-THR group and in 15 (1·9%) in the THR group (adjusted HR 0·77; 95% CI 0·36-1·64; table 2, figure 2B). A prosthetic joint infection was diagnosed in 12 (1·5%) participants in the DM-THR group and in 15 (1·9%) participants in the THR group (adjusted HR 0·81; 0·38-1·73; table 2, figure 2C). After 1 year, 38 (4·9%) participants had died in the DM-THR group and 38 (4·8%) in the THR group (adjusted HR 1·0; 0·64-1·57; table 2, figure 2D). The incidence of any surgical complication (dislocation, prosthetic joint infection, or re-operation for any reason within 1 year) was 23 (3·0%) in the DM-THR group and 48 (6·1%) in the THR group (adjusted HR 0·45; 0·27-0·74; table 2). EQ-5D-5L was similar between treatment groups at 1 year (mean difference 0·0, 95% CI –0·03 to 0·03; table 2). The cumulative incidence of the composite outcome of dislocation or death within 1 year was 48 (6·2%) in the DM-THR group and 69 (8·8%) in the THR group (adjusted HR 0·66; 0·46–0·95; table 2). Analyses of the per protocol population resulted in similar estimates (appendix 1 p 18).

Prespecified subgroup analyses showed that among participants in whom a posterior surgical approach was used, ten (2·4%) in the DM-THR had a dislocation within 1 year, compared with 27 (7·0%) in the THR group. Among individuals in whom a direct lateral surgical approach was used, none of the 369 patients randomly assigned to DM-THR had a dislocation, compared with six (1·5%) of the 402 randomly assigned to THR (appendix 1 p 24).

In an exploratory, post-hoc complete case analysis we compared patient-reported outcome measures in participants who had at least one dislocation with those who did not have a dislocation. We found a mean EQ-5D-5L index of 0·644 (SD 0·264) in individuals who dislocated and 0·736 (SD 0·230) in those who did not.

## Discussion

Among participants with a displaced femoral neck fracture treated with a THR, we found that a DM-THR substantially reduced the risk of dislocation over the first year after surgery. Most dislocations did not lead to re-operations but were treated by closed reductions. The incidence of any surgical complication was lower in the group treated with DM-THR than the THR group; prosthetic joint infection and mortality were similar between treatments, as was quality of life.

Our trial was pragmatic, including a range of surgical and implant preferences in current practice, was designed with a multiplicity of sources for the primary outcome guarding against detection bias, and was conducted internationally including different hospital settings. Therefore, our findings are likely to be widely generalisable to patients with a displaced femoral neck fracture treated with THR. Participating units had been regularly using both implant types before the start of the trial, and surgeons were confident to undertake both procedures, so that adherence to allocated treatment was excellent. We selected a primary outcome that reflects patients’ concerns after THR surgery for osteoarthritis and was prioritised by our patient partners with lived experience of a femoral neck fracture.^25^ Moreover, our primary outcome was not reliant on self-report but based on registered diagnostic and procedural codes and medical chart review.

Our finding that dislocations were treated mainly with closed reduction, which is usually performed under sedation or general anaesthesia, is consistent with current surgical practice where open surgery is usually reserved for patients with recurrent dislocations.^26^, ^27^ Although surgeons were free to choose any approach they preferred, in accordance with both Swedish and UK practice only posterior and lateral approaches were used. Consistent with previous observational findings,^23^, ^28^ the risk of dislocation after THR in our cohort was higher when a posterior surgical approach was used, and, importantly, the choice of surgical approach was well balanced between treatment groups. Similarly, the use of extended femoral neck lengths, a choice of geometry of THR that is believed to reduce the risk of dislocation,^29^ was reasonably balanced across the groups. Other surgical preferences, such as the predominant use of cemented implant fixation, were in accordance with existing evidence and national guidelines.^30^, ^31^

There are limitations to our trial. As with many pragmatic randomised controlled trials, we made no attempt to mask participants or surgeons to treatment assignment.^32^ But, other than quality of life, the outcomes of our trial were objective, and the trial team was masked to allocation. The UK arm of the trial was initiated about 2 years after the Swedish study arm had started, and the UK study protocol was developed to closely match its Swedish counterpart. Nonetheless, some details—such as the reasons physicians performing the initial screening could select for not enrolling patients and the uneven availability of some variables for either the Swedish or the UK cohorts—differ. This variation, along with the lower recruitment and dislocation rates in the UK cohort, make country-level comparisons uninformative. The inclusion rate in this trial is consistent with that reported in other pragmatic surgical studies, but the variation between sites in the proportion of enrolled versus screened patients is notable. Such heterogeneity has important implications, as it can introduce selection bias and limit generalisability. The predominant use of cemented, monoblock dual mobility cups, together with the absence of alternative surgical approaches, such as the direct anterior approach, should also be recognised as important contextual factors when interpreting our findings.

Joint-specific patient-reported outcome measures, such as the Oxford Hip Score or the Western Ontario and McMaster Universities Osteoarthritis Index—typically used to measure outcome following planned THR—might have been more informative than EQ-5D alone. Since our pragmatic trial was entirely registry-nested we were constrained to the outcome set that the underlying registries routinely collect, in this case EQ-5D-5L. However, EQ-5D is the preferred general instrument for measuring recovery from hip fracture and yields similar information as the Oxford Hip Score.^33^, ^34^ No difference in EQ-5D-5L was found in our prespecified analyses; we recognise that this result could reflect a measurement limitation of the instrument in this fitter group of patients. Nonetheless, we did detect a clinically relevant difference in EQ-5D-5L index between participants who had at least one dislocation compared with those who did not, which is in line with the underlying assumptions of this trial.^6^

Participants were only followed up for 1 year; however, the majority of dislocation and prosthetic joint infection events will have presented in this window,^10^, ^27^ and the late complication of polyethylene wear might be less applicable to a hip fracture population with shorter survival than that of patients undergoing THR for osteoarthritis.^35^ Nonetheless, patients with a femoral neck fracture who undergo THR represent those with the longest anticipated life expectancy. We therefore plan for longer-term follow-up of our trial participants to detect late complications associated with polyethylene wear, such as late dislocations, osteolysis, and implant loosening.

We did not collect additional data about the details of surgical procedures, such as the mode of muscle or hip capsule repair or the use of muscle sparing approaches; patient-specific factors, such as spinopelvic mobility, as well as surgical parameters, such as the positioning of the cup and stem components, can also influence the risk of dislocation. Although such parameters can influence dislocation rates,^10^, ^23^ we have no reason to believe they were unbalanced between treatment groups and all procedures were done by surgeons familiar with these techniques and implants. We will report the radiographic outcomes in a subsequent report, but this was not a prespecified outcome in this pragmatic, registry-based trial, and the lack of such information needs to be acknowledged. There might have been differences in the co-interventions delivered between participating sites, such as rehabilitation strategies intended to improve mobilisation and gait patterns.^36^ However, we monitored all sites to ensure that interventions were in fact balanced and believe that they were unlikely to influence the effect estimate.

Few data, and no large-scale randomised trials, are available with which to compare our findings. A recent, non-randomised, registry study^37^ reported incidences for dislocation and re-operation that were similar to the findings in our trial. However, by contrast with existing non-randomised studies we found no evidence of a difference in the incidence of prosthetic joint infection between groups while demonstrating a large and clinically important benefit in reducing dislocations. It is likely that existing observational studies have residual confounding by indication, which might explain the variance in findings. An ongoing, randomised trial of the same research question will contribute important data within the next few years.^38^ DM-THR implants are roughly twice as costly as standard THR in many health-care systems, but implant prices represent only one element of overall cost-effectiveness. It is possible that DM-THR could be a cost-effective intervention in some jurisdictions;^39^, ^40^ and a formal health-economic evaluation based on this trial is therefore currently underway.

DM-THR is highly effective compared with standard THR in reducing the risk of dislocations, the most common postoperative surgical complication after this procedure, and it is also effective in reducing the risk of other surgical complications. DM-THR, when available, should therefore be considered for patients with a displaced femoral neck fracture undergoing a THR.

### Contributors

### Data sharing

Based on the Swedish Patient Act, sensitive data can only be shared after approval of the Swedish National Review Authority. All data requests should be submitted to the corresponding author for further consideration.

## Declaration of interests

NPH is Director of the national infrastructure Biobank Sweden funded by the Swedish Research Council; is a member of the steering committee of the Swedish Arthroplasty Register; and declares personal payments for lectures from Waldemar Link and Heraeus, unrelated to this study. OW is Director of the Swedish Fracture register. SEL is supported by the National Institute for Health and Care Research (NIHR) Exeter Biomedical Research Centre (BRC). DA acknowledges support from NIHR Oxford BRC. XLG acknowledges support from NIHR Barts BRC (NIHR203330). All other authors declare no competing interests.
