# Supplementary material for: Dual mobility versus standard cups in total hip replacement for displaced femoral neck fractures (Duality): an international, multicentre, randomised, controlled, superiority trial
Source: Lancet. 2026 Jul 25;408(10552):348–56. doi: 10.1016/S0140-6736(26)00759-2 (PMC13425373; doi:10.1016/S0140-6736(26)00759-2)

THE LANCET

Supplementary appendix 1

This appendix formed part of the original submission. We post it as supplied by the authors.

Supplement to: Hailer NP, Griffin XL, Mukka S, et al. Dual mobility versus standard cups in total hip replacement for displaced femoral neck fractures (Duality): an international, multicentre, randomised, controlled, superiority trial. *Lancet* 2026; published online July 2. https://doi.org/10.1016/S0140-6736(26)00759-2.

Appendix 1

TRIAL OVERSIGHT **2**

OVERSIGHT COMMITTEES 2

DATA SAFETY MONITORING COMMITTEES 3

TRIAL MANAGEMENT COMMITTEES 4

THE DUALITY INVESTIGATORS **6**

REGISTER MANAGEMENT 6

STUDY COORDINATORS 6

LOCAL STUDY REPRESENTATIVES 7

APPENDIX TABLES **10**

APPENDIX TABLE 1: PARTICIPANT DISPOSITION IN THE MODIFIED INTENTION-TO-TREAT POPULATION AND IN THE PER-PROTOCOL POPULATION. 10

APPENDIX TABLE 2. IMPLANT CHARACTERISTICS BY RANDOMISED TREATMENT (MODIFIED INTENTION-TO-TREAT POPULATION). 12

APPENDIX TABLE 3. PARTICIPANT DEMOGRAPHIC CHARACTERISTICS BY RANDOMISED TREATMENT (PER-PROTOCOL POPULATION). 14

APPENDIX TABLE 4. IMPLANT CHARACTERISTICS BY RANDOMISED TREATMENT (PER-PROTOCOL POPULATION). 16

APPENDIX TABLE 5. TRIAL OUTCOMES (PER-PROTOCOL POPULATION). 18

APPENDIX TABLE 6. DISLOCATIONS BY RANDOMISED TREATMENT AND PER COMPONENT (MODIFIED INTENTION-TO-TREAT POPULATION) 20

APPENDIX TABLE 7. DISLOCATIONS BY RANDOMISED TREATMENT AND PER COMPONENT (PER-PROTOCOL POPULATION). 21

APPENDIX FIGURES **22**

APPENDIX FIGURE 1: CUMULATIVE INCIDENCE OF DISLOCATION AND OTHER SECONDARY OUTCOMES WITHIN ONE YEAR AFTER RANDOMISATION IN THE PER-PROTOCOL POPULATION. 22

APPENDIX FIGURE 2: SUBGROUP ANALYSES OF THE RISK FOR DISLOCATION IN THE MODIFIED INTENTION-TO-TREAT POPULATION. 24

Trial Oversight

Oversight Committees

**Sweden**	
Nils P. Hailer	Department of Surgical Sciences/Orthopaedics & Hand Surgery, Uppsala University, Uppsala
Paul Gerdhem	Department of Surgical Sciences/Orthopaedics & Hand Surgery, Uppsala University, Uppsala
Per Morberg	Department of Surgical and Perioperative Sciences, Umeå University, Umeå
Sebastian Mukka	Department of Diagnostics and Intervention (Orthopaedics), Umeå University, Umeå
Michael Möller	Department of Orthopaedics, Sahlgrenska University Hospital Gothenburg/Mölndal
Cecilia Rogmark	Department of Orthopedics, Skåne University Hospital, Malmö
Ola Rolfsson	Department of Orthopaedics, Sahlgrenska University Hospital Gothenburg/Mölndal
Jörg Schilcher	Department of Orthopaedics, Linköping University Hospital
Monica Sjöholm	Department of Surgical Sciences, Uppsala University
Olof Sköldenberg	Department of Clinical Sciences, Danderyd Hospital, Unit of Orthopaedics | Karolinska Institute, Stockholm
Olof Wolf	Department of Surgical Sciences/Orthopaedics & Hand Surgery, Uppsala University, Uppsala

UK	
Andrew Duckworth	Centre for Population Health Sciences<br>Usher Institute, University of Edinburgh, Edinburgh<br>UK
Sue Jowett	Department of Applied Health Sciences, University of Birmingham, Birmingham, UK
Alex Mitchell	York Trials Unit, University of York, York, UK
Josephine Rowling	Patient Representative, UK
Nicola Greenlaw	Robertson Centre for Biostatistics, University of Glasgow, Glasgow, UK
Andrew Stoddart	Edinburgh Clinical Trials Unit<br>Usher Institute, Edinburgh, UK

Data safety monitoring committees

Sweden	
Paul Ackermann	Karolinska Institute, Stockholm
Michael Haney	Umeå University, Umeå
Jakob Hedberg	Uppsala University, Uppsala

UK	
Rudolf Poolman	Leiden University, Leiden, Netherlands
Kelly Handley	Birmingham Clinical Trials Unit, University of Birmingham, Birmingham, UK
Opinder Sahota	Nottingham Ortho-Geriatric Unit, Nottingham University Hospitals, Nottingham, UK
Versha Cheed	Department of Applied Health Sciences, University of Birmingham, Birmingham, UK

Trial management committees

**Sweden**	
Nils P. Hailer	Department of Surgical Sciences/Orthopaedics & Hand Surgery, Uppsala University, Uppsala
Sebastian Mukka	Department of Diagnostics and Intervention (Orthopaedics), Umeå University, Umeå
Olof Wolf	Department of Surgical Sciences/Orthopaedics & Hand Surgery, Uppsala University, Uppsala

**UK**	
Xavier Griffin	Bone and Joint Health, Blizard Institute, Queen Mary University of London, London
Michael Whitehouse	Musculoskeletal Research Unit, University of Bristol, Bristol
Matthew Costa	Oxford Trauma & Emergency Care, NDORMS, University of Oxford, Oxford
Juul Achten	Oxford Trauma & Emergency Care, NDORMS, University of Oxford, Oxford
Richard Grant	Lead Patient Representative
Rory Middleton	Department of Trauma and Orthopaedics, Royal Cornwall Hospital, Truro
Jonathan Cook	NDORMS, University of Oxford, Oxford
Borislava Mihaylova	Health Economics and Policy Research Unit, Wolfson Institute of Population Health, Queen Mary University of London, London
Esubalew Assefa	Health Economics and Policy Research Unit, Wolfson Institute of Population Health, Queen Mary University of London, London
Duncan Appelbe	Oxford Trauma & Emergency Care, NDORMS, University of Oxford, Oxford

Amrita Athwal	Oxford Trauma & Emergency Care, NDORMS, University of Oxford, Oxford
Sarah (Sallie) Lamb	Faculty of Health and Life Sciences, University of Exeter, Exeter
Rhys Painton	Oxford Trauma & Emergency Care, NDORMS, University of Oxford, Oxford
Jamie Stokes	Leeds Clinical Trials Research Unit, University of Leeds

The DUALITY Investigators

Register Management

Name	Affiliation
Ola Rolfson	Department of Orthopaedics, Sahlgrenska University Hospital, Gothenburg/Mölndal
Michael Möller	Department of Orthopaedics, Sahlgrenska University Hospital, Gothenburg/Mölndal
Johan Kärrholm	Department of Orthopaedics, Sahlgrenska University Hospital, Gothenburg/Mölndal
Krister Arlinger	Centre of Registers Västra Götaland, Gothenburg
Hanne Carlsen	Centre of Registers Västra Götaland, Gothenburg

Study coordinators

Name	Affiliation
Monica Sjöholm	Department of Surgical Sciences, Uppsala University, Uppsala
Ann-Charlotte Claesson	Department of Surgical Sciences, Uppsala University, Uppsala

Local study representatives

Name	Affiliation
Frida Boström<br>Konstantinos Papadopoulos	Department of Orthopaedics, Skellefteå Hospital, Skellefteå
Rasmus Bjerre	Department of Orthopaedics, Kalmar County Hospital
Hans-Peter Bögl	Department of Orthopaedics, Gävle Hospital, Gävle
Christian Hellerfelt	Department of Orthopaedics, Blekinge Hospital, Karlskrona
Anders Isacsson	Department of Orthopaedics, Department of Orthopaedics, Helsingborg Hospital
Per-Erik Johanson	Department of Orthopaedics, Borås Hospital
Nicole Jessen	Department of Orthopaedics, Sunderby Hospital, South Sunderbyn, Norrbotten County
Kamal Kadum<br>Maziar Mohaddes	Department of Orthopaedics, Sahlgrenska University Hospital, Gothenburg/Mölndal
Daniel Stam	Department of Orthopedics, Halland Hospital, Halmstad
Maria Mannberg	Department of Orthopaedics & Hand Surgery, Uppsala University Hospital, Uppsala
Maja Notini	Department of Orthopaedics, Nyköping Hospital, Nyköping
Elin Nemlander	Department of Orthopaedics, Ljungby Hospital
Linnea Nyström	Department of Orthopaedics, Västmanland Hospital Västerås
Jörg Schilcher	Department of Orthopedics, Linköping University Hospital
Olof Sköldenberg	Department of Clinical Sciences, Danderyd Hospital, Unit of Orthopaedics | Karolinska Institutet, Stockholm

Gustav Trehn	Department of Orthopaedics, Östersund Hospital, Östersund
Nils von Wachenfelt<br>René Notelid	Department of Orthopaedics, Region Jönköping County, Eksjö Hospital
Sebastian Mukka<br>Jonas Sundkvist	Department of Diagnostics and Intervention (Orthopaedics), Umeå University, Umeå
Vasileios Zampelis<br>Gunnar Flivik<br>Cecilia Rogmark	Departments of Orthopedics and Clinical Sciences, Skåne University Hospital, Lund
Lotta Kettil	Department of Orthopaedics, Skaraborgs Hospital, Lidköping
Rory Middleton	Royal Cornwall Hospital
Christopher Buckle	Conquest Hospital
Gareth Roberts	University Hospital of Wales
Konstantinos Tsitskaris	Whipps Cross Hospital
Sam Heaton	Yeovil District Hospital
Ian Dos Remedios	Royal Stoke University Hospital
William Poole	Southmead Hospital
Aaron Ng	Pinderfields Hospital
Rathan Yarlagadda	Derriford Hospital
Andrew McAndrew	Royal Berkshire Hospital
Andrew Kelly	Musgrove Park Hospital
Saif Ul Islam	Aintree University Hospital
Marshall Sangster	Gloucestershire Royal Hospital
Mike Reed	Northumbria Specialist Emergency Care Hospital
Kieran Gallagher	Poole General Hospital
Owen Diamond	Royal Victoria Hospital Belfast

Paul Magill	Craigavon Area Hospital
Ayman Sorial	Peterborough City Hospital
Doug Dunlop	Southampton General Hospital
James Berstock	Royal United Hospital Bath
Matt Gee	King's College Hospital
Benjamin Kapur	Glan Clwyd Hospital
Theophilus Joachim	Pilgrim Hospital
Girish Vashista	Donacaster Royal Infirmary

Appendix tables

Appendix table 1: Participant disposition in the modified intention-to-treat population and in the per-protocol population.

	DM-THR	THR	Total
**Participants randomised in the trial**	**798**	**802**	**1600**
	n (%)	n (%)	n (%)
**Participants excluded from the modified intention-to-treat population** *	**19 (2·4%)**	**15 (1·9%)**	**34 (2·1%)**
- due to cognitive impairment **	1 (5·3%)	3 (20·0%)	4 (11·8%)
- due to absence of fracture **	1 (5·3%)		1 (2·9%)
- due to lack of documented informed consent **	4 (21·1%)	4 (26·7%)	8 (23·5%)
- due to treatment with internal fixation **	3 (15·8%)	2 (13·3%)	5 (14·7%)
- due to withdrawn consent **	1 (5·3%)		1 (2·9%)
- due to wrong fracture type (intertrochanteric) **	9 (47·4%)	6 (40·0%)	15 (44·1%)
**Participants included in modified intention-to-treat population***	**779 (97·6%)**	**787 (98·1%)**	**1566 (97·9%)**
**Participants excluded from per-protocol population***	**76 (9·5%)**	**58 (7·2%)**	**134 (8·4%)**
- due to exclusion from modified intention-to-treat population ***	19 (25·0%)	15 (25·9%)	34 (25·4%)
- due to cross-over***	35 (46·1%)	17 (29·3%)	52 (38·8%)
- due to treatment with hemiarthroplasty ***	22 (28·9%)	26 (44·8%)	48 (35·8%)
**Participants included in per-protocol population***	**722 (90·5%)**	**744 (92·8%)**	**1466 (91·6%)**

* the denominator is the number of randomised subjects per treatment group.

[**]the denominator is the number of subjects excluded from the modified intention-to-treat population per treatment group.

[***]the denominator is the number of subjects excluded from the per-protocol population per treatment group.

Appendix table 2. Implant characteristics by randomised treatment (modified intention-to-treat population).

The types of cups, stems, and cement used for fixation of either component, divided by randomised treatment groups.

Implant Characteristic		DM-THR (n=779)	THR (n=787)
Type of cup*, n (%)**	Avantage	310 (39·8%)	8 (1·0%)
	BI-MENTUM	28 (3·6%)	
	BiMobile	152 (19·5%)	4 (0·5%)
	Continuum	1 (0·1%)	5 (0·6%)
	Exeter RimFit	6 (0·8%)	158 (20·1%)
	G7	11 (1·4%)	1 (0·1%)
	IP	3 (0·4%)	65 (8·3%)
	Lubinus	10 (1·3%)	347 (44·1%)
	Marathon	2 (0·3%)	53 (6·7%)
	MobiliT	17 (2·2%)	
	Novae	33 (4·2%)	4 (0·5%)
	Pinnacle	5 (0·6%)	39 (5·0%)
	Polar R3		3 (0·4%)
	Polar cup	114 (14·6%)	1 (0·1%)
	Trident	56 (7·2%)	38 (4·8%)
	Trilogy	1 (0·1%)	19 (2·4%)
	Trinity		15 (1·9%)
	Other	8 (1·0%)	1 (0·1%)
	Hemiarthroplasty	22 (2·8%)	26 (3·3%)
Type of cup cement, n (%)	Cementless	146 (18·7%)	102 (13·0%)
	Copal	118 (15·1%)	99 (12·6%)
	Optipac	267 (34·3%)	286 (36·3%)
	Palacos	195 (25·0%)	251 (31·9%)
	Refobacin	21 (2·7%)	15 (1·9%)
	Other	6 (0·8%)	4 (0·5%)
	Hemi or missing	26 (3·3%)	30 (3·8%)

Type of stem, n (%)	C-Stem	14 (1·8%)	15 (1·9%)
	CPT	34 (4·4%)	31 (3·9%)
	Corail	3 (0·4%)	3 (0·4%)
	Exeter	131 (16·8%)	123 (15·6%)
	MS-30	68 (8·7%)	71 (9·0%)
	SPII	518 (66·5%)	527 (67·0%)
	Taperfit	8 (1·0%)	10 (1·3%)
	Other	3 (0·4%)	7 (0·9%)
Type of stem cement, n (%)	Cementless	4 (0·5%)	10 (1·3%)
	Copal	144 (18·5%)	115 (14·6%)
	Optipac	296 (38·0%)	306 (38·9%)
	Palacos	299 (38·4%)	327 (41·6%)
	Refobacin	19 (2·4%)	19 (2·4%)
	Simplex	8 (1·0%)	6 (0·8%)
	Other	4 (0·5%)	1 (0·1%)
	Missing	5 (0·6%)	3 (0·4%)

* The presence of DM cups in the THR group and of standard cups in the DM-THR group are due to cross-over, as described in figure 1 and in appendix table 1.

** Percentages may not total 100 because of rounding.

Appendix table 3. Participant demographic characteristics by randomised treatment (per-protocol population).

The per-protocol population included 1466 participants. Of the 1600 enrolled participants, 34 were already excluded from the modified intention-to-treat population (please refer to figure 1 and appendix table 1 for details), and 52 who had received a different cup type than they were allocated to, and 48 who were treated with a hemiarthroplasty, were additionally excluded from the modified intention-to-treat population.

Characteristic		DM-THR (n= 722)	THR (n= 744)
Age in years, mean (SD)*		76·0 ± 5·9	76·0 ± 6·0
Sex, n (%)	Female	457 (63·3%)	488 (65·6%)
	Male	265 (36·7%)	256 (34·4%)
Country, n (%)	Sweden	624 (86·4%)	637 (85·6%)
	UK	98 (13·6%)	107 (14·4%)
Fracture type, n (%)	Femoral neck fracture (AO 31-B3)	696 (96·4%)	699 (94·0%)
	Basocervical fracture (AO 31-B2)	26 (3·6%)	45 (6·0%)
Body mass index		24·8 ± 4·2	24·8 ± 4·1
ASA score, n (%)	1	60 (8·3%)	73 (9·8%)
	2	407 (56·4%)	426 (57·3%)
	3	237 (32·8%)	231 (31·0%)
	4**	8 (1·1%)	8 (1·1%)
	Missing	10 (1·4%)	6 (0·8%)
Surgical approach, n (%)	Direct lateral	338 (46·8%)	382 (51·3%)
	Posterior	383 (53·0%)	361 (48·5%)
	Missing	1 (0·1%)	1 (0·1%)

Type of replacement, n (%)	Cemented THR	585 (81·0%)	641 (86·2%)
	Hybrid THR	133 (18·4%)	94 (12·6%)
	Reverse hybrid THR	2 (0·3%)	1 (0·1%)
	Cementless THR	2 (0·3%)	8 (1·1%)
Cup diameter (mm), median (range)		50 (38 - 66)	50 (42 - 64)
Femoral head diameter (mm), median (range)		28 (22 - 32)	32 (22 - 36)
Femoral neck length, n (%)	Standard	553 (76·6%)	561 (75·4%)
	Extended	141 (19·5%)	134 (18·0%)
	Extra extended	5 (0·7%)	15 (2·0%)
	Missing	23 (3·2%)	34 (4·6%)
EQ-5D-5L Utility score***, mean (SD)		0·82 ± 0·19	0·82 ± 0·22
EQ-5D-5L VAS score****, mean (SD)		77·1 ± 19·4	76·5 ± 20·4

* Data are n (%), mean (±SD), or median (range). Percentages may not total 100 because of rounding.

** One participant with ASA class 5 was grouped together with ASA class 4.

*** Utility scores on the EuroQol Group 5-Dimension (EQ-5D-5L) questionnaire range from −0.594 to 1, with higher scores indicating better quality of life. Scores calculated according to the UK index valuation. Data were available on 348 patients in the DM-THR group and on 342 patients in the THR group.

**** Scores on the visual analogue scale (VAS) of the EQ-5D-5L questionnaire range from 0 to 100, with higher scores indicating better quality of life. Data were available on 349 patients in the DM-THR group and on 347 patients in the THR group.

Appendix table 4. Implant characteristics by randomised treatment (per-protocol population).

The types of cups, stems, and cement used for fixation of either component, divided by randomised treatment groups. The per-protocol population included 1466 participants. Of the 1600 enrolled participants, 34 were already excluded from the modified intention-to-treat population (please refer to figure 1 and appendix table 1 for details), and 52 who had received a different cup type than they were allocated to, and 48 who were treated with a hemiarthroplasty, were additionally excluded from the modified intention-to-treat population.

**Implant Characteristic**		**DM-THR (n=722)**	**THR (n=744)**
Type of cup, n (%)*	Avantage	310 (42·9%)	
	BI-MENTUM	28 (3·9%)	
	BiMobile	152 (21·1%)	
	Continuum		5 (0·7%)
	Exeter RimFit		158 (21·2%)
	G7	11 (1·5%)	1 (0·1%)
	IP		65 (8·7%)
	Lubinus		347 (46·6%)
	Marathon		53 (7·1%)
	MobiliT	17 (2·4%)	
	Novae	33 (4·6%)	
	Pinnacle		39 (5·2%)
	Polar R3		3 (0·4%)
	Polar cup	114 (15·8%)	
	Trident	49 (6·8%)	38 (5·1%)
	Trilogy		19 (2·6%)
	Trinity		15 (2·0%)
	Other	8 (1·1%)	1 (0·1%)

Type of cup cement, n (%)	Cementless	135 (18·7%)	102 (13·7%)
	Copal	116 (16·1%)	94 (12·6%)
	Optipac	255 (35·3%)	276 (37·1%)
	Palacos	187 (25·9%)	249 (33·5%)
	Refobacin	21 (2·9%)	15 (2·0%)
	Other	5 (0·7%)	4 (0·5%)
	Missing	3 (0·4%)	4 (0·5%)
Type of stem, n (%)	C-Stem	11 (1·5%)	14 (1·9%)
	CPT	30 (4·2%)	31 (4·2%)
	Corail	3 (0·4%)	3 (0·4%)
	Exeter	118 (16·3%)	121 (16·3%)
	MS-30	65 (9·0%)	67 (9·0%)
	SPII	484 (67·0%)	494 (66·4%)
	Taperfit	8 (1·1%)	9 (1·2%)
	Other	3 (0·4%)	5 (0·7%)
Type of stem cement, n (%)	Cementless	4 (0·6%)	9 (1·2%)
	Copal	132 (18·3%)	103 (13·8%)
	Optipac	272 (37·7%)	287 (38·6%)
	Palacos	281 (38·9%)	317 (42·6%)
	Refobacin	18 (2·5%)	18 (2·4%)
	Simplex	7 (1·0%)	6 (0·8%)
	Other	4 (0·6%)	1 (0·1%)
	Missing	4 (0·6%)	3 (0·4%)

* Percentages may not total 100 because of rounding.

Appendix table 5. Trial outcomes (per-protocol population).

The per-protocol population included 1466 participants. Of the 1600 enrolled participants, 34 were already excluded from the modified intention-to-treat population (please refer to figure 1 and appendix table 1 for details), and 52 who had received a different cup type than they were allocated to, and 48 who were treated with a hemiarthroplasty, were additionally excluded from the modified intention-to-treat population.

Outcomes		DM-THR n=722	THR n=744	Hazard ratio* (95% CI%)
		n (%)		
Secondary outcomes	Dislocation within 1 year	6 (0·8%)	33 (4·4%)	0·16 (0·07–0·39)
	…treated with closed reduction	5 (0·7%)	25 (3·4%)	
	…treated with open reduction	1 (0·1%)	8 (1·1%)	
	Prosthetic joint infection within 1 year	11 (1·5%)	13 (1·7%)	0·89 (0·40–1·98)
	Reoperation within 1 year	11 (1·5%)	13 (1·7%)	0·82 (0·37–1·83)
	Any complication**	18 (2·5%)	46 (6·2%)	0·37 (0·21–0·63)
	Mortality within 90 days	13 (1·8%)	14 (1·9%)	0·92 (0·43–1·97)
	Mortality within 1 year	32 (4·4%)	32 (4·3%)	1·01 (0·62–1·66)
	Dislocation or mortality within 1 year	38 (5·3%)	63 (8·5%)	0·57 (0·38–0·85)
				**Mean difference (95% CI%)**
	EQ-5D-5L Utility score***, mean (SD)	0·78 (0·20)	0·77 (0·20)	0·01 (−0·02–0·04)
				**Odds ratio (95% CI)**
	EQ-5D-5L VAS score****, mean (SD)	75·7 ± 17·9	74·8 ± 17·9	0·86 (0·65–1·14)

* The adjusted hazard ratio is for DM-THR as compared with THR; with 95% confidence interval (CI), adjusted Cox proportional hazard models include age, sex, surgical approach and country.

** ”Any complication” indicates that at least one of dislocation, prosthetic joint infection, or reoperation was recorded for a patient within one year. Not all dislocations and prosthetic joint infections resulted in reoperations.

*** Mean (± SD) utility scores on the EuroQol Group 5-Dimension (EQ-5D-5L) questionnaire range from −0.594 to 1, with higher scores indicating better quality of life. Scores calculated according to the UK index valuation. Data were available on 305 patients in the DM-THR group and on 308 patients in the THR group. Treatment effect estimates from linear regression model with robust standard errors, presented as difference in model adjusted means. Observed cases analyses, all models include baseline index and randomised treatment.

**** Mean (± SD) scores on the visual analogue scale (VAS) of the EQ-5D-5L questionnaire range from 0 to 100, with higher scores indicating better quality of life. Data at both baseline and at the 1-year follow-up were available on 304 patients in the DM-THR group and on 306 patients in the THR group. Results from proportional odds logistic regression model. Odds ratios (OR) for having lower values in VAS (i.e., worse outcome) in DM-THR compared to THR. Model includes baseline VAS score and randomised treatment. Observed cases used both for outcome and baseline score.

Appendix table 6. Dislocations by randomised treatment and per component (modified intention-to-treat population).

Dislocations by randomised treatment, per femoral head diameter and per cup diameter in the modified intention-to-treat population. Only variable levels with events are shown.

Component	Diameter (mm)	DM-THA Events/Total (%)	THA Events/Total (%)
**Femoral head**	22	5/72 (6·9)	0/4 (0·0)
	28	2/647 (0·3)	3/56 (5·4)
	32	2/26 (7·7)	29/579 (5·0)
	36	1/6 (16·7)	1/126 (0·8)
**Cup**	44	1/18 (5·6)	3/22 (13·6)
	45	1/11 (9·1)	0/18 (0·0)
	46	2/76 (2·6)	6/124 (4·8)
	48	0/111 (0·0)	8/148 (5·4)
	50	1/125 (0·8)	6/154 (3·9)
	52	1/104 (1·0)	3/126 (2·4)
	53	1/21 (4·8)	0/7 (0·0)
	54	1/86 (1·2)	4/84 (4·8)
	56	0/32 (0·0)	1/41 (2·4)
	58	1/13 (7·7)	1/7 (14·3)
	60	0/8 (0·0)	1/5 (20·0)
	62	1/3 (33·3)	0/1 (0·0)

Appendix table 7. Dislocations by randomised treatment and per component (per-protocol population).

Dislocations by randomised treatment, per femoral head diameter and per cup diameter in the per-protocol population. Only variable levels with events are shown.

Component	Diameter (mm)	DM-THA Events/Total (%)	THA Events/Total (%)
**Femoral head**	22	4/70 (5·7)	0/2 (0·0)
	28	2/637 (0·3)	3/37 (8·1)
	32	0/3 (0·0)	29/579 (5·0)
	36		1/126 (0·8)
**Cup**	44	1/18 (5·6)	3/21 (14·3)
	45	1/10 (10·0)	0/17 (0·0)
	46	2/75 (2·7)	6/122 (4·9)
	48	0/105 (0·0)	8/143 (5·6)
	50	0/115 (0·0)	6/153 (3·9)
	52	1/97 (1·0)	3/125 (2·4)
	53	1/21 (4·8)	0/7 (0·0)
	54	0/79 (0·0)	4/82 (4·9)
	56	0/32 (0·0)	1/41 (2·4)
	58	0/12 (0·0)	1/7 (14·3)
	60	0/8 (0·0)	1/5 (20·0)

Appendix figures

Appendix figure 1: Cumulative incidence of dislocation and other secondary outcomes within one year after randomisation in the per-protocol population.

Kaplan-Meier estimates for the cumulative incidence of (A) dislocation, (B) any re-operation, (C) prosthetic joint infection, (D) death, according to randomised treatment within one year after randomisation. Numbers at risk are given below each panel.

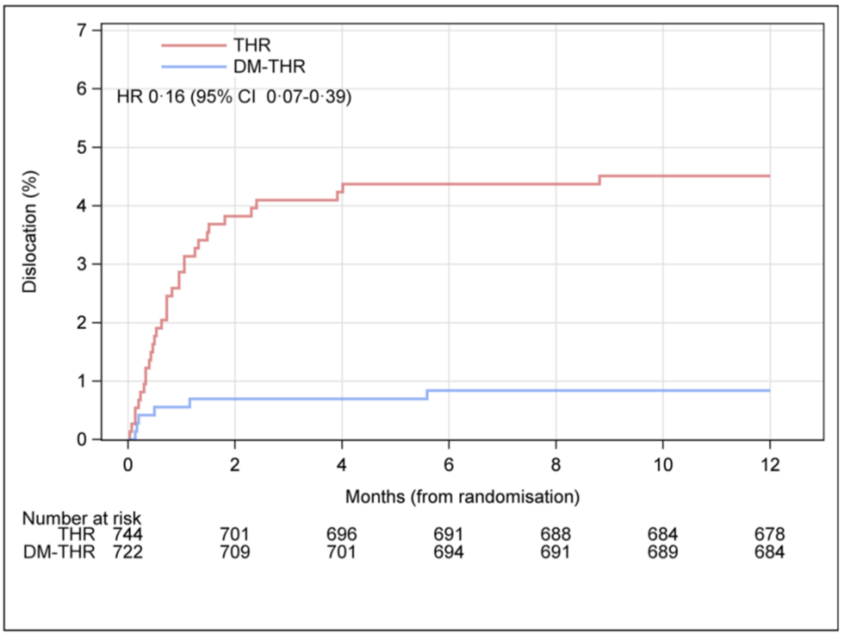

A) Dislocation

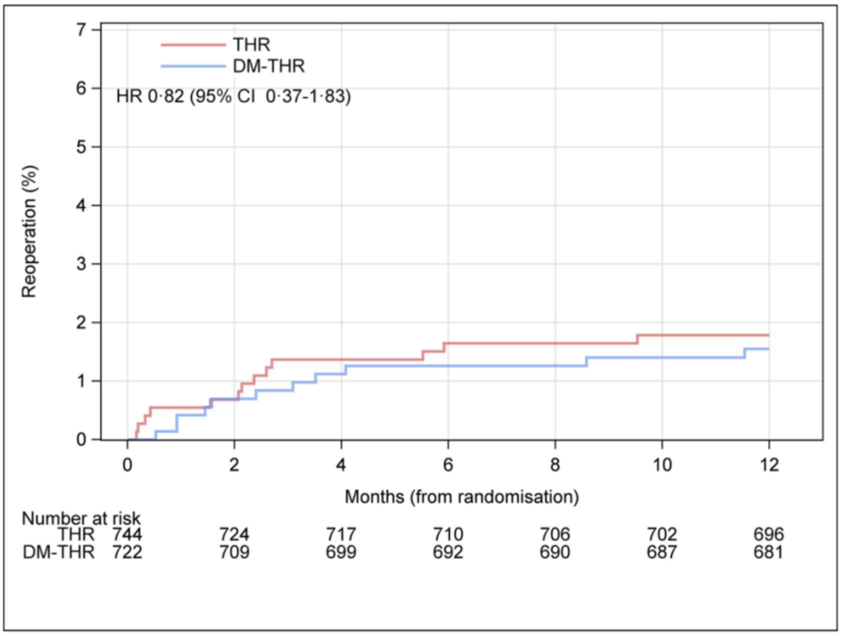

B) Any reoperation

Appendix figure 1, cont'd.

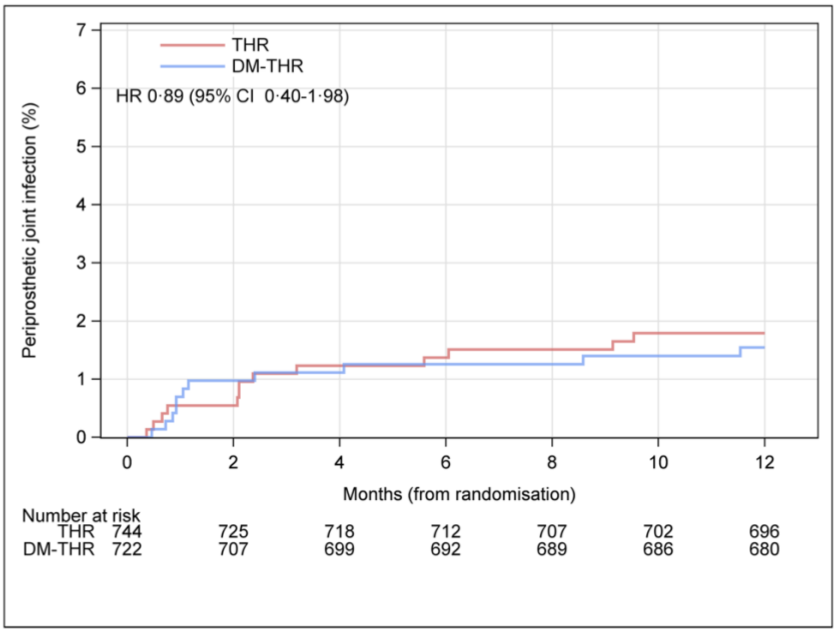

C) Prosthetic joint infection (PJI).

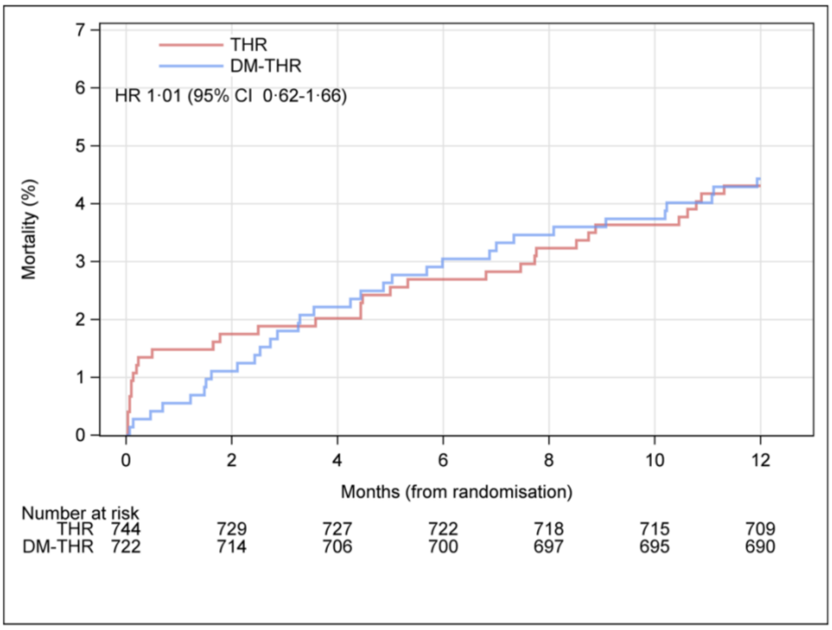

D) Mortality.

Appendix figure 2: Subgroup analyses of the risk for dislocation in the modified intention-to-treat population.

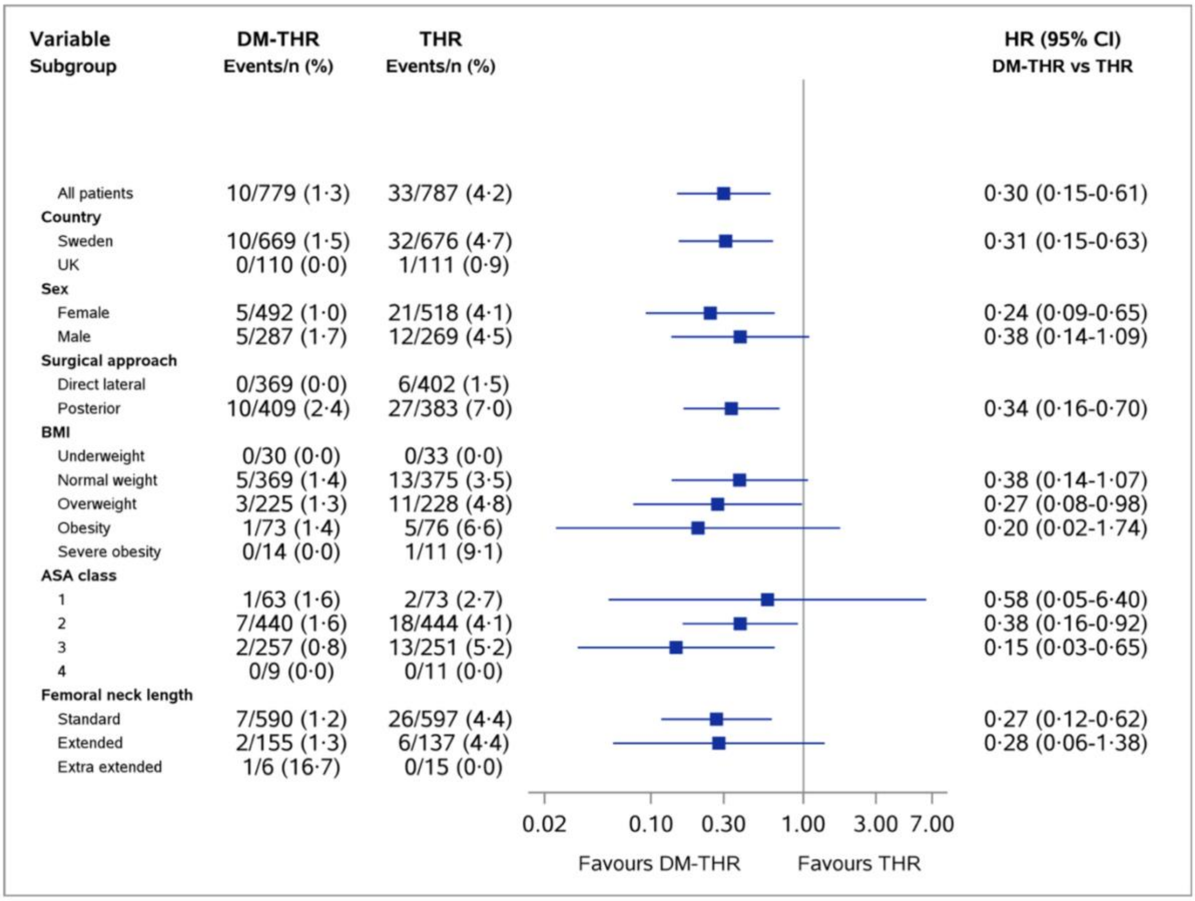

Supplement: Supplementary appendix [file mmc1.pdf]
