# Supplementary material for: Dual mobility versus standard cups in total hip replacement for displaced femoral neck fractures (Duality): an international, multicentre, randomised, controlled, superiority trial
Source: Lancet. 2026 Jul 25;408(10552):348–56. doi: 10.1016/S0140-6736(26)00759-2 (PMC13425373; doi:10.1016/S0140-6736(26)00759-2)
Supplement: Supplementary appendix [file mmc2.pdf]

THE LANCET

Supplementary appendix 2

This appendix formed part of the original submission. We post it as supplied by the authors.

Supplement to: Hailer NP, Griffin XL, Mukka S, et al. Dual mobility versus standard cups in total hip replacement for displaced femoral neck fractures (Duality): an international, multicentre, randomised, controlled, superiority trial. *Lancet* 2026; published online July 2. https://doi.org/10.1016/S0140-6736(26)00759-2.

CLINICAL STUDY PROTOCOL

The DUALITY trial - a Register-based, Randomised Controlled Trial to investigate Dual Mobility Cups in Hip Fracture Patients

**Clinical trials identifier**	NCT03909815
**Version Number:**	3.3
**Date:**	2025-02-26
**Research Body:**	Region Uppsala
**Principal investigator(s):**	Nils Hailer, Olof Wolf, Sebastian Mukka

SYNOPSIS

Title: DUALITY - a Register-based, Randomised Controlled Trial to Investigate Dual Mobility Cups in Hip Fracture Patients

Rational for conducting the study: The aims of the trial are to investigate whether the risk of dislocations after THA surgery performed due to femoral neck fracture is reduced after the use of this device, and whether other complications such as loosening or infection are more frequent. Our ***primary outcome variable*** is the occurrence of dislocations treated by closed or open reductions of the index joint within one year after the index THA procedure. ***Secondary outcome variables*** include the occurrence of re-operations for any reason, periprosthetic joint infections, mortality, patient-reported outcomes, and health care costs.

Study design: Register-based, randomised controlled trial

Study population: Patients aged ≥65 years

Number of patients: 1,600

Inclusion criteria:
- Written informed consent
- Age ≥65 years
- Diagnosis: displaced femoral neck fracture type AO 31-B2 or B3/Garden type 3 or 4
- Eligible for THA according to local guidelines and routines

Exclusion criteria:
- Previous inclusion of contralateral hip
- Delayed fracture surgery (date of injury >7 days prior to date of randomization)
- Pathological or stress fracture of the femoral neck, or fracture adjacent to a previous ipsilateral hip implant
- Inability or unwillingness to give written consent
- Dementia (as diagnosed by the screening physician)
- Unavailability of both interventions for a study subject (e.g., implants being out of stock, or lack of the individual surgeon's expertise to perform either procedure)

Primary outcome variables and examinations:
The primary outcome will be occurrence of closed or open reduction of the index joint within one year.

Trial period: Jan 1st 2020 - Dec 31st 2023

SIGNATURE PAGE

I confirm that I have read and understood this protocol and that I will work according to the protocol. By my signature, I agree to personally supervise the conduct of this study in my affiliation and to ensure its conduct in compliance with the protocol, informed consent, IRB/EC procedures, the Declaration of Helsinki, and local regulations governing the conduct of clinical studies.

Signature Principal Investigator

2025-02-26
Date (yyyy-mm-dd)

Nils Hailer
Printed name of Principal Investigator

Signature Head of Department

2025-02-26
Date (yyyy-mm-dd)

Caroline Sköld
Printed name of Head of Department

Copy for study site; to remain with study protocol

SIGNATURE PAGE

I confirm that I have read and understood this protocol and that I will work according to the protocol. By my signature, I agree to personally supervise the conduct of this study in my affiliation and to ensure its conduct in compliance with the protocol, informed consent, IRB/EC procedures, the Declaration of Helsinki, and local regulations governing the conduct of clinical studies.

Signature Principal Investigator	2025-02-26<br>Date (yyyy-mm-dd)

Nils Hailer

Printed name of Principal Investigator

Signature Head of Department	2025-02-26<br>Date (yyyy-mm-dd)

Caroline Sköld

Printed name of Head of Department

Copy for study group; to be kept by study coordinator

CONTACT DETAILS

Role	Name	Address	Phone	E-mail
Research body	Uppsala University Hospital	Department of Orthopaedics & Hand surgery, Uppsala University Hospital, 751 85 Uppsala, Sweden	018-611 0000	Caroline.skold@akademiska.se
Principal Investigator	Nils Hailer	Department of Surgical Sciences/Orthopaedics Uppsala University, 752 36 Uppsala, Sweden	070-72 48 701	nils.hailer@surgsci.uu.se
Co-Investigator	Olof Wolf	Department of Surgical Sciences/Orthopaedics Uppsala University, 752 36 Uppsala, Sweden	0709-72 82 84	olof.wolf@surgsci.uu.se
Co-Investigator	Sebastian Mukka	Ortopedkliniken, Norrlands Univ.sjukhus, Daniel Naezéns väg, 907 37 Umeå, Sweden	073-086 71 11	sebastian.mukka@umu.se
Study Coordinator	Monica Sjöholm	Department of Surgical Sciences/Orthopaedics Uppsala University, 752 36 Uppsala, Sweden	0704-25 00 43	monica.sjoholm@surgsci.uu.se

TABLE OF CONTENTS

1. Introduction 9
1.1. Background 9
1.2. Rationale for conducting this study 10
2. Study Objectives and Endpoints 10
2.1. Primary objective 10
2.2. Secondary objective(s) 10
3. Study Design and Procedures 10
3.1. Overall study design and flow chart 10
3.2. Rationale for study design 12
3.3. Study visits 13
4. Study Population 13
4.1. Inclusion criteria 13
4.2. Exclusion criteria 13
4.3. Subject enrolment and randomisation 13
4.4. Discontinuation and withdrawal of subjects 14
4.4.1. Premature termination of the study 14
4.5. Re-screening 14
5. Study Treatments 14
5.1. Identity of investigational implants 14
5.2. Blinding 14
5.3. Randomisation 14
5.4. Concomitant medication 15
6. Study Measurements and Variables 15
6.1. Primary variable 15
6.2. Secondary variable(s) 15
7. Statistics 16
7.1. Sample size calculation 16
7.2. Statistical analysis 17
8. Data Management 18
8.1. Recording of data 18
8.2. Data storage and management 18
9. Quality Control and Quality Assurance 19
9.1. Audits and inspections 19
10. Ethics 19
10.1. Ethics committee 19
10.2. Informed consent 19
10.3. Subject data protection 20
10.4. Insurances 20
11. Protocol Deviations and Amendments 20
12. Report and publications 21
13. Study Timetable 21
13.1. Study period 21
13.2. Definition of “End of study” 21
14. Amendments to the study protocol 21

14.1. Re: 3.1: Capsular repair after posterior surgical approach 21
14.2. Re: 3.1: Fixation of components 21
15. List of References 24
Appendix 26
1. ICD codes for primary and secondary outcomes. 26

LIST OF ABBREVIATIONS

Abbreviation	Explanation
AO	"Arbeitsgemeinschaft Osteosynthese"
ASA	American Society of Anaesthesiologists
BMI	Body Mass Index
ICD	International Classification of Diseases
NPR	National Patient Register
RCT	Randomized Controlled Trial
SFR	Swedish Fracture Register
SHAR	Swedish Hip Arthroplasty Register
THA	Total Hip Arthroplasty

1. INTRODUCTION

1.1. Background

In Sweden, ***hip fractures*** annually affect close to 20,000 elderly, often frail patients and although the incidence of this injury seems to be stabilizing or even slightly declining, hip fractures cause an annual economic burden of no less than 800 million € in Sweden alone[1]. More than 2,000 Swedish patients with a displaced femoral neck fracture are annually treated with a THA[2], and the use of this strategy is increasing since it gives superior results when compared to hemiarthroplasty[3]. However, ***dislocation*** of the prosthetic head from the acetabular cup can occur, and this is an extremely painful event that leads to long-lasting impairment of the quality of life[4]. Even adherence to precautions aimed at preventing dislocations causes fear, anxiety and reduced quality of life in THA patients[5]. Very frequently, re-operations are needed in order to address the underlying cause of instability. According to recent Swedish registry findings, dislocation occurs in 8% of patients who receive a THA due to femoral neck fracture within 6 months after surgery[6], making this the most common complication after THA procedures in hip fracture patients. Male sex and a posterior surgical approach are two independent risk factors that each confer an approximately 1.3-fold increase in the risk of dislocation.

Dual mobility cups were developed in order to address the issue of joint instability after THA. In a traditional THA, a metal or ceramic femoral head with a diameter of typically 22- to 32-mm articulates with a polyethylene socket that is fixed to the acetabulum (Fig. 1). In the dual mobility cup, a spherical polyethylene liner encloses the metal femoral prosthesis head of 22- or 28-mm diameter, and this liner is moveable within the external metal shell that is fixed to the acetabulum. Small- or medium-sized cohort studies and registry analyses of larger cohorts indicate that the use of dual mobility cups in primary THA is associated with a reduction in the risk of dislocation in patients with osteoarthritis, but fewer studies address the results after the use of these devices in patients with femoral neck fractures. A Swedish registry study indicated that the use of dual mobility cups in an unselected cohort of THA patients was associated with a reduced risk of revision due to dislocation (relative risk = 0.4) when compared with standard cups[7], and a prospective single-cohort study of patients with a femoral neck fracture who were treated with dual mobility cups reported a very low dislocation risk of 1.4% within nine months[8]. Comparative cohort studies on patients with femoral neck fractures found that dual mobility cups conferred relative risks of dislocation of 0.3 or less when compared with standard cups or hemiarthroplasties[9,10]. A recent study on the hitherto largest sample of roughly 9,000 hip fracture patients estimated a relative risk of 0.45 for revision due to dislocation when comparing patients operated with dual mobility cups to those operated with standard cups[11]. A systematic review estimated a rate of dislocation of 2.5% within 1.3 years when dual mobility cups were used for hip fracture patients[12], however, a recent Danish study with a mean follow-up time of 5.4 years reported a dislocation rate of 4.7% in femoral neck fracture patients treated with dual mobility cups[13].

A serious limitation to all registry research on dislocations after THA is the failure to detect dislocations treated by closed reduction, since most registry analyses completely fail to take this endpoint into account[14]. Moreover, it is likely that most findings derived on registry-based or smaller, non-randomized comparative studies are confounded by indication, whereby dual mobility cups may be preferentially used in patients who are at higher risk of dislocation.

The question whether the use of dual mobility cups confers an increased risk of aseptic loosening or other implant-related complications has been investigated in some of the cited studies, and the

occurrence of polyethylene wear[15] and periprosthetic joint infections may be slightly increased[2], but this also has been contested[11].
High-level evidence on the use of dual mobility cups in hip fracture patients is limited to one pilot randomized study of 20 patients, concluding that a sufficiently powered RCT was not feasible within a UK setting[16].

1.2. Rationale for conducting this study

Observational, low-quality evidence that is equivalent with phases 1 through 2b proposed by the IDEAL framework indicates efficacy of the dual mobility cup in terms of preventing dislocations in hip fracture patients treated with a THA[17]. However, study findings are contradictory: Low dislocation rates of 1.5 to 2.5% within the first year are reported, but there is contrary evidence of higher dislocation rates of almost 5% in a longer perspective. Some long-term registry data that are comparable with phase 4 in the IDEAL framework are available, but the assessment stage (phase 3) with a sufficiently powered RCT has been omitted. Thus, there is insufficient evidence to support the efficacy of dual mobility cups, and reports on a higher risk of liner wear and periprosthetic joint infections in patients operated with these implants question its safety.
Therefore, an ***RCT on the use of dual mobility cups in patients with femoral neck fractures*** is urgently needed, and such a study was recommended both in the revised UK NICE guidelines and in an editorial in a leading orthopaedic journal[18].

2. STUDY OBJECTIVES AND ENDPOINTS

2.1. Primary objective

1) The primary objective is to assess whether dual mobility cups reduce the risk of dislocations in patients with a femoral neck fracture treated with a THA by 50% within one year, as suggested by observational data.

2.2. Secondary objective(s)

The secondary objective(s) of this study are to evaluate whether:

1) there is an increased risk of other adverse events such as re-operations (for any reason), periprosthetic joint infections, and mortality, after the use of dual mobility cups within one year and within three years.
2) patient-reported outcomes are improved after one year by the use of dual mobility cups.
3) the use of dual mobility cups is cost-effective.

3. STUDY DESIGN AND PROCEDURES

3.1. Overall study design and flow chart

The proposed study is designed as a ***register-nested RCT***. The study will be ***pragmatic***, with broad eligibility criteria, participant inclusion by other physicians than the operating surgeon ("third party inclusion"), and great freedom for surgeons to choose between different implant brands, surgical approaches, and post-operative regimes.

Two thirds of Swedish hip fracture patients receiving a THA are operated on via the direct lateral ***approach*** according to Hardinge or Gammer, and the remaining third is operated by a posterior approach.
It is expected that both the use of smaller head sizes and the use of posterolateral approaches will increase the risk of dislocation, but—importantly—there is no reason to believe that these potential confounders will be unequally distributed over the two study arms. However, post-hoc analyses stratified by cup brand, head size and surgical approach will be performed (see *"Statistical methods"*) in order to address this issue.

Standardization of surgery and post-operative treatment will be required of participating units in order to minimize the risk of confounding:

- Surgical approach can vary by surgeon, but individual surgeons must maintain the same approach for both study groups.
- If a posterior approach is used, a capsular and *m. piriformis* repair should be performed and documented.
- If a posterior approach is used, a capsular and m. piriformis repair should generally be performed and documented. If a participating unit does not routinely perform a posterior repair the unit can participate in the trial, provided that the absence of a posterior repair is equally applied to both intervention and control groups. A sensitivity analysis will be performed after trial conclusion with the aim of investigating whether such units have a higher than average incidence of the primary outcome.
- Component fixation can be cemented or uncemented dependent on local routines. If a participating unit routinely uses uncemented cups or stems for hip fracture patients the unit can participate in the trial, provided that the use of uncemented fixation of either component is equally applied to both intervention and control groups. A sensitivity analysis will be performed after trial conclusion with the aim of investigating whether such units have a higher than average incidence of the primary outcome or the secondary outcome 'any re-operation'.
- Weight bearing will be allowed without restriction post-operatively in both study groups, which today is standard practice.
- Post-operative mobilization will begin on day 0 or 1, which today is standard practice.
- Education and information around hip precautions must be consistent across groups, ensured by instructions in the study protocol stating that the same educational material and oral information is presented to all study participants within a given unit.
- Though specifics may vary between sites, each site will be required to provide the same rehabilitation across groups, ensured by instructions in the study protocol requiring each unit to use the same rehabilitation protocol for all study participants.

Figure 1 ***Study design: Register-based, randomised controlled trial***

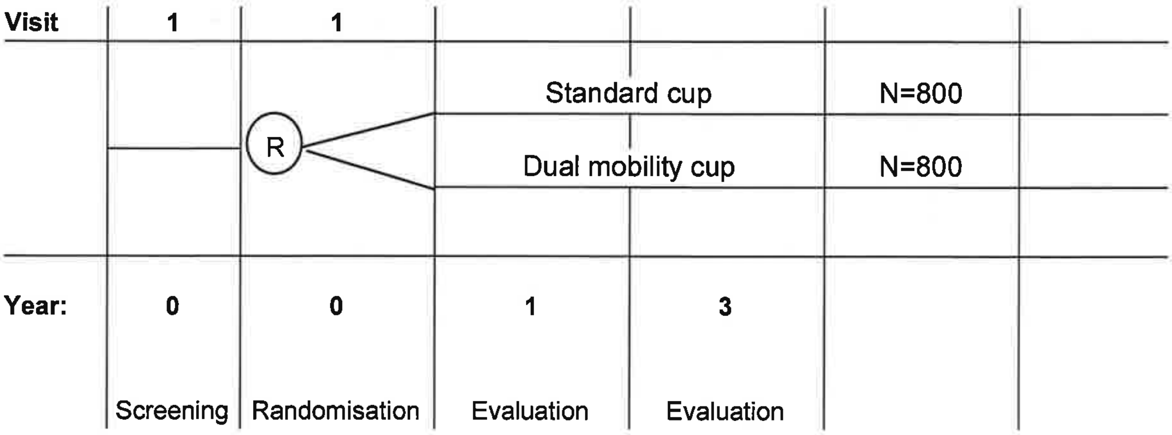

3.2. Rationale for study design

The rRCT study design enables us to perform a national multicenter randomised controlled trial without any additional follow-up. In this elderly population, the main advantage is the possibility to recruit a large sample size without burden the patients with additional follow-up visits. The Swedish personal identity number (PIN) allows the investigator to cross-check registers on an individual level. Data on fracture classification, age, sex, type of trauma, time of diagnosis with radiography, time of surgical treatment will be collected in the SFR and the randomisation will be done within the SFR registry-platform after informed consent has been obtained from the patient. Further variables will be registered by cross-checking with the Swedish Hip Arthroplasty Register with data on type of arthroplasty used (hemi or total hip), fixation method, manufacturer and type of components and any revision surgery performed. Data on reoperations are registered in the National Patient Register (NPR). Mortality is automatically cross-checked with the Swedish Cause of Death Register.

3.3. Study visits

There will be no formal clinical follow-up visits in addition to the local clinical routines. Data on reoperations and mortality are registered in the National Patient Register (NPR).

Day:	Visit 1 Screening and randomisation 0	Follow-up 1y	Follow-up End of Trial 3y
Informed consent	X		
Demography	X		
Inclusion/exclusion criteria	X		
Randomisation	X		
Outcome		X	X

4. STUDY POPULATION

4.1. Inclusion criteria

- Written informed consent
- Age ≥65 years
- Diagnosis: displaced femoral neck fracture type AO 31-B2 or B3/Garden type 3 or 4
- Eligible for THA according to local guidelines and routines

4.2. Exclusion criteria

- Previous inclusion of contralateral hip
- Delayed fracture surgery (date of injury >7 days prior to date of randomization)
- Pathological or stress fracture of the femoral neck, or fracture adjacent to a previous ipsilateral hip implant
- Inability or unwillingness to give written consent
- Dementia (as diagnosed by the screening physician)
- Unavailability of both interventions for a study subject (e.g., implants being out of stock, or lack of the individual surgeon's expertise to perform either procedure)

4.3. Subject enrolment and randomisation

Subject eligibility will be established before treatment randomisation. Subjects will be randomised strictly sequentially, as subjects are eligible for randomisation. If a subject discontinues from the study, the subject number will not be reused, and the subject will not be allowed to re-enter the study.

4.4. Discontinuation and withdrawal of subjects

Subjects are free to discontinue their participation in the study at any time without prejudice to further treatment. Patients will be withdrawn from study if the patient withdraws consent. Already collected study data for these patients will be kept in the study database, however new data, including data from registries will not be added. Patients prematurely withdrawn from the study will not be replaced.

4.4.1. Premature termination of the study

The study group may decide to stop the trial or part of the trial at any time. Furthermore, the investigator should promptly inform the Ethics Committee and provide a detailed written explanation.

4.5. Re-screening

Re-screening is not allowed.

5. STUDY TREATMENTS

5.1. Identity of investigational implants

The choice of supplier, brand and type of implants are based on the preference of each participating center.
Implant variation in Sweden is very limited. Three ***cup brands*** Avantage® (Zimmer), Polar® (Smith&Nephew), and Ades® (Zimmer) account for 97% of the dual mobility cups used in Swedish hip fracture patients, and none other than these are currently used at the participating units[2]. The variation among standard cups is slightly larger, with the Lubinus® (Waldemar Link), Marathon® (DePuy Synthes), Exeter RimFit® (Stryker), and Lubinus IP® (Waldemar Link) cups being used in about 95% of hip fracture patients. The participating units will use the implants that are routinely available for their hip fracture patients.
Femoral head sizes will be chosen according to local routines, which—for technical reasons—implies use of a 28-mm head when combined with the majority of dual mobility cups, and use of a 32-mm head when combined with most standard cups. In cases where patient anatomy demands for the use of cup sizes below 50 mm the use of 22-mm femoral heads may be required when combined with small dual mobility cups, and the use of 28-mm femoral heads may be needed when combined with small standard cups.
The ***stem component*** of each study THA will be the stem type that represents the local standard in the treatment of hip fracture patients. The Lubinus SP2® (Waldemar Link), the Exeter®(Stryker), and the MS-30® (Zimmer) stems are used for more than 90% of Swedish hip fracture patients receiving a THA, and all participating units have access to at least one of these brands, but the choice of stem is ultimately up to the surgeon.

5.2. Blinding

There will be no blinding.

5.3. Randomisation

This study is designed as a register-based RCT based on the platform of the Swedish Fracture Register. The subjects are randomised in a 1:1 ratio to receive standard (controls) or dual mobility

cups, using permuted block randomization. Randomisation will be performed through the web-based platform of the Swedish Fracture Register.

5.4. Concomitant medication

Patient will receive their ordinary medications and the standard pre- and postoperative treatment at each participating center.

6. STUDY MEASUREMENTS AND VARIABLES

6.1. Primary variable

Closed or open reduction of the index joint within one year
Dislocation will be ascertained by identifying the occurrence of any closed or open reduction of the previously inserted THA (the "index joint") within one year after surgery. Dislocation will be treated as a binary categoric variable, registered together with an underlying time to event-variable. The rationale for choosing the first year after surgery as the primary observation period is based on the finding that around 90% of revisions due to dislocation after primary THA are performed within this time frame[7]. The occurrence of dislocations is assessed by cross-matching study participants with the well-validated Swedish National Patient Register (NPR) where information on closed and open reductions including laterality is available. Closed and open reductions will be identified by the registration of any of the International Classification of Diseases (ICD; version 10) codes or NOMESCO codes indicative of this complication (see Appendix 1).
Presence of a contralateral THA is expected in about 20% of the study participants[2]. In order to avoid false-positive events due to errors in laterality coding, medical charts of all study participants who are detected as having experienced dislocations in the cross-matching procedure will be accessed, and it will be ascertained on which joint the reduction procedure or the revision surgery was performed (i.e., the index or contralateral joint).
The relative hazard of dislocation in the intervention compared to the control group will be assessed by fitting adjusted Cox proportional hazards models, and a risk reduction of at least 45% will be considered clinically relevant.

6.2. Secondary variable(s)

The relative hazard of the adverse events any re-operation of the index joint, periprosthetic joint infection and mortality within one year and within three years in the intervention compared to the control group will be assessed by fitting adjusted Cox proportional hazards models (see *"Statistical methods"*), and a risk increase of >20% within one year will be considered clinically relevant.

Any re-operation of the index THA

Re-operation will be treated as a binary categoric variable, recorded together with an underlying time to event-variable, and will be defined as the occurrence of any surgical procedure performed on the previously treated hip within one year after surgery. The occurrence of re-operations is assessed by cross-matching study participants with the NPR as described above, and will be defined by registration of at least one of the specified ICD or NOMESCO codes (Appendix 1).

Periprosthetic joint infection

Periprosthetic joint infection will be treated as a binary categorical variable, registered together with an underlying time to event-variable. It will be defined as the occurrence of any sign of deep

infection around the previously inserted THA within one year after surgery, defined by registration of ICD or NOMESCO codes (Appendix 1).

90-day and 1-year mortality

Occurrence of death (treated as a binary categoric variable), together with date and causes of death, are registered in the NPR, and 90-day and 1-year mortality will be obtained by cross-matching all study participants with the NPR.

Patient-reported outcomes

Patient-reported outcome will be assessed by use of EQ-5D domain scores and by the EQ-5D-VAS on a 0-100 numeric scale, where 0 represents the worst and 100 represents the best possible health state. Both parameters are routinely collected by the Swedish Hip Arthroplasty Register (SHAR) and will be assessed one year after the index procedure. EQ-5D domain scores and the EQ-5D-VAS will be analysed by proportional odds logistic regression (see *"Statistical methods"*). Any difference between the intervention compared with the control group that exceeds the minimal clinically-important difference of this instrument will be considered clinically relevant.

Cost-effectiveness

Procedural costs for intervention and control treatment will be recorded at all sites, ensuring documentation of baseline costs for the two treatment alternatives. Procedural costs of admissions for closed reductions as well as for reoperations will also be collected from all units. This allows for basic health economic calculations using Markov modelling.

7. STATISTICS

7.1. Sample size calculation

Main scenario

The null hypothesis is that the use of dual mobility cups does not reduce the risk of dislocation. We assume that the 1-year incidence of dislocations after insertion of conventional THA in patients with femoral neck fracture is 7%. This estimate is lower than the 8% dislocation rate described in Swedish hip fracture patients treated with a THA[6]. For the group operated on with a dual mobility cup, we assume a relative risk of 0.5, giving an incidence of dislocations of 3.6% in the intervention group. This risk reduction is based on the previously described relative risk of dislocations after the use of dual mobility cups that ranged from 0.3 to 0.5.

Alternative scenario

A recent study from Denmark on patients treated with a dual mobility cup due to a femoral neck fracture reported a dislocation rate of 4.7% after a mean follow-up of 5.4 years[13]. This higher dislocation rate may be due to the following factors: 1) The follow-up period in the cited study extends beyond the 1-year follow-up planned in our trial. 2) In line with Danish practice, all patients were operated via the posterolateral approach, which is associated with a 30% increase in the risk of dislocation[7]. 3) More than half of the Danish cohort was operated using cementless implants, a mode of fixation that is also associated with an increased risk of dislocation. The two latter factors would also increase the background dislocation rate in a control cohort. Thus, our alternative power calculation assumes a 1-year dislocation rate of 8% in the control group and 4.5% in the intervention group (relative risk of 0.55).

Sample size simulation

Sample size was determined by simulations under a simplified assumption of a constant risk during one year of follow-up, with 1/4 of control arm patients having an event risk of 6.4% (no risk factors),

1/2 patients with a 7.4% risk (one risk factor), and 1/4 patients with a 8.5% risk (two risk factors), corresponding to sex and surgical approach as independent risk factors associated with an increased risk of dislocation. Random censoring due to death was assumed to occur exponentially at 10%/year. This mortality figure is based on a study on Swedish hip fracture patients treated with a THA[19] and is also in accordance with mortality data annually published by the SHAR[2]. It has to be kept in mind that hip fracture patients treated with a THA are selected for this procedure because they are deemed fitter in terms of evident comorbidities than other hip fracture patients. With a sample size of ***n=1,600 patients***, the trial has:

- ***88% power*** to detect a reduction in 1-year dislocation rates ***from 7 to 3.6%, equalling a hazard ratio of 0.5 (main scenario),***
- and ***83% power*** to detect a reduction ***from 8 to 4.5%, equalling a hazard ratio of 0.55 (alternative scenario).***

Only selected units having performed no less than 20 dual mobility cup insertions in hip fracture patients can participate in the trial to ensure that the surgical expertise required to perform both intervention and control treatment is present at each unit. All 14 hitherto recruited units together performed about 2,200 THA procedures on hip fractures during the period 2016-2018[2], and more units are expected to be enrolled in the near future. The proposed sample size is thus feasible.

7.2. Statistical analysis

Analyses will be performed using the intention-to-treat principle including all randomized patients according to randomized treatment. Our primary outcome measure is the adjusted risk of dislocation treated by open or closed reduction within 1 year. Time to event will be described using Kaplan-Meier curves per randomized treatment group. The relative hazard of dislocation in the treatment compared to the control group will be estimated by Cox regression adjusted for sex and surgical approach (direct lateral/other) and presented as hazard ratio with 95% profile likelihood confidence interval and two-sided likelihood ratio p-value.

With the registry-nested follow-up, we assume that follow-up will be complete. In the rare case that a patient has incomplete follow-up, (s)he will be considered censored at last known follow-up. Death before dislocation will be handled as censoring at day of death.

The secondary outcome measures any re-operation, periprosthetic joint infection and mortality will be analysed and described in the same way as for the primary outcome.

Supplementary sensitivity analyses will be performed for all event endpoints. These analyses will primarily use logistic regression with the same covariates as the primary analysis, and as a supplement risk differences with Wald confidence intervals will be computed. To investigate sensitivity to baseline covariates, unadjusted Cox regression will be performed. Sensitivity analyses to investigate the impact of censoring by death, in addition to analysing death as an outcome, will include analyses of the composite of dislocation and death in the same way as for the primary outcome. Estimation of the risk of dislocation after one year will be investigated in an additional sensitivity analysis including all patients with a follow-up exceeding one year. Randomized and actual treatments will be described in a CONSORT diagram, and additional per-protocol analyses will be undertaken as sensitivity analyses. The threshold of statistical significance will be set at a two-sided p-value of 0.05. Secondary outcomes will be presented without formal multiplicity adjustment. A detailed statistical analysis plan will be completed before data base lock.

EQ-5D domain scores at one year will be summarized using descriptive frequency tables by randomized treatment. They will be analysed using proportional odds logistic regression adjusted for the baseline domain score as a categorical variable, and presented as the common odds ratio for all cut-points. For the primary presentation and analysis, missing domain scores due to death will be considered a separate category. For the adjusted analysis, missing baseline scores will be imputed using multiple imputation. Sensitivity analyses using observed cases only will also be provided.
EQ-5D VAS score at one year will be presented using tables of medians and quartiles as well as empirical cumulative distribution plots of VAS score and linear change in VAS from baseline. The VAS score will be analysed using proportional odds logistic regression adjusted for baseline score as a numerical variable modelled as a restricted cubic spline. Missing baseline scores will be imputed using multiple imputation. Outcome scores missing due to death will primarily be imputed as zero, with no imputation of other missing scores.
For all event outcome variables pre-defined subgroup/interaction analyses to assess the homogeneity of the treatment contrast will be performed, for sex, age, ASA class I/II/III[-IV], and BMI, and for the procedural characteristics femoral neck length, cup diameter, type of cup, type of stem, type of cement, and surgical approach. For categorical subgroup indicators, events will be described in each subgroup as for the entire population, and the treatment contrast in each subgroup will be estimated using a Cox proportional hazard model with treatment, subgroup indicator, and interaction, and presented with nominal 95% CI for each subgroup and the interaction p-value. For age and BMI, the interaction model will use restricted cubic spline modelling, and present the result as a curve of treatment contrast by covariate with 95% pointwise confidence bands, and the interaction p-value. Treatment comparisons are not relevant for subgroups that are specific to a single treatment arm. For such subgroups descriptive statistics including Kaplan-Meier plots will be presented for each subgroup.
For health economic studies, Markov modelling based on the assumption of clearly defined health states will be performed, and the primary outcome will be cost per quality-adjusted life year. Deterministic and probabilistic sensitivity analyses of the main model hypotheses and variables will be performed in addition to the main analyses.

8. DATA MANAGEMENT

8.1. Recording of data

All study data will be transferred from SFR, SHAR and the NPR into the study database, with the exception of the screening question answers that will be entered into the study database from the SFR interface. Data relevant to assess known confounders and primary and secondary outcomes will be collected retrospectively from the registries mentioned above.
The written informed consent will be stored at the study site. The investigator ensures that all source documents are accessible for monitoring.

8.2. Data storage and management

All data are recorded, handled and stored in a way that allows its accurate reporting, interpretation and verification. All source data including informed consent at each participating study center, a copy of the completed study database, original protocol with amendments and the final report will

be stored at the orthopaedic department at Uppsala University Hospital for a minimum period of 25 years after termination of the trial, according to the EU regulation 536/2014.

At the conclusion of the study, the occurrence of any protocol deviations will be determined. Data from the SFR on all study participants will be fused with all data on the aforementioned study participants available in the SHAR one year after the inclusion of the last patient. This combined dataset will then be sent to the NPR to obtain all registered ICD and NOMESCO codes for all study participants from the date of inclusion and onward. After these actions have been completed and the database has been declared to be complete and accurate, it will be locked and available for data analysis.

9. QUALITY CONTROL AND QUALITY ASSURANCE

The coordinator will have regular contacts with the clinic to verify presence of informed consents of participating subjects, to confirm that facilities remain acceptable, that the investigational team is adhering to the protocol, to verify inclusion/exclusion criteria, study main endpoints. The investigator should ensure that all persons assisting with the trial are adequately informed and trained about the protocol, that the standardization defined in section 3.1 is adhered to.

9.1. Audits and inspections

Authorized representatives of the study group, or an Ethics Committee may perform audits or inspection at the center. The investigator must ensure that all study documents are accessible for auditing and inspection. The purpose of an audit or inspection is to systematically and independently examine all study-related activities and documents, to determine whether these activities were conducted, and data were recorded, analyzed and accurately reported according to the protocol, and any applicable regulatory requirements.

10. ETHICS

The study is performed in accordance with the protocol, with the latest version of the Declaration of Helsinki, and applicable regulatory requirements. The regional ethical committee at Uppsala University has approved the study (Approval No. 2019-01137, date of issue: 2019-04-24).

10.1. Ethics committee

The final study protocol, including the final version of the Informed Consent Form and other information given to subjects e.g. advertisements, must be approved or given a favorable opinion in writing by an Ethics Committee (EC) as appropriate. The Principal Investigator is responsible for informing the EC of any amendment to the protocol, in accordance with local requirements.

10.2. Informed consent

The principal Investigator at each center will ensure that the subject is given written information about the nature, purpose and possible risks and benefits of the study. Subjects must also be notified that they are free to discontinue from the study at any time. The subject should be given the opportunity to ask questions and allowed time to consider the information provided.

The subject's signed and dated informed consent must be obtained before conducting any procedure specifically for the study. The monitor(s) will be granted direct access to the subject's original medical records for verification of clinical trial procedures and/or data, without violating the confidentiality of the subject, to the extent permitted by the applicable laws and regulations and that, by signing a written informed consent form, the subject' or the subject's legally acceptable representative is authorizing such access.

The original, signed Informed Consent Form (ICF) must be stored at the study site. A copy of the signed ICF must be given to the subject.

If a protocol amendment requires a change to the ICF, the EC must approve modifications that lead to a revised ICF before the revised form is used.

10.3. Subject data protection

The Informed Consent Form will incorporate wording that complies with relevant data protection and privacy legislation. Pursuant to this wording, subjects will be informed about the collection and by those persons who need that information for the purposes of the study.
The Informed Consent Form will explain that study data will be stored in a computer database, maintaining confidentiality in accordance with national data legislation. All data computer processed by the study group will be identified by ten-digit personal registration numbers.
The Informed Consent Form will also explain that for data verification purposes, authorized representatives of the study group, a regulatory authority or an Ethics Committee may require direct access to parts of the hospital or practice records relevant to the study, including subjects' medical history.

10.4. Insurances

The study subjects are covered by the Swedish Patient Injury Act.

11. PROTOCOL DEVIATIONS AND AMENDMENTS

Modifications to the signed protocol are only possible through approved protocol amendments and with the agreement of all responsible persons. Details of non-substantial amendments are to be clearly noted in the amended protocol.
A change that concerns; a new trial site, new principal investigator and or a new informed consent form should only be submitted to the concerned Ethics Committee.
In case of a substantial protocol amendment (e.g. change of; main purpose of the trial, primary/secondary variable, measurement of primary variable), the concerned Ethics Committee must be informed and should be asked for its opinion/approval prior implementation of amended protocol, as to whether a full re-evaluation of the ethical aspects of the study is necessary by the committee. This should be fully documented.

The Investigator must not implement any deviation from, or change to the protocol, without discussion with, and agreement by the study group and prior review and documented approval/favorable opinion of the amendment from the relevant ethics committee, except where it

is necessary to eliminate an immediate hazard to study subjects, or where the change(s) involves only logistical or administrative aspects of the study (e.g. change of telephone numbers).

12. REPORT AND PUBLICATIONS

After completion of the study, the results will be analyzed and a clinical study report will be prepared. Within one year after the end of the study, the study group will submit a final study report with the results of the study, including any publications/abstracts of the study, to the accredited Ethics Committee. In addition, upon study completion and finalization of the study report the results of this trial will be either submitted for publication and/or posted in a publicly accessible database of clinical trial results.

13. STUDY TIMETABLE

13.1. Study period

Estimated subject enrollment start: 2020-01-01
Subject enrollment stop: 2022-12-31
Subject last follow-up: 2023-12-31

13.2. Definition of "End of study"

The study group will notify the concerned Ethics Committee of the end of the study within a period of 90 days. *End of study is defined as one year after inclusion of the last subject.*

14. AMENDMENTS TO THE STUDY PROTOCOL

14.1. Re: 3.1: Capsular repair after posterior surgical approach

Date of amendment: 2020-02-07

> The original version under section 3.1 was: *"If a posterior approach is used, a capsular and m. piriformis repair should be performed and documented."*
> The novel version reads: ***"If a posterior approach is used, a capsular and m. piriformis repair should generally be performed and documented. If a participating unit does not routinely perform a posterior repair the unit can participate in the trial, provided that the absence of a posterior repair is equally applied to both intervention and control groups. A sensitivity analysis will be performed after trial conclusion with the aim of investigating whether such units have a higher than average incidence of the primary outcome."***

14.2. Re: 3.1: Fixation of components

Date of amendment: 2020-02-07

> The original version under section 3.1 was: *"Component fixation is required to be cemented which follows from the choice of cup and stem components given above. (Uncemented components are only very rarely used in Swedish hip fracture patients.)"*
> The novel version reads: ***"Component fixation can be cemented or uncemented dependent on local routines. If a participating unit routinely uses uncemented cups***

or stems for hip fracture patients the unit can participate in the trial, provided that the use of uncemented fixation of either component is equally applied to both intervention and control groups. A sensitivity analysis will be performed after trial conclusion with the aim of investigating whether such units have a higher than average incidence of the primary outcome or the secondary outcome 'any re-operation'."

14.3. Re: 3. Study design

Date of amendment: 2022-12-02

Subsequent to submission of UK study protocol "White 12/Duality" to RGEA in March 2022, REC submission in April 2022 and OCTRU approval in May 2022, the UK study arm opened for recruitment on 2022-12-02. According to the UK study protocol the UK study arm is handled by the White platform and is run under the acronym "White 12". See UK documentation for further details.

14.4. Re: 8.2 Data storage and management, and 10. Ethics

Date of amendment: 2023-11-07

Subsequent to establishment of a UK study arm (see above under amendment 14.3) an amendment regarding the original approval was submitted the Swedish Ethical Review Authority, the purpose of which was to seek approval for transfer of a study database containing all relevant information on the UK study arm to Sweden for building of a final study database. Furthermore, approval was requested to transfer the final study database from Sweden to the UK for analysis of specified secondary outcomes, including PROM data required to perform health economics analyses. This application was approved by the Swedish Ethical Review Authority (approval no. 2023-06139-02, date of approval 2023-11-07).

14.5. Re: Research body

Date of amendment: 2024-01-18

In the course of discussions with representatives for VG Region, the body responsible for the two registries underlying the duality-study, the Swedish Fracture Register and the Swedish Arthroplasty Register, on the process of data storage and handling of the research database it was decided that the research body in this current study protocol must be identical to the research body indicated in the application to the Swedish Ethical Review Authority. Consequently, from version 3.1 of this study protocol, the responsible research body for the study is forthwith designated as Uppsala University Hospital, not Uppsala University, which is now consistent with the application to the Swedish Ethical Review Authority. This change in research body is confirmed by the signature of the Head of Department of the Department of Orthopaedics and Hand Surgery, Uppsala University Hospital, Dr. Caroline Sköld.

14.6. Re: Informed consent

Date of amendment: 2024-12-04

We hereby change the method of documenting informed consent from research participants in the trial, where the inclusion period ended on April 1st, 2024. In accordance with the Swedish Ethical Review Act (2003:460), §§ 16 and 17, we have provided both oral and written information to the 1,378 included Swedish research participants regarding the overall plan for the research, its purpose, the methods used, any potential consequences and risks, as well as the fact that participation is voluntary and can be terminated at any time. The participants have thus received all the relevant information required to give their consent to the research, in accordance with legal requirements. This has been documented through the checking of a checkbox in the digital "screening window" at the time of the participant's inclusion, as well as in the form of a physical consent form.

We now determine that presence of the documented consent will be considered solely based on the filled checkbox in the randomization window, which the informing physician checked after obtaining informed consent. This checkbox, along with the completion of three additional screening questions, has been recorded in the so-called "screening log," which is stored by the Swedish Fracture Register together with the research database. All 1,378 included research participants, according to this screening log, have provided their informed consent to the including physician. Simultaneously, we establish that the previous requirement for a physically signed consent form, as described in the original application and study plan, will be waived. This procedural change has been approved by the Swedish Ethical Review Authority (Dnr 2024-06845-02, date of approval 2024-12-04).

15. LIST OF REFERENCES

1. Svedbom A, Hernlund E, Ivergard M, et al. Osteoporosis in the European Union: a compendium of country-specific reports. *Archives of osteoporosis* 2013; **8**: 137.
2. Swedish_Hip_Arthroplasty_Register. Annual Report, 2017.
3. Hopley C, Stengel D, Ekkernkamp A, Wich M. Primary total hip arthroplasty versus hemiarthroplasty for displaced intracapsular hip fractures in older patients: systematic review. *BMJ (Clinical research ed)* 2010; **340**: c2332.
4. Enocson A, Pettersson H, Ponzer S, Tornkvist H, Dalen N, Tidermark J. Quality of life after dislocation of hip arthroplasty: a prospective cohort study on 319 patients with femoral neck fractures with a one-year follow-up. *Quality of life research : an international journal of quality of life aspects of treatment, care and rehabilitation* 2009; **18**(9): 1177-84.
5. Lee GRH, Berstock JR, Whitehouse MR, Blom AW. Recall and patient perceptions of hip precautions 6 weeks after total hip arthroplasty. *Acta Orthop* 2017; **88**(5): 496-9.
6. Hansson S. Clinical results after hip fracture - with special focus on hip arthroplasty. Lund; 2018.
7. Hailer NP, Weiss RJ, Stark A, Karrholm J. The risk of revision due to dislocation after total hip arthroplasty depends on surgical approach, femoral head size, sex, and primary diagnosis. An analysis of 78,098 operations in the Swedish Hip Arthroplasty Register. *Acta Orthop* 2012; **83**(5): 442-8.
8. Adam P, Philippe R, Ehlinger M, et al. Dual mobility cups hip arthroplasty as a treatment for displaced fracture of the femoral neck in the elderly. A prospective, systematic, multicenter study with specific focus on postoperative dislocation. *Orthop Traumatol Surg Res* 2012; **98**(3): 296-300.
9. Tarasevicius S, Robertsson O, Dobozinskas P, Wingstrand H. A comparison of outcomes and dislocation rates using dual articulation cups and THA for intracapsular femoral neck fractures. *Hip international : the journal of clinical and experimental research on hip pathology and therapy* 2013; **23**(1): 22-6.
10. Bensen AS, Jakobsen T, Krarup N. Dual mobility cup reduces dislocation and re-operation when used to treat displaced femoral neck fractures. *Int Orthop* 2014; **38**(6): 1241-5.
11. Jobory A, Karrholm J, Overgaard S, et al. Reduced Revision Risk for Dual-Mobility Cup in Total Hip Replacement Due to Hip Fracture: A Matched-Pair Analysis of 9,040 Cases from the Nordic Arthroplasty Register Association (NARA). *J Bone Joint Surg Am* 2019; **101**(14): 1278-85.
12. Darrith B, Courtney PM, Della Valle CJ. Outcomes of dual mobility components in total hip arthroplasty: a systematic review of the literature. *The bone & joint journal* 2018; **100-b**(1): 11-9.
13. Tabori-Jensen S, Hansen TB, Stilling M. Low dislocation rate of Saturne((R))/Avantage((R)) dual-mobility THA after displaced femoral neck fracture: a cohort study of 966 hips with a minimum 1.6-year follow-up. *Archives of orthopaedic and traumatic surgery Archiv fur orthopadische und Unfall-Chirurgie* 2019; **139**(5): 605-12.
14. Devane PA, Wraighte PJ, Ong DC, Horne JG. Do joint registries report true rates of hip dislocation? *Clin Orthop Relat Res* 2012; **470**(11): 3003-6.
15. Tabori-Jensen S, Frolich C, Hansen TB, Bovling S, Homilius M, Stilling M. Higher UHMWPE wear-rate in cementless compared with cemented cups with the Saturne(R) Dual-

Mobility acetabular system. *Hip international : the journal of clinical and experimental research on hip pathology and therapy* 2018; **28**(2): 125-32.
16. Griffin XL, Parsons N, Achten J, Costa ML. A randomised feasibility study comparing total hip arthroplasty with and without dual mobility acetabular component in the treatment of displaced intracapsular fractures of the proximal femur : The Warwick Hip Trauma Evaluation Two : WHiTE Two. *The bone & joint journal* 2016; **98-b**(11): 1431-5.
17. Cook JA, McCulloch P, Blazeby JM, et al. IDEAL framework for surgical innovation 3: randomised controlled trials in the assessment stage and evaluations in the long term study stage. *BMJ (Clinical research ed)* 2013; **346**: f2820.
18. Horriat S, Haddad FS. Dual mobility in hip arthroplasty: What evidence do we need? *Bone & joint research* 2018; **7**(8): 508-10.
19. Hailer NP, Garland A, Rogmark C, Garellick G, Karrholm J. Early mortality and morbidity after total hip arthroplasty in patients with femoral neck fracture. *Acta Orthop* 2016; **87**(6): 560-6.

Appendix
1. ICD codes for primary and secondary outcomes.

Endpoint	ICD	NOMESCO
**Dislocation**	M24.3, M24.4, M24.4F, S73.0, T93.3,	NFH00, NFH02, NFH20, NFH21, NFH22
**Periprosthetic joint infection**	M00.0, M00.0F, M00.1, M00.2, M00.2F, M00.8, M00.8F, M00.9, M00.9F, M86.0F, M86.1F, M86.6, M86.6F, T81.4, T84.5, T84.5F, T84.5X, T84.7, T84.7F	NFSx, NFA12, TNF05, TNF10
**Any re-operation**		Any of the codes above, and: NFA00-22, NFA31-32, NFCx, NFF01-12, NFL09-19, NFL39-49, NFL69-99, NFM09-29, NFM49, NFM79-99, NFTx, NFWx
