# Supplementary material for: Dual mobility versus standard cups in total hip replacement for displaced femoral neck fractures (Duality): an international, multicentre, randomised, controlled, superiority trial
Source: Lancet. 2026 Jul 25;408(10552):348–56. doi: 10.1016/S0140-6736(26)00759-2 (PMC13425373; doi:10.1016/S0140-6736(26)00759-2)
Supplement: Supplementary appendix [file mmc3.pdf]

THE LANCET

Supplementary appendix 3

This appendix formed part of the original submission. We post it as supplied by the authors.

Supplement to: Hailer NP, Griffin XL, Mukka S, et al. Dual mobility versus standard cups in total hip replacement for displaced femoral neck fractures (Duality): an international, multicentre, randomised, controlled, superiority trial. *Lancet* 2026; published online July 2. https://doi.org/10.1016/S0140-6736(26)00759-2.

APPENDIX 12 to WHiTE Platform Master Protocol

World Hip Trauma Evaluation 12

Dual mobility versus standard articulation total hip replacement in the treatment of older adults with a hip fracture (DUALITY)

This appendix must be read with the accompanying WHiTE Platform Master Protocol. This appendix describes only the additional details relevant to the conduct of this particular randomised comparison within the context of the overarching master protocol.

This comparison is funded by the National Institute for Health Research (NIHR) Research for Patient Benefit programme (NIHR 203115). The views expressed are those of the author(s) and not necessarily those of the NIHR or the Department of Health and Social Care.

© Copyright: The University of Oxford and Oxford University Hospitals NHS Foundation Trust 2019

Appendix 12: World Hip Trauma Evaluation – DUALITY

Short title: WHiTE 12 - DUALITY

NIHR RfPB Ref: NIHR203115

ISRCTN: 11895196

Date and Version No: V3.0 08Nov2024

**Lead Investigator:**	Xavier L Griffin
	Queen Mary University of London
**Investigators:**	Michael Whitehouse, University of Bristol
	Sarah Lamb, University of Exeter
	Borislava Mihaylova, Queen Mary University of London
	Rory Middleton, Royal Cornwall Hospitals NHS Trust
	Duncan Appelbe, University of Oxford
	Juul Achten, University of Oxford
	Jonathan Cook, University of Oxford
	Richard Grant, Patient and Public Involvement Representative

**Funder:**	National Institute for Health Research (NIHR) Research for Patient Benefit (RfPB) Programme

We declare no conflicts of interest.

© Copyright: The University of Oxford and Oxford University Hospitals NHS Foundation Trust 2019

TABLE OF CONTENTS

1 APPENDIX AMENDMENT HISTORY 5

2 KEY CONTACTS 6

3 LAY SUMMARY 7

4 DUALITY SYNOPSIS 8

5 ABBREVIATIONS 11

6 BACKGROUND AND RATIONALE 11

6.1 What is the clinical problem being addressed? 11

6.2 How does the existing literature support this proposal? 12

6.3 Need for this comparison 12

7 OBJECTIVES AND OUTCOME MEASURES 12

7.1 Primary objective 12

7.2 Secondary objectives 13

7.3 Outcome Measures 13

7.3.1 Primary 13

7.3.2 Secondary 13

8 DESIGN 14

8.1 Concept 14

9 COMPARISON PROCEDURES 14

9.1 PARTICIPANT IDENTIFICATION 14

9.1.1 Comparison participants 14

9.1.2 Inclusion criteria 14

9.1.3 Exclusion criteria 15

9.2 Consent 15

9.3 Randomisation 15

9.4 Blinding 15

9.5 Description of the randomised treatments 15

9.5.1 Preoperative assessments 15

9.5.2 Anaesthetic technique 15

9.5.3 Surgical treatment 16

9.5.4 Early post-operative care 16

9.6 Assessments 16

9.6.1 Schedule of assessments 16

9.6.2 Visits and Contacts 17

9.7 Definition of End of Comparison 17

© Copyright: The University of Oxford and Oxford University Hospitals NHS Foundation Trust 2019

10 SAFETY REPORTING 17
10.1 Related and expected Serious Adverse Events 18
11 STATISTICS & ANALYSES 18
11.1 Sample size determination 18
11.2 Analysis populations 18
11.3 The level of statistical significance 19
11.4 Statistical Analysis 19
11.5 Health economic analysis 20
12 DATA PROCESSING AND DATA SHARING 21
13 DISSEMINATION POLICY 22
14 REFERENCES 23
ANNEX A: FLOW CHART 26
ANNEX B: Data linkage planned within UK 27
ANNEX C: Data sharing of pseudonymised trial source data between Universities of Oxford and Uppsala 28

© Copyright: The University of Oxford and Oxford University Hospitals NHS Foundation Trust 2019

1 APPENDIX AMENDMENT HISTORY

Amendment No.	Protocol Appendix Version No.	Date issued	Author(s) of changes	Details of Changes made
AM14<br>(SA 05)	2.0	16Dec2022	Elisa Basso<br>Amrita Athwal	Addition of Comparison Manager<br>Correction of reference to 'comparison' as opposed to 'trial' or 'study' throughout document where appropriate<br>4. and 7 Alignment of wording of objectives for consistency with master protocol and clarification of distinguishing comparison-specific time-points from those that fall under the Platform common outcome set. Addition of wording of mobility and residential status at 12 months as secondary objective.<br>9.1.3. Addition of reference to overarching exclusion criteria in WHiTE Platform master protocol<br>10. Clarification added regarding justification for no requirement to collect AEs or unrelated SAEs.
AM24 (SA10)	3.0	08Nov2024	Romina Basting, Kate Herbert, Amrita Athwal	Removal of recruitment window.<br>Staffing updates.<br>Section 8.1, align collection of comparison complications with clarification of complications list in the overarching WHiTE Platform Master Protocol

© Copyright: The University of Oxford and Oxford University Hospitals NHS Foundation Trust 2019

2 KEY CONTACTS

**Lead Investigator**	Professor Xavier Griffin<br>x.griffin@qmul.ac.uk<br><br>Barts Bone and Joint Health, Barts and The London School of Medicine and Dentistry, Queen Mary University of London, 4 Newark Street, London E1 4AT
**Comparison Manager**	Rhys Painton<br>white12-duality@ndorms.ox.ac.uk<br>Kadoorie Centre, NDORMS, University of Oxford, John Radcliffe Hospital, Headley Way, Oxford, OX3 9DU
**Funder(s)**	National Institute for Health Research, Research for Patient Benefit (RfPB) programme, Central Commissioning Facility, (CCFRMS), Grange House 15 Church Street, Twickenham, TW1 3NL, 020 8843 8000
**Senior Statistician**	Professor Jonathan Cook, jonathan.cook@ndorms.ox.ac.uk
**Committees**	**Comparison Management Group**<br>Xavier Griffin<br>Michael Whitehouse<br>Sarah Lamb<br>Borislava Mihaylova<br>Rory Middleton<br>Duncan Appelbe<br>Juul Achten<br>Jonathan Cook<br>Richard Grant<br>Amrita Athwal<br>Rhys Painton

© Copyright: The University of Oxford and Oxford University Hospitals NHS Foundation Trust 2019

3 LAY SUMMARY

Every year around 70,000 people in the UK break their hip. Hip fractures are a common and very serious injury in older people, similar in impact to a major stroke. We will investigate two treatments for one specific type of hip fracture. Currently, surgeons use one of two types of total hip replacement, which is an operation to replace both the ball and socket of the hip joint. One type uses a small ball within the socket, in the other the ball is enclosed within a larger plastic ball; we do not know which is the better treatment.

We will investigate which of these treatments gives a better result for people aged 65 years and over with a hip fracture from 30 hospitals across the UK. We want to look at whether people have had any complications after their surgery, particularly dislocation, where the ball comes out of the socket. We will ask people how they feel and how active they are a year after their fracture. We will also work out the cost of the two treatments – for the individual, for the health service and in terms of social support in the following year.

We have previously tried to answer this important question in a pilot study. We concluded that it wasn't possible to carry out the necessary study since it would need to be very large and expensive. Since then, there has been greatly increased use of the alternative type of hip replacement without any evidence. In this study we will work in collaboration with other international partners so that taken together we will be able to answer the question more quickly and at a cost that is reasonable.

To compare the two treatments properly we think we will need 1600 people to take part overall, we anticipate that 450 will come from the UK part of the study. If people agree to take part, they will be placed into one of two groups through a process called randomisation which makes sure that the groups are similar and the comparison is fair. After their operation everyone will have the usual ward care, rehabilitation and follow up that is standard practice at their hospital.

The researchers will ask participants about any complications from the surgery, their health, walking ability and other daily activities, as well as any additional costs. Answers will be collected at the time of the surgery, at 4 and 12 months after the injury; the results from the two groups will then be compared.

© Copyright: The University of Oxford and Oxford University Hospitals NHS Foundation Trust 2019

4 DUALITY SYNOPSIS

The aims of DUALITY are to investigate whether the risk of dislocation after total hip replacement (THR) surgery performed for femoral neck fracture is reduced after the use of dual mobility (DM) versus standard articulation THR.

DUALITY will be conducted in collaboration with a Swedish, pragmatic, superiority randomised clinical trial conducting the same investigation governed by a sister protocol. The Swedish trial is co-ordinated and sponsored by Uppsala University (independent of this UK trial), registered at clinicaltrials.gov NCT03909815 and funded by the Swedish Research Council (VR2019-00436).[1] A total of 1600 Swedish and UK participants will be randomly allocated on a 1:1 ratio to either DM or standard THR. It is our intention to combine data from both studies to be analysed together – the statistical analysis will be conducted by the Swedish team and the economic evaluation will be conducted by the UK team.

DUALITY will be embedded as a randomised comparison within the WHiTE Platform. This protocol appendix details the UK Platform-embedded trial.

© Copyright: The University of Oxford and Oxford University Hospitals NHS Foundation Trust 2019

**Comparison title**	World Hip Trauma Evaluation – Dual Mobility Cups in Hip Fracture Patients (DUALITY)				
**Short title**	WHiTE 12 - DUALITY				
**Registration**	The comparison has been registered with the current controlled trials database under reference number ISRCTN 11895196				
**Funder of DUALITY comparison**	Department of Health – NIHR Research for Patient Benefit (RfPB) Programme				
**Design**	Pragmatic, multicentre, two-arm randomised controlled superiority comparison with parallel economic analyses from a UK NHS perspective and direct patient follow-up to 12-months				
**Participants**	Patients with a displaced intracapsular fracture and meet the DUALITY eligibility criteria				
**Sample Size**	Anticipated to be approximately 450				
**Planned Comparison Duration**	3 months set-up – 12 months recruitment – 12 months follow-up– 3 months analysis & reporting				
**DUALITY Outcomes**	**DUALITY Objectives**	**Instruments**	**Time-points**		
			**Part of Platform common outcome set**	**DUALITY-specific**	
**Primary**	To compare the risk of dislocation in the first 12 months post-diagnosis of a hip fracture between the treatment groups.	Patient-completed complications via medical follow-up CRF confirmed with hospital records and routine datasets.	**Participant:** 4 months post-diagnosis of a hip fracture	**Participant:** 12 months post-diagnosis of a hip fracture **Medical record and routine data:** discharge and 12 months post-diagnosis of a hip fracture	
**Secondary**	1) To compare the risk of all cause re-operation in the first 12 months post-diagnosis of a hip fracture between the treatment groups.	Patient-completed complications via medical follow-up CRF confirmed with hospital records and routine datasets.	**Participant:** 4 months post-diagnosis of a hip fracture **Medical record:** hospital discharge	**Participant:** 12 months post-diagnosis of a hip fracture **Medical record and routine data:** 12 months post-diagnosis of a hip fracture	
	2) To compare mortality risk in the first 12 months post-diagnosis of a hip fracture between the treatment groups.	Death notification CRF.	**Medical record and routine data:** 4 months post-diagnosis of a hip fracture	**Medical record and routine data:** Mortality check at 12 months post-diagnosis of a hip fracture	
	3) To compare the risk of implant-related infections in the first 12 months post-diagnosis	Patient-completed complications via medical follow-up CRF confirmed	**Participant:**	**Participant:** 12 months post-	

© Copyright: The University of Oxford and Oxford University Hospitals NHS Foundation Trust 2019

	of a hip fracture between the treatment groups.	with hospital records and routine datasets.	4 months post-diagnosis of a hip fracture<br>**Medical record:** hospital discharge	diagnosis of a hip fracture<br>**Medical record and routine data:** 12 months post-diagnosis of a hip fracture.
	4) To compare Health Related Quality of Life (HRQoL) at 4 and 12 months post-diagnosis of a hip fracture between the treatment groups	EuroQol 5 Dimensions 5 levels (EQ-5D-5L).	**Participant:** Baseline and at 4 months post-diagnosis of a hip fracture	**Participant:** 12 months post-diagnosis of a hip fracture
	5) To compare mobility at 4 and 12 months post-diagnosis of a hip fracture between the treatment groups	modified New Mobility Score (mNMS)	**Participant:** Baseline and at 4 months post-diagnosis of a hip fracture	**Participant:** 12 months post-diagnosis of a hip fracture
	6) To compare residential status at 4 and 12 months post-diagnosis of a hip fracture between the treatment groups.	UK National Hip Fracture Database (NHFD) Residential Status questions	**Participant:** Baseline and at 4 months post-diagnosis of hip fracture	**Participant:** 12 months post-diagnosis of a hip fracture
	7) To compare the healthcare and broader resource implications up to 12 months post-diagnosis of a hip fracture between the treatment groups from an NHS and UK Social Care perspective.	Review of hospital medical notes complemented by patient-completed resource use questionnaire and routine datasets.	**Participant:** Baseline and at 4 months post-diagnosis of a hip fracture<br>**Medical record and routine data:** Hospital discharge	**Participant:** 12 months post-diagnosis of a hip fracture<br>**Medical record and routine data:** 12 months post-diagnosis of a hip fracture
**Treatment**	Dual Mobility Total Hip Replacement (the cup must be of dual articulation, non-constrained design)			
**Comparator**	Standard Total Hip Replacement (the cup and liner must be of a single articulation, non-constrained design)			

© Copyright: The University of Oxford and Oxford University Hospitals NHS Foundation Trust 2019

5 ABBREVIATIONS

AE	Adverse Event
BOA	British Orthopaedic Association
CRF	Case Report Form
DMP	Data Management Plan
DUALITY	Dual Mobility trial
EFORT	European Federation of National Associations of Orthopaedics and Traumatology
EQ-5D-5L	EuroQol 5 Dimension 5 Level
HES	Hospital Episode Statistics
HRG	Healthcare Resource Group
HRQoL	Health Related Quality of Life
ITT	Intention to Treat
mNMS	modified New Mobility Score
NHS	National Health Service
NICE	National Institute for Health and Care Excellence
NIHR	National Institute for Health and Care Research
NJR	National Joint Registry
OTA	Orthopaedic Trauma Association
OTS	Orthopaedic Trauma Society
QALY	Quality Adjusted Life Year
RCT	Randomised Clinical Trial
REC	Research Ethics Committee
SAE	Serious Adverse Event
THR	Total Hip Replacement
WHiTE	World Hip Trauma Evaluation

6 BACKGROUND AND RATIONALE

6.1 What is the clinical problem being addressed?

Hip fracture is one of the biggest challenges facing patients and healthcare systems. There are more than 70,000 hip fractures in the UK every year and 1.3 million worldwide,[2,3] projected to rise to more than 6 million by 2050.[4] The global cost of this clinical problem is estimated at 1.75 million disability adjusted life years lost and represents 1.4% of the total healthcare burden in established market economies.[4,5] People suffering hip fracture have a 30-day mortality of 7%, a 1-year mortality of 25% and experience a permanent reduction in their health related quality-of-life similar to that of a person with Parkinson's disease or multiple sclerosis.[6] Displaced intracapsular fractures, those broken at the level of the femoral neck and where the bone fragments have become malaligned, represent approximately half of all hip

© Copyright: The University of Oxford and Oxford University Hospitals NHS Foundation Trust 2019

fractures.[7] 95% of people with these hip fractures are treated with a hip replacement; those who are independently mobile, have few comorbidities, and are cognitively intact commonly receive a total hip replacement (THR)[7,8] where both the ball and the socket of the hip joint are replaced with metal and plastic implants.

6.2 How does the existing literature support this proposal?

Recent international cohort and registry observational studies have demonstrated widespread variation in clinical practice in terms of which type of implant is used for these patients.[9,10] The risk of dislocation after standard THR for hip fracture patients is estimated to be up to 13%.[9] A recent meta-analysis of 125 studies reported a pooled risk of dislocation of 2.1% at 6 years follow-up all types of THR; the risk ratio for Dual Mobility (DM) versus standard THR was 0.15 (95% CI 0.08-0.29).[11] International arthroplasty registries have reported similarly low rates of dislocation.[9] Despite the potential lower risk of dislocation with DM THR, there are concerns regarding the long term outcomes with DM THR with conflicting observational evidence around wear[12,13] and infection.[14] There has been a previous attempt to perform a randomised trial (RCT) to address this question, conducted before the widespread uptake of DM technology; the feasibility phase concluding that this question could not be answered in a UK only study due to the large sample needed and the consequently long and expensive recruitment required.[15] DUALITY is a RCT currently running in Sweden, designed to compare the clinical and cost effectiveness of standard with DM THR in older adults with a hip fracture (clinicaltrials.gov NCT03909815). By conducting a UK version of the DUALITY study in collaboration with Uppsala University, within the UK hip fracture trials platform (WHiTE PLATFORM), the existing infrastructure and multinational collaboration can be leveraged to overcome the feasibility barriers previously identified around this question;[16] delivering an NHS-applicable estimate of clinical effectiveness and cost-effectiveness from a NHS perspective.

6.3 Need for this comparison

The standard type of THR involves a single joint between a relatively small ball and the socket; with an associated risk of dislocation, where the ball disengages with the socket, of approximately 10%.[9] Dislocation requires readmission to hospital for the hip to be put back into joint. Most patients who experience a dislocation go on to have recurrent dislocations and one half of those undergo revision surgery with a high risk of further complications.[17,18] Dislocation and revision for dislocation is associated with poorer outcomes.[18] The mortality following second surgery for hip fractures approaches 50% in this frail group of patients; the functional outcome of revision hip replacement is less successful when compared with an index procedure.[19] The costs associated with second operations for hip fracture are enormous. Recent estimates from European centres participating in the multinational randomised hip fracture trial HEALTH[20] suggest that second operations cost almost €14,000 per patient and contribute to 5% of the overall hospital costs of delivering hip fracture care.[21] An alternative design, the dual mobility (DM) THR may be less likely to dislocate. Here, the ball and socket are still replaced but the small ball is encased in a much larger plastic ball; improving the stability of the hip replacement by increasing the range of movement of the joint prior to dislocation. DM THR may reduce the risk of dislocation whilst providing similar functional outcomes as a standard THR.[11] For this reason, DM THR may be a preferred primary treatment; but there are concerns that it has a shorter longevity[22] due to increased wear[23] and an increased risk of implant-related infection.[12] Coupled with this DM THR is typically one third more expensive again than standard THR. The aim of this comparison is to investigate the clinical and cost effectiveness of dual mobility versus standard articulation THR in the treatment of adults aged 65 years and over with an acute, displaced intracapsular hip fracture.

7 OBJECTIVES AND OUTCOME MEASURES

7.1 Primary objective

To compare the risk of dislocation in the first 12 months post-diagnosis of a hip fracture between the treatment groups.

© Copyright: The University of Oxford and Oxford University Hospitals NHS Foundation Trust 2019

7.2 Secondary objectives

1. To compare the risk of all cause re-operation within the first 12 months post-diagnosis of a hip fracture between the treatment groups.

2. To compare mortality risk in the first 12 months post-diagnosis of a hip fracture between the treatment groups.

3. To compare the risk of implant-related infections in the first 12 months post-diagnosis of a hip fracture between the treatment groups.

4. To compare health-related quality of life at 4* and 12 months post-diagnosis of a hip fracture between the treatment groups.

5. To compare mobility at 4* and 12 months post-diagnosis of a hip fracture between the treatment groups.

6. To compare residential status at 4* and 12 months post-diagnosis of a hip fracture between the treatment groups.

7. To compare the healthcare and broader resource implications up to 12 months post-diagnosis of a hip fracture between the treatment groups from an NHS and UK Social Care perspective.

*These time-points indicate that these objectives are already collected as part of the overarching platform.

7.3 Outcome Measures

The common outcome data described in the Master Protocol at baseline and 4 months post-diagnosis of a hip fracture will be collected and augmented with additional data collection at baseline and 12 months post-diagnosis of a hip fracture as per section 9.6.1.

7.3.1 Primary

The adjusted risk of dislocation treated by open or closed reduction within 12 months post-diagnosis of a hip fracture.
Dislocation events will be obtained through participant-reported complications via medical follow up CRFs and review of the participants' medical records.

7.3.2 Secondary

As part of the common outcome instruments, described in the master protocol, implant-related infection and re-operation events will be collected from participant-reported complications CRFs and review of the participants' medical records:

Reoperation: any surgical procedure performed on the index THR will be collected.

Implant-related infection: There is no consistent international consensus for the diagnosis of implant related deep infection. Here, this will be defined clinically by the treating surgical team, as a diagnosis recorded in the participants' medical records.

© Copyright: The University of Oxford and Oxford University Hospitals NHS Foundation Trust 2019

8 DESIGN

8.1 Concept

DUALITY will be embedded as a randomised comparison within the existing WHiTE Platform testing clinical superiority between the treatment groups in collaboration with Uppsala University Sweden, with a parallel economic analysis in the UK. Approximately 450 participants will be recruited in the UK. Both treatments are routinely used within the NHS. Clinical teams across the NHS are very familiar with both treatments. Recruitment in the UK will take place at approximately 30 recruitment centres over a period of twelve months.

Screening logs will be kept at each recruitment centre to determine the number of patients assessed for eligibility and reasons for any exclusion. The number of eligible and recruited patients, and the number of patients who decline consent or withdraw will be recorded.

Assessments will include all those described in the Master protocol, augmented with additional data relevant to this specific randomised comparison. In summary:

Baseline demographic data will be collected as per the requirements in the master protocol (see section 12.2 of the master protocol), with additional data relating to index surgery. When the patient is discharged from hospital, the local research team will check the participant medical records for any early complications.

Data being collected at 4 months post-diagnosis of a hip fracture will be that which satisfies the platform outcomes (see master protocol).These include data relating to dislocation, implant-related infections and all-cause re-operation will be collected as a part of this, which will inform the primary and secondary outcomes for DUALITY. The same data will be collected at 12 months post-diagnosis of a hip fracture.

As per section 12.3.5 of the master protocol any complications reported by participants via the medical follow-up forms will be queried by central study staff for confirmation by either the recruitment centre, or the participant's GP against their medical notes. This will be completed at the 12 months post-diagnosis of a hip fracture time-point.

9 COMPARISON PROCEDURES

A comparison flow chart is shown in Annex A.

9.1 PARTICIPANT IDENTIFICATION

9.1.1 Comparison participants

A subset of participants in the overarching WHiTE platform will be eligible for this randomised comparison.

9.1.2 Inclusion criteria

Adults aged 65 years and over with a displaced intracapsular hip fracture who meet the accepted criteria for consideration of a THR as per current NICE guidance:[11]

*N.B. whereas the over-arching WHiTE Platform inclusion criteria allows adults aged 60 years and over, this age requirement is specific to DUALITY.

© Copyright: The University of Oxford and Oxford University Hospitals NHS Foundation Trust 2019

9.1.3 Exclusion criteria

In addition to the exclusion criteria stated in the overarching master protocol, the participant is not eligible if ANY of the following apply:

- Delayed fracture surgery (date of presentation to recruitment centre more than seven days prior to date of randomisation).
- Pathological or stress fracture of the femoral neck.
- Fracture adjacent to a previous ipsilateral hip implant, such as a previously inserted screw or plate.

9.2 Consent

Patients will be presumed to have capacity unless established otherwise and the default will be to seek prospective individual consent from every patient. Where patients do not have capacity, those procedures laid down in Section 11.4 of the master protocol will apply.

With regards to these provisions, the randomised comparison described in this appendix is **not** a clinical trial of an investigational medicinal product.

9.3 Randomisation

Randomisation will be as per section 11.5 of the platform master protocol. The randomisation will be on a 1:1 basis, using a validated computer randomisation system managed through a secure web-based service provided by the Oxford Clinical Trials Research Unit. The randomisation sequence will be generated by the trial statistician. Full details will be stored in a separate randomisation and blinding plan stored in the confidential statistics section of the trial master file.

Randomisation will be performed as close as practicable to the start of surgery as possible to avoid the risk of postponement of surgery or moving to a different theatre list.

9.4 Blinding

This will be a pragmatic randomised comparison so that the treating clinical team cannot be blinded to the treatment allocation. The outcome data will be collected from participants and entered onto the study central database by a research assistant in the study central office to reduce the risk of assessment bias. The participants in this comparison will not be informed which of the two treatments they have received. No formal assessment of the success or otherwise of the blinding will be made.

9.5 Description of the randomised treatments

9.5.1 Preoperative assessments

Participants will usually be assessed in the Emergency Department. Diagnosis of a hip fracture will be confirmed by a plain radiograph, as per routine clinical care. Supplementary imaging will be at the discretion of the treating clinical team. Routine investigations, anaesthetic assessment, antibiotic and venous thromboembolic prophylaxis will be used as per local policy.

9.5.2 Anaesthetic technique

A regional or general anaesthesia technique will be used for every participant as per routine clinical care. Intra-operative analgesia may be achieved by combining a local anaesthetic nerve block using either a

© Copyright: The University of Oxford and Oxford University Hospitals NHS Foundation Trust 2019

nerve stimulator or ultrasound-guided technique, IV paracetamol 1g intravenous infusion and opiate analgesia as clinically indicated. Details of the anaesthetic technique will be recorded in the study CRF.

9.5.3 Surgical treatment

All participants will receive perioperative prophylactic antibiotics in accordance with current protocols agreed at each centre. Appropriate preparation, positioning and anaesthetic technique will be left to the discretion of the clinical team as per their normal clinical practice. Resources related to delivering treatment in both arms will be collected in study case report forms, including type of intervention received, admission and discharge date, complications and further treatments required. Hip replacement will be with an arthroplasty in line with the NICE guidance.[8] Patient position, surgical approach and surgical technique will be chosen by the operating surgeon, details of each will be recorded. The choice of stem and cup components will be at the discretion of the operating surgeon. Participants will be randomly allocated to one of the treatment arms:

1. STANDARD TOTAL HIP REPLACEMENT: The cup and any liner must be of a single articulation, non-constrained design.
2. DUAL MOBILITY TOTAL HIP REPLACEMENT: The cup must be of a dual articulation, non-constrained design.

Details of the operation will be recorded in the study CRF.

9.5.4 Early post-operative care

All participants will be under the care of a multi-disciplinary team with input from a physician with an interest in hip fracture.

After surgery, key aspects of initial rehabilitation will be standardised; all participants will:

1. be encouraged to fully weight bear, and
2. attempt mobilisation on the day of, or first day after, surgery with a therapist.8,24.   The local multi-disciplinary team will be responsible for delivering rehabilitation and managing onward referral and discharge planning as per usual practice, according to local care pathways.

9.6 Assessments

9.6.1 Schedule of assessments

The overall schedule of assessments, including the common outcome set and the additional outcomes measured for this comparison, and methods for data collection are described in the table below:

Time Point	Data	Source	Setting
**Baseline**	i) Demographics<br>ii) Relevant medical history<br>iii) Injury details<br><br>*Pre-injury (Obtained retrospectively):*<br>iv) EQ-5D<br>v) Residential status<br>vi) Mobility status<br>vii) Resource use	Participant/proxy & medical record	Acute inpatient – face to face; medical record review

© Copyright: The University of Oxford and Oxford University Hospitals NHS Foundation Trust 2019

**Up to point of discharge**	i) Resource provision<br>ii) Early complications<br>iii) Index Surgical Treatment information*<br>iv) Dislocation	Medical records for clinical effectiveness analysis.<br>Data linkage with registries and data warehouses for health economics analysis only.	Acute inpatient; Medical record review recorded onto Baseline Additional Hospital CRF
**4 months post-diagnosis of a hip fracture**	i) EQ-5D<br>ii) Re-operation<br>iii) Implant-related infection<br>iv) Dislocation<br>v) Complications<br>vi) Residential status<br>vii) Mobility status<br>viii) Resource use	Participant/proxy<br>Data linkage with registries and data warehouses for health economics analysis only.	Telephone, online or postal
**12 months post-diagnosis of a hip fracture ***	i) EQ-5D<br>ii) Re-operation<br>iii) Implant-related infection<br>iv) Dislocation<br>v) Complications<br>vi) Residential status<br>vii) Mobility status<br>viii) Resource use	Participant/proxy & medical record for clinical effectiveness analysis.<br>Data linkage with registries and data warehouses for health economics analysis only.	Telephone, online or postal; medical record review

Table 1: Assessment schedule, instruments and means of collection.

*Key: *indicates measurement timepoint or data collected is in addition to the Platform Common Dataset specified in the master protocol*

9.6.2 Visits and Contacts

Contact 1: Details of the baseline contact are described in the master platform protocol.

Contact 2: Follow-up at 4 months post-diagnosis of a hip fracture as per platform protocol.

Contact 3: Follow-up at 12 months post-diagnosis of a hip fracture as per the 4-month follow-up time-point.

9.7 Definition of End of Comparison

The end of comparison is the point at which the follow up of the last participant has been completed, all the data has been entered and all queries have been resolved. The last direct data collection will be at 12 (+2) months post-diagnosis of a hip fracture. The Sponsor and main Research Ethics Committee will be notified in writing within 15 days if the comparison has been concluded or terminated early.

10 SAFETY REPORTING

Safety reporting for each participant will begin from the time of consent and will end when the participant has reached their final follow up time point, 12 months post-diagnosis of a hip fracture. Investigators should follow up serious adverse events until resolved or the participant reaches 12 months post-diagnosis of a hip fracture.

Due to the low risk of this randomised comparison and well-established safety profile of the interventions being investigated, adverse events that do not meet the definition of SAEs and unrelated SAEs are not required to be reported.

© Copyright: The University of Oxford and Oxford University Hospitals NHS Foundation Trust 2019

All unexpected serious adverse events (SAEs) are to be reported according to the guidelines specified in section 15 of the Master Protocol.

10.1 Related and expected Serious Adverse Events

See Master Protocol for details of SAEs that are expected and related to the fracture and surgical procedure.

11 STATISTICS & ANALYSES

11.1 Sample size determination

The primary outcome measure, dislocation event, is commonly not collected completely within national registries where the simpler measure – revision surgery for dislocation – is usually preferred. Therefore, the control group level used in the sample size calculation for this comparison was based upon a range of estimates from smaller observational studies that report our primary outcome.[25–29] Control event risks were estimated for the standard THR group of 7, 8 and 9%. We explored various scenarios assuming a true hazard ratio of 0.5, based on previous observational studies and after consultation with our PPI and expert surgeon groups, as a clinically meaningful difference.[13,25,27,30] Assuming random censoring due to death occurring exponentially at 10% per year, a simplified assumption of a constant risk of dislocation during the 12 month follow-up and a (2-sided) significance of 5%, gives:[22,31–33]

Power for 0.5 hazard ratio			
Sample size	Uncensored control risk (%)		
	7	8	9
1000	70	75	80
1600	88	92	95
2000	94	96	98

Under these conditions a total sample of 1600 participants will give approximately 90% statistical power (between 88 and 95% depending upon the control group level) to reject the null hypothesis if the control risk falls within the assumed parameters.

In the UK contribution to DUALITY we anticipate recruiting approximately 450 participants in the time available for recruitment.

11.2 Analysis populations

The primary analysis population will be intention to treat (ITT); that is all participants will be analysed as per their randomised treatment allocation. Sensitivity analyses will be undertaken on the per-protocol population for the primary outcome and key secondary outcomes.

The ITT population includes all randomised participants including:

1. Participants who are randomised but do not undergo surgery (such as those who died or were found to be ineligible after randomisation but before surgery).
2. Participants who are randomised and die after surgery with a consultee declaration signed but before post-diagnosis of a hip fracture consent has been confirmed.
3. Participants who are randomised and found to be ineligible during or after surgery.

© Copyright: The University of Oxford and Oxford University Hospitals NHS Foundation Trust 2019

Note: participants who withdraw from the comparison between randomisation and 12 months will provide data up to the point of withdrawal.

The per protocol population will be the ITT population excluding participants as described in 1 and 3 above and other major deviations from the protocol which will be fully described in the Statistical Analysis Plan.

No within-comparison analyses are planned for the UK subgroup independently from the rest of the DUALITY sample.

11.3 The level of statistical significance

The statistical significance will be assessed at 5% for two-sided tests and reported for p-values less than 5% (p values of less than 0.05). All p-values will be reported to 3 decimal places. 95% confidence intervals will be reported throughout.

11.4 Statistical Analysis

Analyses will be performed using the intention-to-treat principle including all randomised participants according to the allocated treatment. The primary outcome is the adjusted risk of dislocation within 12 months. The cumulative unadjusted incidence of dislocations will be estimated using the Kaplan–Meier method per randomised treatment group. The relative hazard of dislocation in the intervention compared with the control group will be estimated by a Cox regression model adjusted for sex, Body Mass Index and surgical approach. The treatment effect will be presented as a hazard ratio along with a 95% confidence interval and a two-sided likelihood-ratio p-value. With the registry-nested follow-up, we anticipate that there will be complete follow-up (as planned) for all participants, but in the rare case that a participant has incomplete follow-up they will be considered censored at last known follow-up. Death before dislocation will be handled as censoring at day of death.

The secondary endpoints any reoperation, implant-related infection, and all-cause mortality will be analysed and described in the same way as the primary endpoint. Supplementary sensitivity analyses will be performed for all event endpoints. These analyses will primarily use logistic regression with a fixed and curtailed follow-up point with the same covariates as the primary analysis, and as a supplement, risk differences with Wald confidence intervals will be computed. To investigate sensitivity to baseline covariates, unadjusted Cox regression models will be fitted for the primary endpoint. Sensitivity analyses to investigate the impact of censoring by death, in addition to analysing death as an outcome, will include analyses of the composite of dislocation and death performed similarly to the primary endpoint analysis. Randomised and actual treatments will be described in a CONSORT diagram, and additional per-protocol analyses will be undertaken as sensitivity analyses. The threshold of statistical significance will be set at a two-sided p-value of 0.05. Secondary endpoints will be presented without formal multiplicity adjustment.

EQ-5D domain scores (5 levels) at 4 and 12 months after post-diagnosis of a hip fracture will be summarised using descriptive frequency tables by randomised treatment. EQ-5D-5L index values will be derived by mapping the EQ-5D-5L descriptive system data onto the EQ-5D-3L onto the UK utility valuation set using the Crosswalk Index Value Calculator.41 Using this value set the scale ranges from -0.594, indicating the worst possible heath state, to 1.0, and is anchored at 0 and 1.0 indicating a health state equivalent to death and perfect health respectively. Multivariate linear regression will be used to

© Copyright: The University of Oxford and Oxford University Hospitals NHS Foundation Trust 2019

compare EQ-5D-5L utility at 12 months post-diagnosis of a hip fracture between the randomised groups adjusted for sex, BMI, and surgical approach. EQ-5D VAS scores at 12 months post-diagnosis of a hip fracture will be presented using tables of medians and quartiles as well as empirical cumulative distribution plots of VAS score and linear change in VAS from baseline. The VAS score will be analysed using proportional odds logistic regression adjusted for baseline score as a numerical variable modelled as a restricted cubic spline. Zero values will be imputed for all those who have died. For the adjusted analyses, missing baseline scores will be imputed using multiple imputation.

For all event outcome variables, pre-defined subgroup/interaction analyses to assess the homogeneity of the treatment effect will be performed for sex, age, ASA class, and BMI, and for the procedural characteristics femoral neck length, cup diameter, femoral head diameter, type of cup, type of stem, type of cement, and surgical approach. For categorical subgroup indicators, events will be described in each subgroup as for the entire population, and the treatment contrast in each subgroup will be estimated using a Cox proportional hazard model with treatment, subgroup, indicator, and interaction, and presented with nominal 95% confidence intervals for each subgroup and the interaction p-value. For age, sex, and BMI, the interaction model will use restricted cubic spline modelling, and present the results as a curve of treatment effect by covariate with 95% pointwise confidence bands and the interaction p-value. Treatment comparison is not relevant for subgroups that are specific to a single treatment arm. For such subgroups descriptive statistics including Kaplan–Meier plots will be presented for each subgroup.

11.5 Health economic analysis

The economic evaluation will use the data of all participants randomised into the study in both UK and in Sweden, to evaluate the cost-effectiveness of DM versus standard articulation THR from the perspectives of UK NHS and Personal Social Services and the wider society. A cost-utility analysis will compare the incremental health outcome, measured in terms of QALYs, with the incremental cost between the two treatment options following the intention-to-treat principle. The assessment of costs, QALYs and cost-effectiveness will be consistent with methods recommended by the National Institute for Health and Clinical Excellence.[34] Health-related quality of life will be assessed during the comparison period at baseline (a retrospective pre-injury status) and at 4 and 12 months post-diagnosis of a hip fracture using the EQ-5D-5L instrument. The EQ-5D profiles generated for each patient will be valued using a set of estimated preferences based on the UK population using the method recommended by NICE at the time of analysis.[35,36] Multiple imputation techniques will be used to adjust for missing EQ-5D data. Mean and standard errors of EQ-5D utility scores at 4 and 12 months (combined UK and Sweden) by treatment arm will be presented.

Health care resource data will be collected using two key approaches. First, comprehensive hospital episode data will be available for participants in UK and Sweden during the study, both data sources including the index surgical treatment episode and detailed diagnosis and procedure data. Second, UK participants in the comparison will provide further data on their living arrangements, long-term care, other formal and informal care they receive, time off work, house modifications, and mobility equipment at 4 and 12 months in the comparison using patient self-administered questionnaires. Hospital record data of all comparison participants will be mapped into UK Healthcare Resource Groups (HRGs) and costed using UK Reference costs.[37] Mean hospital costs and hospital cost differences between comparison arms will be reported. Costs of personal and social care and work time loss will be evaluated for UK participants using unit costs sourced from the Personal Social Services Research Unit.[38] It is

© Copyright: The University of Oxford and Oxford University Hospitals NHS Foundation Trust 2019

expected that the generalisability of clinical data between UK and Sweden will be appropriate. However, generalisability of resource use and quality of life data between the countries is less certain and will be assessed. The approach to costing hospital care using UK HRGs and costs is expected to ensure hospital care of Swedish participants reflects UK costs. Nevertheless, prior to finalising our assessment methods, we will investigate generalisability of hospital data for THR following hip fracture, separately for the acute admission and hospital rehabilitation care. If substantial differences for elements of resource use and costs are indicated between UK and Sweden, the 450 UK participants will be used to relate level of costs to participant health status; the health status of all participants will be then used to evaluate level of costs during comparison follow-up. Similarly, patient reported data for out of hospital resource use will be available for UK participants only. A regression modelling approach will relate UK participants' health status (presence and type of dislocation/re-operation) with level of personal care, private costs and work loss costs. This model will then inform the economic analysis from the UK NHS and Personal Social Services and, separately, the UK societal perspectives, using health status of all study participants in UK and Sweden during follow-up. Our approach to missing data will depend on the level and pattern of missing data. Cost and QALY data will be combined to evaluate an incremental cost-effectiveness ratio, defined as the ratio of the mean difference in costs to the mean difference in QALYs between treatments with appropriate consideration of different sources of uncertainty. Sensitivity analyses will explore robustness of findings to key parameters such as care costs and utility valuation methods. The health economic analysis plan will be finalised prior to unblinding study data for analysis.

12 DATA PROCESSING AND DATA SHARING

For this randomised comparison, the source data for the clinical data set are defined as the CRFs derived from participant/proxy-report and participants medical records at the recruiting hospital. The clinical data set will be shared with Uppsala University Sweden for clinical effectiveness analysis purposes.

The source data for the health economics data set will be as per the above, augmented with data obtained through approved data linkages. Uppsala University Sweden will share their data set with the University of Oxford to create a combined health economics data set, which will be analysed at the University of Oxford.

The overarching WHiTE Platform, in which this comparison is embedded, has existing approved linkages to National Joint Registry (NJR), Hospital Episode Statistics (HES) and Civil Register (Deaths) described within the master protocol and to which participants consent at the point of recruitment. The purpose of processing these data, in this randomised comparison, will be to confirm correct coding of the source data for, and augmentation of, the health economics data set only. If there is discrepancy between the source data and the additional datasets, the comparison data will be confirmed with the participant/proxy and recruiting hospital. A Data Flow Diagram can be found in Annex B.

Annex C provides a Data Flow Diagram of data sharing between the University of Oxford, UK and The University of Uppsala, Sweden. Pseudonymised source data for the clinical data set only will be shared from University of Oxford to University of Uppsala for the processing purpose of conducting the DUALITY comparison statistical analysis. Details of the data to be shared will be specified in the Data Management Plan (DMP) saved in the central Comparison Master File. Pseudonymised source data only will be shared from University of Uppsala to University of Oxford for the processing purpose of conducting the DUALITY comparison health economic analysis from a NHS perspective.

© Copyright: The University of Oxford and Oxford University Hospitals NHS Foundation Trust 2019

13 DISSEMINATION POLICY

The main outputs for DUALITY will be released within 12 months of obtaining the final data from both the UK and Swedish trials.

The dissemination strategy will consist of three strands: the first will ensure that patients and the public are informed of the trial results; the second will engage practitioners and health-care providers, and the third will inform national guideline and policy makers.

Patients and members of the public: The PPI co-applicant has well-established links with patient groups. We will work in collaboration with Oxford Link and Age UK – East London, led by our PPI co-applicants, to refine and distribute the findings in a plain language summary for patients and the public. In addition to disseminating directly to study participants, findings will be more widely available locally through posters in appropriate outpatient rooms and liaising with identified service user groups.

Health care providers: The final results will be submitted for open access publication, alongside presentations at annual meetings of the British Orthopaedic Association (BOA), Association of Anaesthetists of Great Britain and Ireland (AAGBI), and the Orthopaedic Trauma Society (OTS). We will present the findings to the entire NHS via the NHS national electronic Library for Health (NHS Evidence). International 'reach' of our published research findings will be supplemented by presentations at high visibility meetings such as the OTA Annual Meeting (US) and EFORT Annual Congress (Europe).

National guidelines: We will use our established network involvement to disseminate these research findings. These include the NIHR Clinical Research Network, the International Fragility Fracture Network (a global network aimed at promoting advances in fragility fracture management), and specialist interest groups (BOA/OTS/OTA/EFORT). We expect the results of this definitive trial to be available within 3 years and inform the next NICE clinical guideline (CG124) update in 2025.

© Copyright: The University of Oxford and Oxford University Hospitals NHS Foundation Trust 2019

14 REFERENCES

1. Wolf O, Mukka S, Notini M, Möller M, Hailer NP. Study protocol: The DUALITY trial—a register-based, randomized controlled trial to investigate dual mobility cups in hip fracture patients. *Acta Orthopaedica*. 2020;91(5). doi:10.1080/17453674.2020.1780059

2. Cooper C, Campion G, Melton LJ. Hip fractures in the elderly: A world-wide projection. *Osteoporosis International*. 1992;2(6):285-289. doi:10.1007/BF01623184

3. National Hip Fracture Database (NHFD) annual report 2018 | RCP London. Accessed June 30, 2021. https://www.rcplondon.ac.uk/projects/outputs/national-hip-fracture-database-nhfd-annual-report-2018

4. Johnell O, Kanis JA. An estimate of the worldwide prevalence and disability associated with osteoporotic fractures. *Osteoporosis International*. 2006;17(12):1726-1733. doi:10.1007/s00198-006-0172-4

5. Falls and fracture consensus statement Resource pack. Published online 2017. Accessed July 14, 2021. www.facebook.com/PublicHealthEngland

6. Sullivan PW, Slejko JF, Sculpher MJ, Ghushchyan V. Catalogue of EQ-5D scores for the United Kingdom. *Medical Decision Making*. 2011;31(6):800-804. doi:10.1177/0272989X11401031

7. National Hip Fracture Database (NHFD) annual report 2020 | RCP London. Accessed June 30, 2021. https://www.rcplondon.ac.uk/projects/outputs/national-hip-fracture-database-nhfd-annual-report

8. Overview | Hip fracture: management | Guidance | NICE (CG124). Published online 2017.

9. Jobory A. *Dislocation after Hip Fracture Related Arthroplasty-Incidence, Risk Factors and Prevention*. Lund University, Faculty of Medicine; 2020. Accessed June 30, 2021. https://portal.research.lu.se/portal/en/publications/dislocation-after-hip-fracture-related-arthroplasty--incidence-risk-factors-and-prevention(2a7eacd0-0d11-44c1-a62e-756c6a0cf931)/export.html

10. Evans JT, Blom AW, Timperley AJ, et al. Factors associated with implant survival following total hip replacement surgery: A registry study of data from the national joint registry of england, wales, northern ireland and the isle of man. *PLoS Medicine*. 2020;17(8). doi:10.1371/journal.pmed.1003291

11. Kunutsor SK, Barrett MC, Beswick AD, et al. Risk factors for dislocation after primary total hip replacement: a systematic review and meta-analysis of 125 studies involving approximately five million hip replacements. *The Lancet Rheumatology*. 2019;1(2):e111-e121. doi:10.1016/S2665-9913(19)30045-1

12. Kreipke R, Rogmark C, Pedersen AB, et al. Dual mobility cups: Effect on risk of revision of primary total hip arthroplasty due to osteoarthritis: A matched population-based study using the nordic arthroplasty register association database. *Journal of Bone and Joint Surgery - American Volume*. 2019;101(2):169-176. doi:10.2106/JBJS.17.00841

13. Jobory A, Kärrholm J, Overgaard S, et al. Reduced Revision Risk for Dual-Mobility Cup in Total Hip Replacement Due to Hip Fracture: A Matched-Pair Analysis of 9,040 Cases from the Nordic Arthroplasty Register Association (NARA). *Journal of Bone and Joint Surgery - American Volume*. 2019;101(14):1278-1285. doi:10.2106/JBJS.18.00614

14. Darrith B, Courtney PM, della Valle CJ. Outcomes of dual mobility components in total hip arthroplasty: A systematic review of the literature. *Bone and Joint Journal*. 2018;100B(1):11-19. doi:10.1302/0301-620X.100B1.BJJ-2017-0462.R1

15. Griffin XL, Parsons N, Achten J, Costa ML. A randomised feasibility study comparing total hip arthroplasty with and without dual mobility acetabular component in the treatment of displaced intracapsular fractures of the proximal femur the warwick hip trauma evaluation two : White two. *Bone and Joint Journal*. 2016;98-B(11):1431-1435. doi:10.1302/0301-620X.98B11.BJJ-2016-0478.R1

© Copyright: The University of Oxford and Oxford University Hospitals NHS Foundation Trust 2019

16. Huxley C, Achten J, Costa ML, Griffiths F, Griffin XL. A process evaluation of the WHiTE Two trial comparing total hip arthroplasty with and without dual mobility component in the treatment of displaced intracapsular fractures of the proximal femur: Can a trial investigating total hip arthroplasty for hip fracture be delivered in the NHS? *Bone and Joint Research*. 2016;5(10):444-452. doi:10.1302/2046-3758.510.BJR-2015-0008.R1

17. Blom AW, Rogers M, Taylor AH, Pattison G, Whitehouse S, Bannister GC. Dislocation following total hip replacement: The Avon Orthopaedic Centre experience. *Annals of the Royal College of Surgeons of England*. 2008;90(8):658-662. doi:10.1308/003588408X318156

18. Kotwal RS, Ganapathi M, John A, Maheson M, Jones SA. Outcome of treatment for dislocation after primary total hip replacement. *Journal of Bone and Joint Surgery - Series B*. 2009;91(3):321-326. doi:10.1302/0301-620X.91B3.21274

19. Lenguerrand E, Whitehouse MR, Wylde V, Gooberman-Hill R, Blom AW. Pain and function recovery Trajectories following revision hip arthroplasty: Short term changes and comparison with primary hip arthroplasty in the ADAPT cohort study. *PLoS ONE*. 2016;11(10). doi:10.1371/journal.pone.0164839

20. Total Hip Arthroplasty or Hemiarthroplasty for Hip Fracture. *New England Journal of Medicine*. 2019;381(23):2199-2208. doi:10.1056/nejmoa1906190

21. Burgers PTPW, Hoogendoorn M, van Woensel EAC, et al. Total medical costs of treating femoral neck fracture patients with hemi- or total hip arthroplasty: a cost analysis of a multicenter prospective study. *Osteoporosis International*. 2016;27(6):1999-2008. doi:10.1007/s00198-016-3484-z

22. Caton JH, Prudhon JL, Ferreira A, Aslanian T, Verdier R. A comparative and retrospective study of three hundred and twenty primary Charnley type hip replacements with a minimum follow up of ten years to assess wether a dual mobility cup has a decreased dislocation risk. *International Orthopaedics*. 2014;38(6):1125-1129. doi:10.1007/s00264-014-2313-2

23. Tabori-Jensen S, Frølich C, Hansen TB, Bøvling S, Homilius M, Stilling M. Higher UHMWPE wear-rate in cementless compared with cemented cups with the Saturne® Dual-Mobility acetabular system. *HIP International*. 2018;28(2):125-132. doi:10.1177/1120700018768615

24. Hip fracture rehabilitation in physiotherapy practice | The Chartered Society of Physiotherapy. Accessed February 17, 2022. https://www.csp.org.uk/publications/hip-fracture-rehabilitation-physiotherapy-practice

25. Hailer NP, Weiss RJ, Stark A, Kärrholm J. The risk of revision due to dislocation after total hip arthroplasty depends on surgical approach, femoral head size, sex, and primary diagnosis. *Acta Orthopaedica*. 2012;83(5):442-448. doi:10.3109/17453674.2012.733919

26. de Martino I DRSVPLSPSTP. Dislocation following total hip arthroplasty using dual mobility acetabular components. *https://doi.org/101302/0301-620X99B1BJJ-2016-0398R1*. 2017;99B(1):18-24. doi:10.1302/0301-620X.99B1.BJJ-2016-0398.R1

27. Tarasevičius S, Robertsson O, Dobozinskas P, Wingstrand H. A comparison of outcomes and dislocation rates using dual articulation cups and THA for intracapsular femoral neck fractures. *HIP International*. 2013;23(1):22-26. doi:10.5301/HIP.2013.10632

28. Hailer NP, Weiss R, Stark A, Karrholm J. Dual-mobility cups for revision due to instability are associated with a low rate of re-revisions due to dislocation: 228 patients from the Swedish Hip Arthroplasty Register. *Acta orthopaedica*. 2012;83(6):566-571. doi:10.3109/17453674.2012.742395

29. A, Brüggemann H, Mallmin NP H. Do dual-mobility cups cemented into porous tantalum shells reduce the risk of dislocation after revision surgery? *Acta orthopaedica*. 2018;89(2):156-162. doi:10.1080/17453674.2018.1432927

30. Bensen AS, Jakobsen T, Krarup N. Dual mobility cup reduces dislocation and re-operation when used to treat displaced femoral neck fractures. *International Orthopaedics*. 2014;38(6):1241-1245. doi:10.1007/s00264-013-2276-8

© Copyright: The University of Oxford and Oxford University Hospitals NHS Foundation Trust 2019

31. Sims AL, Parsons N, Achten J, Griffin XL, Costa ML, Reed MR. A randomized controlled trial comparing the Thompson hemiarthroplasty with the Exeter polished tapered stem and Unitrax modular head in the treatment of displaced intracapsular fractures of the hip. *Bone and Joint Journal*. 2018;100B(3):352-360. doi:10.1302/0301-620X.100B3.BJJ-2017-0872.R2

32. Fernandez MA, Achten J, Lerner RG, et al. Randomised controlled trial comparing hydroxyapatite coated uncemented hemiarthroplasty with cemented hemiarthroplasty for the treatment of displaced intracapsular hip fractures: A protocol for the WHITE 5 study. *BMJ Open*. 2019;9(12). doi:10.1136/bmjopen-2019-033957

33. Herdman M, Gudex C, Lloyd A, et al. Development and preliminary testing of the new five-level version of EQ-5D (EQ-5D-5L). *Quality of Life Research*. 2011;20(10):1727-1736. doi:10.1007/s11136-011-9903-x

34. *Guide to the Methods of Technology Guide to the Methods of Technology Appr Appraisal 2013 Aisal 2013 Process and Methods*.; 2013.

35. van Hout B, Janssen MF, Feng YS, et al. Interim scoring for the EQ-5D-5L: Mapping the EQ-5D-5L to EQ-5D-3L value sets. *Value in Health*. 2012;15(5):708-715. doi:10.1016/j.jval.2012.02.008

36. Hernandez Alava M, Wailoo A, Grimm S, et al. EQ-5D-5L versus EQ-5D-3L: The Impact on Cost Effectiveness in the United Kingdom. *Value in Health*. 2018;21(1):49-56. doi:10.1016/j.jval.2017.09.004

37. *National Cost Collection: National Schedule of NHS Costs - NHS Trust and NHS Foundation Trusts (2018-19).* Accessed June 30, 2021. https://www.england.nhs.uk/national-cost-collection/

38. Curtis LA, Burns A. Unit Costs of Health and Social Care 2019. Accessed June 30, 2021. https://kar.kent.ac.uk/79286/

© Copyright: The University of Oxford and Oxford University Hospitals NHS Foundation Trust 2019

ANNEX A: FLOW CHART

Inclusion

- Patients ≥ 65 years with displaced intracapsular hip fracture who meet the accepted criteria for the consideration of a THR as per current NICE guidance.

Exclusion

- Delayed fracture surgery (date of presentation more than seven days prior to date of randomisation).
- Pathological or stress fracture of the femoral neck.
- Fracture adjacent to a previous ipsilateral hip implant, such as a previously inserted screw or plate.

Platform Exclusion

- Previous participation in the same randomised comparison
- A second hip fracture (other side) while the patient is still enrolled in the Platform following their first hip fracture.

↓

Randomisation (1:1)
Remote web-based randomisation with RRAMP

↓ ↓

Standard Total Hip Replacement:
The cup and any liner must be of a single articulation, non-constrained design.

Dual Mobility Total Hip Replacement:
The cup must be of a dual articulation, non-constrained design.

↓ ↓

Baseline data collection

- WHiTE Platform Baseline CRFs.
- DUALITY Additional Baseline CRFs

↓

Follow-Up 4 Months – Telephone, Post or electronic, and medical records

- EQ-5D-5L, complications (including re-operation, implant-related infection and dislocation), residential status, subjective mobility status, resource use, collected by DUALITY trial office.
- Complications recorded up to 4 months post-diagnosis of a hip fracture (reported by patients and research team).

Follow-Up 12 Months – Telephone, Post or electronic, and medical records

- EQ-5D-5L, complications (including re-operation, implant-related infection and dislocation), residential status, subjective mobility status, resource use, collected by DUALITY trial office.
- Complications recorded up to 12 months post-diagnosis of a hip fracture (reported by patients and research team).

© Copyright: The University of Oxford and Oxford University Hospitals NHS Foundation Trust 2019

ANNEX B: Data linkage planned within UK

Data linkage already in place under the WHITE PLATFORM
Participants consent to data sharing at point of recruitment.
Purpose of linkage for DUALITY is to confirm correct coding of the source data for, and augmentation of health economics data set.

© Copyright: The University of Oxford and Oxford University Hospitals NHS Foundation Trust 2019

ANNEX C: Data sharing of pseudonymised trial source data between Universities of Oxford and Uppsala

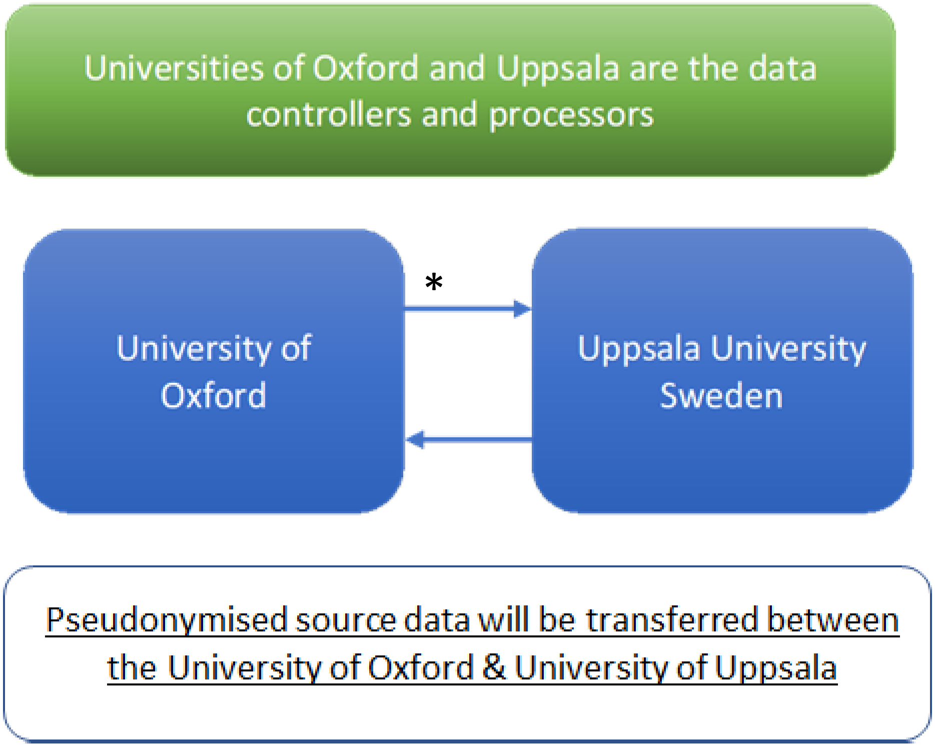

*Clinical dataset for clinical effectiveness analysis.

© Copyright: The University of Oxford and Oxford University Hospitals NHS Foundation Trust 2019
