# Supplementary material for: Dual mobility versus standard cups in total hip replacement for displaced femoral neck fractures (Duality): an international, multicentre, randomised, controlled, superiority trial
Source: Lancet. 2026 Jul 25;408(10552):348–56. doi: 10.1016/S0140-6736(26)00759-2 (PMC13425373; doi:10.1016/S0140-6736(26)00759-2)
Supplement: Supplementary appendix [file mmc4.pdf]

THE LANCET

Supplementary appendix 4

This appendix formed part of the original submission. We post it as supplied by the authors.

Supplement to: Hailer NP, Griffin XL, Mukka S, et al. Dual mobility versus standard cups in total hip replacement for displaced femoral neck fractures (Duality): an international, multicentre, randomised, controlled, superiority trial. *Lancet* 2026; published online July 2. https://doi.org/10.1016/S0140-6736(26)00759-2.

Statistical Analysis Plan

Study Code:	DUALITY
Study Title:	The DUALITY trial—a register-based, randomised controlled trial to investigate dual mobility cups in hip fracture patients
Based on protocol version and date:	3.1, 18-JAN-2024
Biostatistician:	Tatevik Ghukasyan Lakic
Clinical Data Manager:	
Clinical Project Leader:	
Sponsor:	Uppsala University Hospital

Approved by Study Biostatistician (UCR);

Name: Tatevik Ghukasyan Lakic Signature: Date:

Reviewed and approved by Biostatistician (UCR);

Name: Ollie Östlund Signature: Date:

Approved by Clinical Project Leader;

Name: NA Signature: Date:

Approved by Sponsor's Representative;

Name: Nils Hailer Signature: Date:

Table of Contents

Statistical Analysis Plan 1
1 Introduction 3
2 Abbreviations 3
3 Study Objectives and Endpoints 4
3.1 Objectives 4
3.1.1 Primary Objective 4
3.1.2 Secondary Objectives 4
3.2 Endpoints 4
3.2.1 Primary Endpoint(s) 4
3.2.2 Secondary Endpoint(s) 4
4 Study Design 5
4.1 Study Subjects and Eligibility Criteria 5
4.2 Randomisation and Blinding 6
4.3 Surgical Intervention 6
4.4 Withdrawal of Patients from the Trial 6
5 Definition of Analysis Populations 6
6 Description of Statistical Analysis 7
6.1 Study Conduct and Subject/Patient Disposition 7
6.2 Baseline Characteristics and Treatment Group Comparability 7
6.3 Treatment Administration/Adherence 7
6.4 Efficacy Analyses 8
6.4.1 General considerations and multiplicity issues 8
6.4.2 Event Outcomes 8
6.4.3 Primary outcome 9
6.4.4 Secondary event outcomes 9
6.4.5 Subgroup analyses of event outcomes 10
6.4.6 Secondary SLR outcomes 11
6.5 Safety Analyses 12
6.6 Handling of Missing Data 12
7 Determination of Sample Size 12
8 Changes in the Planned Analysis 13
9 Description of Data Sources and Derived Variables 13
9.1 Data Sources 13
9.2 Derived Variables 14
10 Description of Output 14
11 Statistical Software 14

1 Introduction

The aim of this statistical analysis plan (SAP) is to describe details of planned presentation of the study data described to be performed by the Uppsala Clinical Research (UCR) Biostatistics section. The results will be presented according to the output specification (see Appendix 1 Output Shells). The UCR study biostatistician is responsible for writing the plan with necessary input from other members of the study team. A biostatistician and the coordinating investigator will approve the final version. The SAP is based on trial protocol version 3.3, dated 2025-02-26.

2 Abbreviations

BMI	Body Mass Index
CI	Confidence Interval
DBC	Database Closure Document
DMC	Dual Mobility Cups
FNF	Femoral Neck Fracture
HR	Hazard Ratio
ICD	International Classification of Diseases
ITT	Intention to Treat
mITT	Modified Intention to Treat
NJR	National Joint Registry
NPR	National Patient Register
PJI	Periprosthetic Joint Infection
PP	Per Protocol
R-RCT	Registry-based Randomised Clinical Trial
SAP	Statistical Analysis Plan
SAR	Swedish Arthroplasty Register (Ledprotesregistret)
THA	Total Hip Arthroplasty
UCR	Uppsala Clinical Research Center

3 Study Objectives and Endpoints

3.1 Objectives

3.1.1 Primary Objective

The primary aim of the DUALITY trial is to investigate whether the use of dual mobility cups (DMC) reduces the risk of dislocation after total hip arthroplasty (THA) surgery performed due to femoral neck fracture (FNF) when compared with standard cups.

3.1.2 Secondary Objectives

The secondary objectives of this study are to evaluate whether:

1. there is an increased risk of other adverse events such as
   - re-operations (for any reason),
   - periprosthetic joint infections (PJI),
   - mortality,

after the use of DMC within one year and within three years.

2. patient-reported outcomes are improved after one year by the use of DMC.
3. the use of DMC is cost-effective.

Note: cost-effectiveness analyses are outside the scope of this SAP.

3.2 Endpoints

3.2.1 Primary Endpoint(s)

The primary endpoint is the occurrence of any dislocation treated with closed or open reduction of the index joint within 1 year.

3.2.2 Secondary Endpoint(s)

Secondary endpoints are:

- Any reoperation of the index joint within 1 year,
- PJI of the index joint within 1 year,
- Mortality within 90 days and within 1 year,
- Composite of dislocation and mortality within 1 year[1],

Patient-reported outcomes at 1 year will be assessed by use of

- EQ-5D domain score (5 levels), the 5 dimensions for describing health states will be analysed separately

[1]Not listed as an outcome in protocol, but included in the statistical methods section

  - Mobility
  - Usual Activities
  - Self-care
  - Pain & Discomfort
  - Anxiety & Depression
- EQ-5D index
- EQ-5D-visual analogue scale (VAS) on a 0–100 numeric scale.

Note: In addition to the planned analyses at one year, both primary and secondary time-to-event outcomes will also be analysed at three years when the longer follow-up data is available.

4 Study Design

The DUALITY trial is a multicenter, register-nested, randomised controlled trial (rRCT; James et al. 2015). Patients with a displaced FNF who are eligible for a THA according to local guidelines are randomised 1:1 to intervention (DMC) or control treatment (standard cup).

4.1 Study Subjects and Eligibility Criteria

The SFR study platform detects eligible patients during registration of the injured patient and automatically alerts the admitting physician of the possibility to screen the patient for eligibility, a method of register-based screening and inclusion that is also used for the first orthopedic rRCT in Sweden, the HipSTHer trial (Wolf et al. 2020).

Inclusion criteria are:

- age ≥ 65 years
- fracture type 3 or 4 according to Garden or basicervical femoral neck fracture, classified in the SFR as 31-B2 or B3
- eligibility for a THA according to local guidelines and routines,
- availability of both treatment options, and
- signed, informed patient consent.

Unavailability of both treatment options can be due to implants being out of stock or the lack of the individual surgeon’s competence to use either implant type.

Exclusion criteria are:

- cognitive impairment,
- previous inclusion of a contralateral THA in the ongoing trial,
- delayed fracture surgery (date of injury more than seven days prior to date of screening),
- pathological or stress fracture of the femoral neck,
- inability or unwillingness to give written consent,
- unavailability of both interventions for a study subject, and
- fracture adjacent to a previous ipsilateral hip implant, such as a previously inserted screw or plate.

4.2 Randomisation and Blinding

Randomisation is performed by use of the study platform incorporated in the SFR. Subjects are randomised to receive either a DMC (intervention group) or a standard cup (control group), using an allocation sequence hidden from all involved healthcare providers and provided by a trial-independent statistician. There will be no patient or physician blinding.

4.3 Surgical Intervention

The trial design is pragmatic, which implies that the choice of implant brands, fixation methods, surgical approach, pre-, peri-, and postoperative routines are based on the participating hospitals' preferences. Nonetheless, the study protocol requires all participating units to maintain their chosen regime across both intervention and control groups, thus ensuring that only the type of intervention varies per unit. In Sweden, two-thirds of FNF patients who receive a THA are operated on via a direct lateral approach (Swedish Hip Arthroplasty Register 2018) according to Hardinge (1982) or Gammer (1985), and the remaining third via a posterior approach. Surgical approach can vary by surgeon, but individual surgeons must maintain the same approach for both study groups. If the posterior approach is used, the posterior capsule and short external rotators should be repaired, but if individual surgeons choose to abstain from this recommendation, they are free to do so, provided they maintain this regime across treatment groups.

4.4 Withdrawal of Patients from the Trial

Participants are free to withdraw from the trial at any time without any adverse consequences to further treatment. Already collected data on patients who choose to withdraw their consent to participate in the trial will be retained in the study database, but no additional data including data derived from cross-matching of the SFR database with other registries will be added.

5 Definition of Analysis Populations

mITT: All intentionally randomised patients possible to identify for follow-up and not breaking critical (as defined by the sponsor) inclusion criteria will be included in the modified Intention-to-treat (mITT) population. Randomisation numbers assigned unintentionally, such as by clicking the wrong box or randomising for the wrong patient record in SFR, will be recorded in the clean file documentation and removed from the analysis database. Attempts will be made for randomisation numbers with incorrect patient identifiers to identify if there is an intentionally randomised patient linked to the number. A randomisation number for which it is not possible to clearly identify a patient for follow-up of primary endpoint, will be recorded and removed from the analysis database, even if a patient might have been randomised. M-ITT population will be the primary efficacy dataset and participants will be presented in the treatment group to which they were randomised (even if they received the other cup).

PP: The Per Protocol (PP) population will be a subset of the mITT population. All patients with relevant protocol deviation will be excluded from this dataset. This dataset will be used for sensitivity analyses of the primary efficacy endpoint and secondary event outcomes, and participants will be presented in the treatment group they received (regardless of randomisation).

Relevant protocol deviations are defined as

1. Patients not clearly documented to have received the device they were randomised to as initial intervention,
2. Patients known to have been included despite satisfying exclusion criteria.

Patients not included in the mITT and PP population will be identified and listed in database closure document (DBC).

6 Description of Statistical Analysis

6.1 Study Conduct and Subject/Patient Disposition

Descriptive statistics of inclusion and follow-up will be presented by randomised treatment. Inclusion will be illustrated graphically in a cumulative plot. Withdrawals, with reason for withdrawal, and loss to follow-up will be displayed in a CONSORT diagram. The time to last follow-up of withdrawals will also be presented in a listing.

6.2 Baseline Characteristics and Treatment Group Comparability

Baseline characteristics will be tabulated descriptively in total and by randomised treatment, for the mITT analysis set, for the mITT analysis set by sex, surgical approach and country subgroups, and for the PP analysis set.
Categorical variables will be described as number and percentage by randomised treatment. Missing data will be presented as a separate category. Numerical variables will be presented as number of observations with data, median, quartiles, min and max, and arithmetic mean and standard deviation.

6.3 Treatment Administration/Adherence

Deviations from protocol concerning the cup will be presented as total number and percentage of patients by deviation type, and also by deviation type and reason. All DUALITY free-text comments will be listed.

6.4 Efficacy Analyses

6.4.1 General considerations and multiplicity issues

All analyses will be by randomised treatment. The primary analysis of the primary outcome will be considered significant if $p<0.05$ (two-sided). There is no pre-specified testing procedure after the primary test, and all inferential results will be presented with nominal confidence intervals (CI) and p-values, without multiplicity adjustment. Inferential results for secondary outcomes should be interpreted with caution.

6.4.2 Event Outcomes

6.4.2.1 Analysis set for event outcomes

Analysis will be performed using the intention-to-treat principle including all patients in mITT set according to randomised treatment. Only the first event of each type will be presented, not repeated events in the same patient.
For PP sensitivity analyses the PP population will be used.

6.4.2.2 Follow-up and censoring for event outcomes

Time to event for all outcomes will be from randomisation. Participants who withdraw their consent will be considered censored on the date of withdrawal. However, such patients may only have complete data up to the last previous extraction of registry data or follow-up contact. In that case the date of last data extraction will be used. If registry data has been obtained for participants after withdrawal from follow-up, events and follow-up time after the date of withdrawal will not be included in the analysis data base, and in analyses the participant will be considered censored on the date of withdrawal. For UK patients the NJR data can be used if the withdrawal only refers to clinical follow-up. Recorded events after the end of each follow-up will not be included in the analyses. In the unlikely event that different event types have different follow-up time for a participant, the shortest time will be used for composites, and events after that time will be censored. For endpoints that do not include mortality, patients that die before the last day of follow-up without reaching an endpoint will be considered censored on the day of death, to estimate the endpoint cause-specific hazard ratio (HR).

Patients without event or other censoring will be censored on day 365.

6.4.2.3 Descriptive statistics for event outcomes

All event outcomes will be presented as Kaplan-Meier plots and tables of number and percentage of participants with event and events by patient year, by randomised treatment for all follow-up time. Censored patients (see above) will be considered not to have had an event, and included in the denominator for percentages. Time after censoring or event will be excluded from the denominator for event rates.

6.4.2.4 Statistical analysis of event outcomes

All event outcomes will be analysed using Cox proportional hazards regression adjusted for sex, surgical approach, country and age (included as continuous variable), and the treatment contrast will be presented as a hazard ratio with 95% confidence interval and two-sided p-value for the hypothesis of no difference. Ties will be handled with Efron's method and tests and confidence intervals will be based on the likelihood ratio.

6.4.3 Primary outcome

6.4.3.1 Primary analysis

The primary outcome, time from randomisation to first dislocation treated by open or closed reduction, will primarily be presented and analysed as described in section 6.4.2.

6.4.3.2 Sensitivity and supplemental analyses

Supplementary analyses will be performed for all event endpoints. These analyses will primarily use logistic regression with the same covariates as the primary analysis. Patients that die without an endpoint will be considered as non-events.
In addition, risk differences with Wald confidence intervals will be computed using Kaplan-Meier point estimates and standard errors at 1 year.
To investigate sensitivity to baseline covariates, unadjusted Cox regression models will be fitted. Sensitivity analyses to investigate the impact of possibly informative censoring by death, in addition to analyzing death as an outcome, will include analyses of the composite of dislocation and death performed similarly to the primary endpoint analysis.
Cumulative hazards will be plotted on linear and log-scale, by treatment, to aid assessment of proportional hazards. No statistical test for non-proportionality will be performed.
Additional post-hoc analyses may be considered in case of suspected severely violated proportional hazards assumption, such as repeating the primary analysis of primary event outcome with censoring at 30 and 90 days. This allows comparisons of short- and long-time effects, and gives estimates that can be used for different time horizons if proportional hazards seem severely violated. Care must be exercised to interpret the censored analyses due to multiplicity. Additional analyses may include restricted mean survival time analysis.

6.4.4 Secondary event outcomes

Secondary event outcomes; any reoperation of the index joint, PJI of the index joint, mortality, as well as the composite of dislocation/mortality will be presented as described in section 6.4.2.

6.4.5 Subgroup analyses of event outcomes

Subgroup analyses will be performed for all event outcomes. Patients with missing data for the subgroup indicator will be excluded. Since the subgroup is defined by pre-randomisation characteristics, this is assumed not to induce bias. The purpose of the analyses is to explore possible heterogeneity in treatment effect, and no subgroup-specific claims will be made. Data descriptions will include Kaplan-Meier plots by treatment and subgroup, and tables of number of patients and events in each arm in each subgroup. All subgroup analyses will be performed using interaction models based on the primary analysis model, also including the subgroup in question and the treatment-subgroup interaction as factors and presented as treatment hazard ratios with nominal 95% confidence intervals for each subgroup and the interaction p-value.
The following subgroups were pre-defined in the protocol.

- Sex
- Age
- ASA class
- BMI
- Country[2]

and the procedural characteristics

- Femoral neck length
- Cup diameter
- Femoral head diameter
- Type of cup
- Type of stem
- Type of cement
- Surgical approach

For age and BMI, the interaction model will use restricted cubic spline modelling with 4 knots placed at 5th, 35th, 65th and 95th percentiles, and present the results as a curve of treatment contrast by covariate with 95% pointwise confidence bands and the interaction p-value. For descriptive purposes, such as in forest plot, BMI will also be categorised using the levels below:

  - Underweight: BMI less than 18.5
  - Normal weight: BMI 18.5–24.9
  - Overweight: BMI 25.0–29.9
  - Obesity: BMI 30.0–34.9
  - Severe obesity: BMI 35.0 and above.

Treatment comparison is not relevant for subgroups that are specific to a single treatment arm. For such subgroups descriptive statistics including Kaplan–Meier plots will be presented for each subgroup. The subgroups analyses will use observed cases, without any imputation of missing subgroup variable.

[2] Added while developing this SAP, explained by inclusion of UK sites after protocol development.

6.4.6 Secondary SLR outcomes

6.4.6.1 EQ-5D dimension scores

EQ-5D domain scores (5 levels) at 1 year after index surgery will be summarised using tables with number and percentage of patients in each reported class including missing data as a separate category, and the same table but for observed cases only (excluding patients with missing data), by randomised treatment. These outcomes will be analyzed by using proportional odds logistic regression models adjusted for the baseline domain score as a categorical variable. The results will be presented as the common odds ratio of higher domain score, together with 95% CI and two-sided p-value. Missing domain scores due to death will be considered a separate category. For the primary analysis, missing baseline scores will be imputed using the most frequent value (mode). Sensitivity analyses using observed cases only will also be provided.

6.4.6.2 EQ-5D VAS

EQ-5D VAS score at 1 year after index surgery will be tabulated by randomised treatment, including number of missing observations, arithmetic mean, standard deviation, median, quartiles and max and min values as well as empirical cumulative distribution plots of VAS score and linear change in VAS from baseline. The VAS score will be analyzed using proportional odds logistic regression adjusted baseline score as a numerical variable modelled as a restricted cubic spline with four knots placed at 5th, 35th, 65th and 95th percentiles. Missing baseline scores will be imputed using the median value. Outcome scores that are missing due to death will primarily be imputed as 0, with no imputation of other missing scores.

6.4.6.3 EQ-5D index

EQ-5D index at 1 year will be tabulated by randomised treatment, including number of missing observations, arithmetic mean, standard deviation, median, quartiles and max and min values. The recommended UK valuation method for EQ-5D-5L will be used to calculate the value for each patient at respective time point[3]. We considered both the Swedish and UK mapping algorithms and chose to use the UK mapping to ensure consistency with the health economic analysis plan. As a sensitivity analysis, we will also use the Swedish valuation[4]. The outcome index will be analysed using linear regression models with robust standard errors adjusted for baseline measurement. In case of severly violated model assumptions other methods such as proportional odds models will be considered as sensitivity analyses.

---

[3] Hernandez Alava M, P.S., Wailoo A.,, Estimating the Relationship Between EQ-5D-5L and EQ-5D-3L: Results from a UK Population Study. Pharmacoeconomics 2023; 41(2): 199-207

[4] Burström K, Teni FS, Gerdtham UG, Leidl R, Helgesson G, Rolfson O, Henriksson M. Experience-Based Swedish TTO and VAS Value Sets for EQ-5D-5L Health States. Pharmacoeconomics. 2020 Apr 20.

6.5 Safety Analyses

The secondary event outcomes will be treated as safety outcomes and analysed as described in section 6.4.2

6.6 Handling of Missing Data

The use of a register in the data collection and the type of subjects in the study indicate that the amount of missing data for key hard endpoints are likely to be small (i.e. <2%).

For the primary analyses of primary outcome missing data in surgical approach variable will be imputed using the most frequent value at respective site.

For the outcome EQ-5D (5 levels) missing baseline scores will be imputed using the most frequent value. Outcome scores that are missing due to death will be considered a separate category and coded as 6.
For the outcome EQ-5D VAS/index missing outcome scores due to death will primarily be imputed as 0, and missing baseline scores will be imputed using the median/mean value.

No other imputation of missing outcome scores will be performed for EQ-5D variables.

7 Determination of Sample Size

Scenario 1 For our power calculation, we assume that the 1-year incidence of dislocation after insertion of a standard THA after FNF is 7%, thus slightly lower than the 8% dislocation rate described in Swedish FNF patients treated with a THA (Jobory 2020). For the intervention group operated on with a DMC we assume a relative risk of 0.5, giving an incidence of dislocation of 3.6%. This risk reduction is based on the relative risk of dislocations after the use of a DMC estimated in previous observational studies, ranging from 0.3 to 0.5 (Hailer et al. 2012, Tarasevicius et al. 2013, Bensen et al. 2014, Jobory et al. 2019).

Scenario 2 A recent study from Denmark that investigates the use of DMC in FNF patients reports a dislocation rate of 4.7% after a mean follow up of 5.4 years (Tabori-Jensen et al. 2019). To account for this alternative, more pessimistic scenario, we calculate power based on the assumption of a 1-year dislocation rate of 8% in the control group and 4.5% in the intervention group, giving a relative risk of 0.55. Sample size was determined by simulations under a simplified assumption of a constant risk during the 1-year follow-up, with 25% of the control-arm patients having an event risk of 6.4% (no risk factors), 50% of patients having a 7.4% event risk (one risk factor), and 25% of patients having a 8.5% event risk (two risk factors), corresponding to sex and surgical approach as independent risk factors associated with an increased risk of dislocation (Hailer et al. 2012). Random censoring due to death was assumed to occur exponentially at 10%/year. This assumption is based on a Swedish study on hip fracture patients treated with a THA (Hailer et al. 2016) and is also in line with mortality data in patients treated with THA due to FNF that is reported by the SHAR (Swedish Hip Arthroplasty Register 2018). With a sample size of n

= 1,600 patients, the trial has 88% power to detect a reduction in 1-year dislocation rates from 7% to 3.6%, equaling a hazard ratio of 0.5 (scenario 1), and 83% power to detect a reduction from 8% to 4.5%, equaling a hazard ratio of 0.55 (scenario 2).

8 Changes in the Planned Analysis

Primary analyses of all time-to-event outcomes will be adjusted for sex, surgical approach, and country, with two levels – Sweden and UK. The addition of country variable to the previously listed sex and surgical approach variables is motivated by the inclusion of UK sites after the protocol development. We decided to also include age in the adjustment variables, as age is believed to be predictive for secondary outcomes, and we aimed to maintain consistency across models for all time-to-event outcomes.

According to the protocol, all randomised patients were to be included in the primary efficacy dataset following the intention-to-treat principle. However, the steering committee identified patients who were randomised but subsequently found not being eligible for the DUALITY trial. We also identified patients who were operated with internal fixation who could consequently not experience the primary outcome (dislocation of hip arthroplasty) and for whom it also was not possible to adjust for surgical approach, why these were also excluded. These patients will be identified and documented in clean file, and excluded from the analysis dataset. Thus the primary efficacy dataset will be based on the mITT population.

The SAP specifies single imputation for missing baseline values in EQ-5D variables instead of multiple imputation, as originally stated in the protocol. This change was made because the outcome in question is secondary, and single imputation is deemed sufficient for handling missing baseline data in this context.

9 Description of Data Sources and Derived Variables

9.1 Data Sources

All study data from Swedish sites will be transferred from SFR, SAR and the National Patient Register (NPR) into the study database, with the exception of the screening question answers that will be entered into the study database from the SFR interface. Data relevant to assess known confounders and primary and secondary outcomes will be collected retrospectively from the registries mentioned above.
In the SFR all fracture types in adults and all long-bone fractures in children are registered since 2011. The SFR is a unique national quality register as it contains information on fractures, regardless of treatment (surgical or non-surgical). The SAR is a merger of two national arthroplasty registers, the Swedish Knee Arthroplasty Register and the Swedish Hip Arthroplasty Register. The register includes data about knee replacements since 1975, hip replacements since 1979, hemiarthroplasties since 2005 and knee osteotomies since

2013. There is data about patient reported outcome, demography, prophylaxis, surgical technique, type of implant, reoperations and revisions.

The NPR includes, among other information, data on all completed inpatient stays since 1964, with nationwide coverage beginning in 1987. The data to be retrieved from the NPR will include the main diagnosis, secondary diagnosis, external cause of injury, procedures together with corresponding dates, and the dates of admission and discharge for each visit.

Baseline data on age, sex, injury mechanism, fracture classification, time of diagnosis obtained by radiography, and time and type of surgical treatment are transcribed from the SFR to the study database. Answers given by the admitting physician in response to the screening questions will be saved to the study database in order to enable an analysis of reasons underlying the failure to include eligible patients in the trial. Postoperatively, procedural details on the type of surgical approach, type of cup and stem fixation, cement brands, cup and stem brands, cup and femoral head diameter, femoral neck length, and stem size are registered in the SAR according to national routines. In addition to these procedural details body mass index (BMI) and American Society of Anesthesiologists (ASA) class are recorded in the SLR. After trial completion, the study cohort obtained from the SFR will be linked to information on the study participants registered in the SAR and the NPR, and these data will be entered into a common research database.

Data from UK sites will be securely transferred to UCR and harmonised with the study database. Further details on the harmonisation process will be provided in the documen DUALITY final SAP (DV) v 1.0.

9.2 Derived Variables

Derived variables are described in the document DUALITY final SAP (DV) v 1.0.

10 Description of Output

Output shells are found in DUALITY final SAP (OS) v 1.0. Note: This document does not include analyses at three years; however, these analyses will be conducted in the same manner as the one-year time-to-event outcome analyses.

11 Statistical Software

The software packages R 4.1.2 (R Foundation for Statistical Computing, Vienna, Austria) and SAS 9.4 SAS Institute, Inc., Cary, NC will be used for data management and the statistical analyses.

Appendix 1 – Output Shells

Statistical Analysis Plan: Output shells

Study Code:	DUALITY
Study Title:	The DUALITY trial—a register-based, randomised controlled trial to investigate dual mobility cups in hip fracture patients
Based on protocol version and date:	3.3, 26-FEB-2025
Biostatistician:	Tatevik Ghukasyan Lakic

Reviewed and approved by Biostatistician (UCR);

Name: Ollie Östlund	Signature:	Date:

Approved by Sponsor's Representative;

Name: Nils Hailer	Signature:	Date:

SAP version: final 1.0

1. Description of Output

Table 1. Inclusion dates by country

Country	Randomised treatment	N	First patient in	Last patient in
Sweden	DMC			
	Control			
UK	DMC			
	Control			

Table 2. Subject disposition

Disposition	Randomised Treatment		Total
	DMC N	Control N	N
Subjects screened			x
Subjects randomised in the study	x	x	x
*Subjects withdrawn from the study, n (%)**	x (x)	x (x)	x (x)
*- due to x***	x(x)	x (x)	x (x)
*- due to xx***	x(x)	x (x)	x (x)
*- due to xxx***	x(x)	x (x)	x (x)
*Subjects included in mITT Population**	x(x)	x (x)	x (x)
*Subjects included in PP Population**	x(x)	x (x)	x (x)

*the denominator is the number of randomised subjects
** the denominator is the number of withdrawn subjects

Table 3. Protocol deviations

Disposition	Randomised Treatment		Total N
	DMC N	Control N	
*Subjects excluded from PP, n (%)**	x (x)	x (x)	x (x)
*- due to wrong fracture type***	x(x)	x (x)	x (x)
*- due to wrong treatment***	x(x)	x (x)	x (x)
*- due to xxx***	x(x)	x (x)	x (x)
*Subjects included in mITT Population**	x(x)	x (x)	x (x)
*Subjects included in PP Population**	x(x)	x (x)	x (x)

*the denominator is the number of randomised subjects
** the denominator is the number of withdrawn subjects

Table 4. Number of subjects by site

Disposition	N screened	N included	N included (% of screened)	N included in PP
Sweden, n (%)				
- Uppsala				
- Gävle				
- Halmstad				
- Etc…				
UK, n (%)				
- Royal Cornwall Hospital				
- Conquest Hospital				
- Etc…				

Figure 1. Inclusion by country, ITT [Example plot. Will be plotted for Sweden and UK].

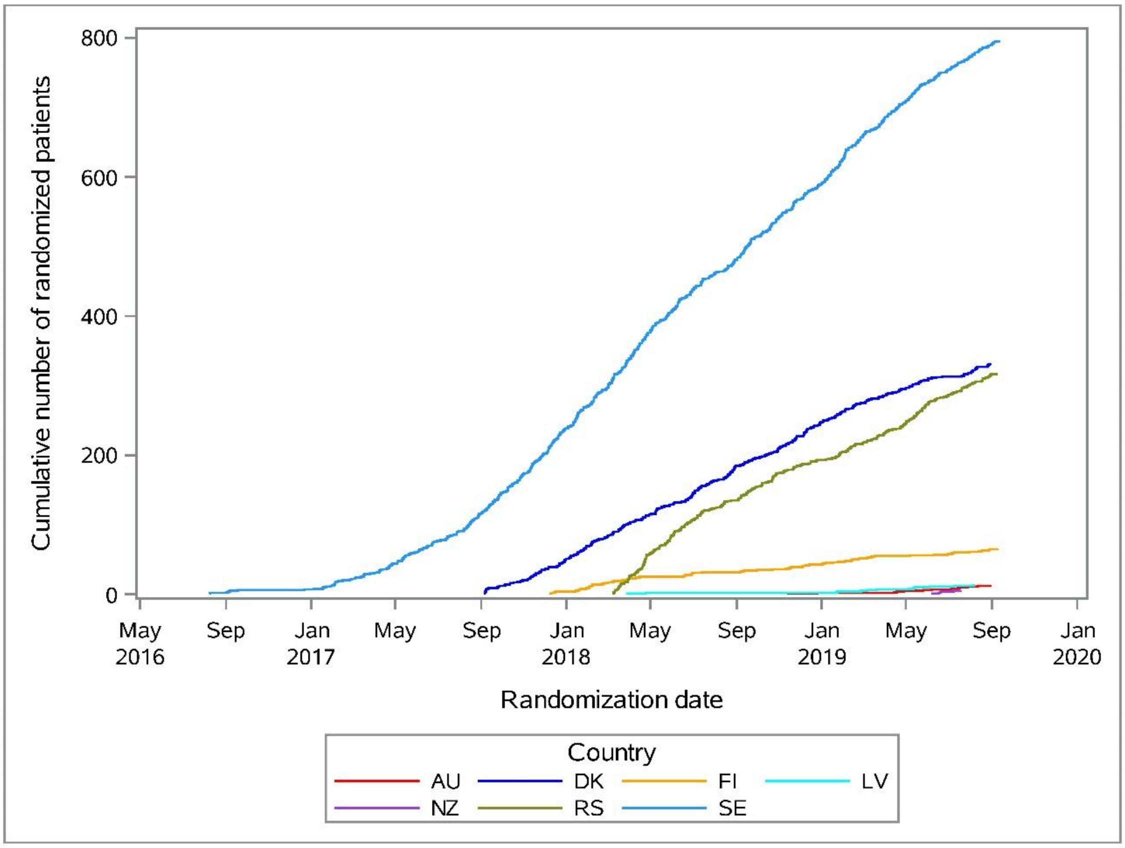

Figure 2. Consort flow chart.

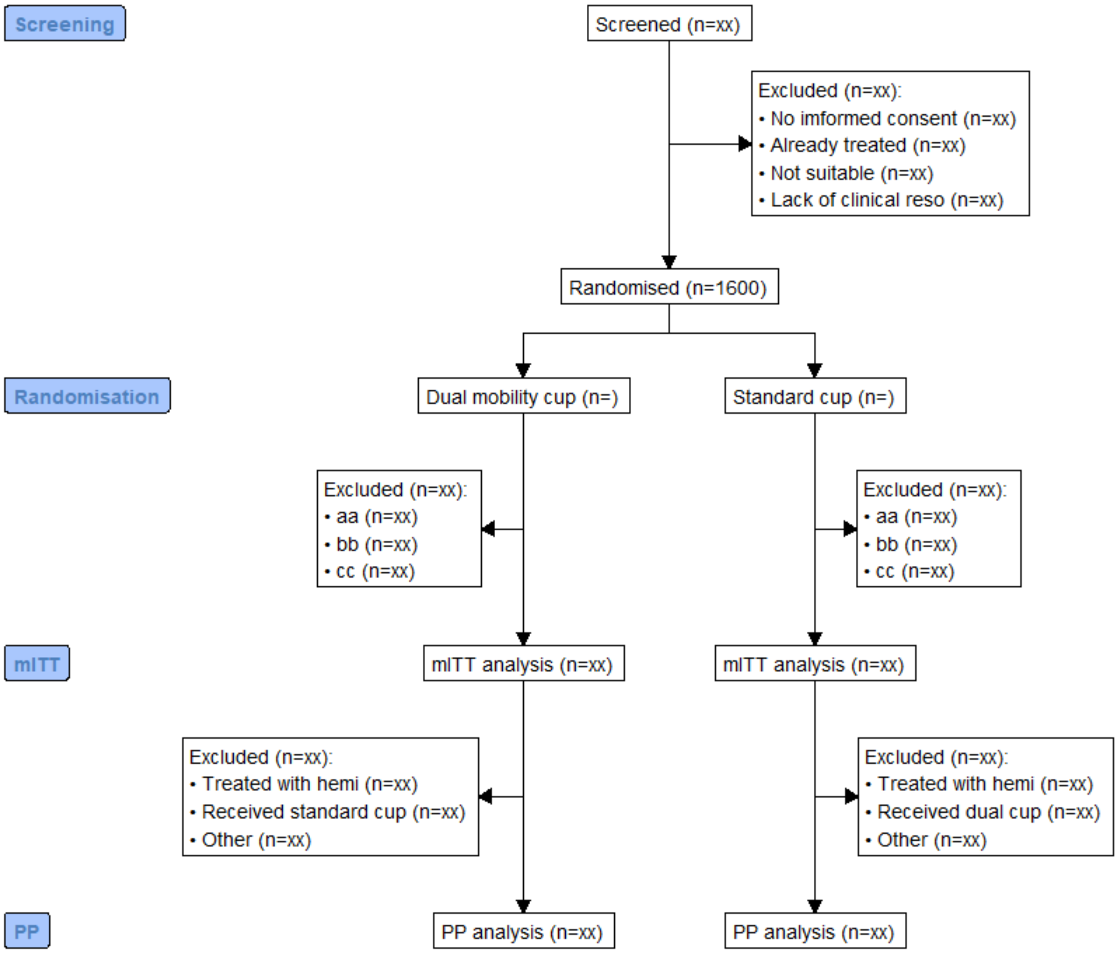

Table 5.1. Background characteristics, mITT

Variable	Statistic/level	DMC N	Control N	Total N
Age (years)	n			
	Median (Q1 – Q3)			
	Mean (SD)			
	Min - Max			
Sex, n (%)	Female			
	Male			
BMI	n			
	Median (Q1 – Q3)			
	Mean (SD)			
	Min - Max			
BMI categorised, n (%)	Underweight: < 18.5			
	Normal weight: 18.5–24.9			
	Overweight: 25.0–29.9			
	Obesity: 30.0–34.9			
	Severe obesity: ≥ 35.0			
ASA class, n (%)	I			
	II			
	III			
	IV			
	Missing			
Surgical approach, n (%)	Direct lateral			
	Posterior			
	Missing			
Femoral neck length, n (%)	Standard			
	Extended			
	Extra extended			
	Missing			
Cup diameter	n			
	Median (Q1 – Q3)			
	Min - Max			
Femoral head diameter	n			
	Median (Q1 – Q3)			

Variable	Statistic/level	DMC<br>N	Control<br>N	Total<br>N
	Min - Max			
Manufacturer[1] of cup, n (%)	Smith & Nephew			
	Fischer Medical			
	Ortotech			
	Zimmer Biomet			
	Stryker			
	Link Sweden			
	DePuy Johnson & Johnson			
	Other			
	Missing			
Type of stem, n (%)	Exeter			
	MS-30			
	SPII			
	CPT			
	C-Stem			
	Corail			
	Taperfit			
	Other			
	Missing			
Type of cement – femur, n (%)	Cementless			
	PALACOS			
	COPAL			
	Refobacin			
	Optipac			
	Simplex			
	Other			
	Missing			
Type of cement – cup, n (%)	Cementless			
	PALACOS			
	COPAL			
	Refobacin			
	Optipac			

[1] More levels might appear in final data, both in manufacturer of cup and manufacturer of stem variables.

Variable	Statistic/level	DMC N	Control N	Total N
	Other			
	Missing			
Manufacturer of stem, n (%)	Zimmer Biomet			
	Stryker			
	Link Sweden			
	DePuy Johnson & Johnson			
	Other			
	Missing			
Combined offset	Standard offset			
	High offset			
	Missing/Other			

Table 5.2. Background characteristics, mITT, by sex subgroups [same outline as Table 5.1]

Table 5.3. Background characteristics, mITT, by surgical approach subgroups [same outline as Table 5.1]

Table 5.4. Background characteristics, mITT, by country subgroups [same outline as Table 5.1]

Table 5.5. Background characteristics, PP [same outline as Table 5.1]

Table 5.5. PROMs at baseline, mITT

Variable	Statistic/level	DMC N	Control N	Total N
EQ - 5D Mobility at baseline, n (%)	No problem			
	Slight problems			
	Moderate problems			
	Severe problems			
	Extreme problems			
	Missing			

Variable	Statistic/level	DMC<br>N	Control<br>N	Total<br>N
EQ - 5D<br>Self-care at baseline, n (%)	No problem			
	Slight problems			
	Moderate problems			
	Severe problems			
	Extreme problems			
	Missing			
EQ - 5D<br>Usual activities at baseline, n (%)	No problem			
	Slight problems			
	Moderate problems			
	Severe problems			
	Extreme problems			
	Missing			
EQ - 5D<br>Pain/Discomfort at baseline	No problem			
	Slight problems			
	Moderate problems			
	Severe problems			
	Extreme problems			
	Missing			
EQ - 5D<br>Anxiety/Depression at baseline, n (%)	No problem			
	Slight problems			
	Moderate problems			
	Severe problems			
	Extreme problems			
	Missing			
EQ – 5D index	n			
	Median (Q1 – Q3)			
	Mean (SD)			
	Min - Max			
VAS	n			
	Median (Q1 – Q3)			
	Mean (SD)			
	Min - Max			

Table 6.1. Event rates, mITT

Outcomes	DMC<br>N = xxx			Control<br>N = xxx			Total<br>N = xxxx		
	Events (%)	Person time	Rate	Events (%)	Person time	Rate	Events (%)	Person time	Rate
Dislocation within 1 year									
PJI within 1 year									
Reoperation within 1 year									
Mortality within 90 days									
Mortality within 1 year									
Composite of dislocation or mortality within 1 year									

Table 6.2. Event rates, PP [same outline as Table 6.1]

Figure 3.1.1 Kaplan-Meier estimate of participants with dislocation within 1 year, mITT

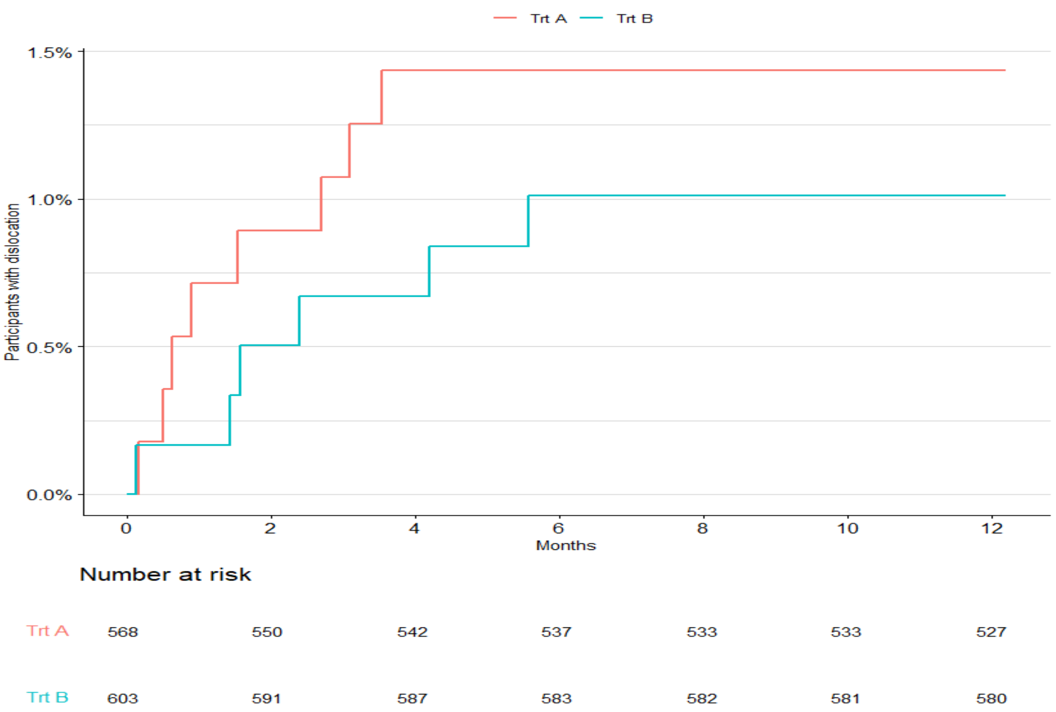

Figure 3.1.2 Kaplan-Meier estimate of participants with dislocation within 1 year, PP

Figure 3.2.1 Kaplan-Meier estimate of participants with any reoperations within 1 year, mITT

Figure 3.2.1 Kaplan-Meier estimate of participants with any reoperations within 1 year, PP

Figure 3.3.1 Kaplan-Meier estimate of participants with PJI within 1 year, mITT

Figure 3.3.2 Kaplan-Meier estimate of participants with PJI within 1 year, PP

Figure 3.4.1 Kaplan-Meier estimate of participants with mortality within 90 days, mITT

Figure 3.4.2 Kaplan-Meier estimate of participants with mortality within 90 days, PP

Figure 3.5.1 Kaplan-Meier estimate of participants with mortality within 1 year, mITT

Figure 3.5.2 Kaplan-Meier estimate of participants with mortality within 1 year, PP

Figure 3.6.1 Kaplan-Meier estimate of participants with composite of dislocation/mortality within 1 year, mITT

Figure 3.6.2 Kaplan-Meier estimate of participants with composite of dislocation/mortality within 1 year, PP

Table 7.1. Primary analyses for all time-to-event outcomes, mITT

Outcome		Model*	HR (95% CI)	P value for treatment effect
Primary outcome	Dislocation within 1 year	Adjusted		
		Unadjusted		
Secondary outcomes	PJI within 1 year	Adjusted		
		Unadjusted		
	Reoperation within 1 year	Adjusted		
		Unadjusted		
	Mortality within 90 days	Adjusted		
		Unadjusted		
	Mortality within 1 year	Adjusted		
		Unadjusted		
	Composite of dislocation or mortality within 1 year	Adjusted		
		Unadjusted		

*Adjusted models include age, sex, surgical approach and country in addition to randomised treatment, primary results.

Table 7.2. Primary analyses for all time-to-event outcomes, PP

Table 8. Subgroup analyses for the primary outcome at 1 year, mITT

Subgroup	Level	DMC Events/N (%)	Control Events/N (%)	HR (95% CI)	P-value for interaction
Country	Sweden				
	UK				
Sex	Female				
	Male				
BMI	Underweight				
	Normal weight				
	Overweight				
	Obesity				
	Severe obesity				
ASA class	I				
	II				
	III				
	IV				
Femoral neck length	Standard				
	Extended				
	Extra extended				
Cup diameter	continuous	NA	NA	NA	
Femoral head diameter	continuous	NA	NA	NA	
Acetabular component fixation	Cementless				
	Cemented				
Femoral component fixation	Cementless				
	Cemented				
Type of cement - cup	Optipac				
	Palacos				
	Copal				
Type of cement - femur	Optipac				
	Palacos				
	Copal				
Surgical approach	Direct lateral				
	Posterior				

*Figure 4 Forest plot (example plot, no subgroups will be presented for age, BMI subgroups with 5 levels as stated in SAP, p-value for interaction term trt * bmi as continuous variable) with HR (95% CI) per subgroup level for dislocation within 1 year, mITT*

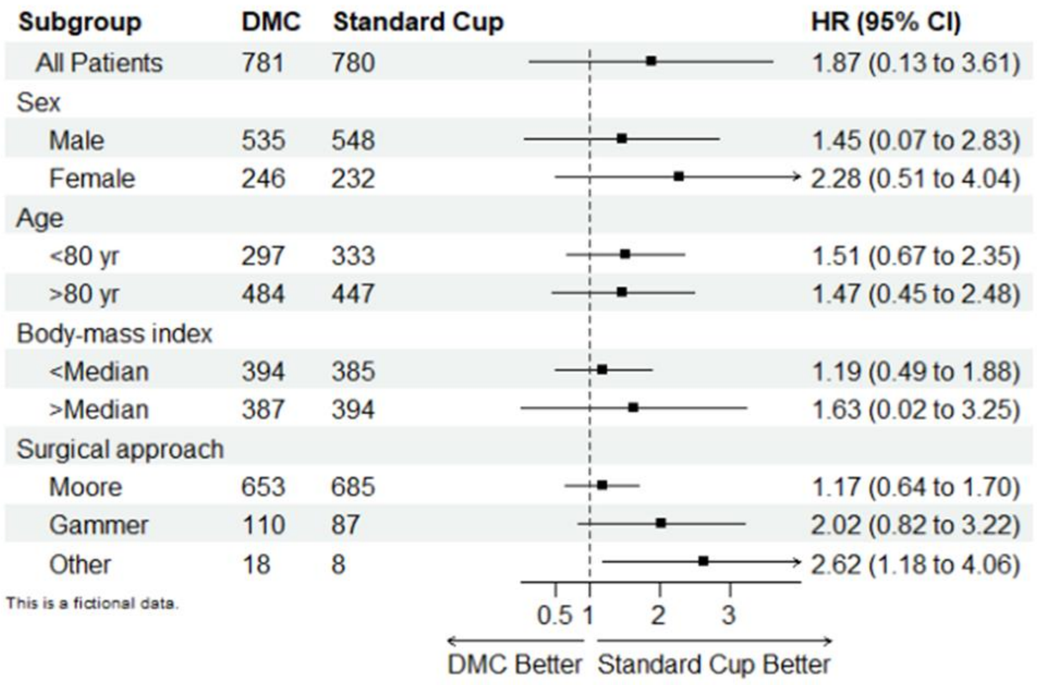

Figure 5. Interaction of treatment and age at randomisation for the primary outcome, mITT. Estimated hazard ratio with 95% pointwise confidence bands (Plotted as restricted cubic splines)

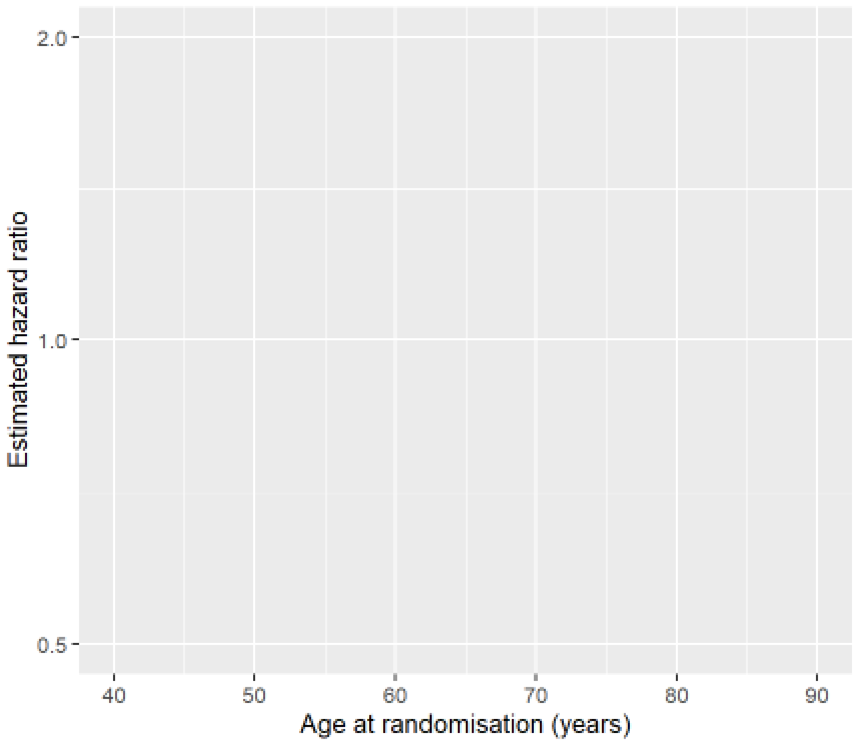

Figure 6. Interaction of treatment and BMI at randomisation for the primary outcome, mITT. Estimated hazard ratio with 95% pointwise confidence bands (Plotted as restricted cubic splines)

Table 9. Supplementary analyses for all time-to-event outcomes using logistic regression models, mITT [The same will be done for PP]

Outcome		Model*	OR (95% CI)	P value for treatment effect
Primary outcome	Dislocation within 1 year	Adjusted		
		Unadjusted		
Secondary outcomes	PJI within 1 year	Adjusted		
		Unadjusted		
	Reoperation within 1 year	Adjusted		
		Unadjusted		
	Mortality within 90 days	Adjusted		
		Unadjusted		
	Mortality within 1 year	Adjusted		
		Unadjusted		
	Composite of dislocation or mortality within 1 year	Adjusted		
		Unadjusted		

*Adjusted models include age, sex, surgical approach and country in addition to randomised treatment

Table 10. Supplementary analyses for all time-to-event outcomes, risk differences based on KM estimates and standard errors, mITT [The same will be done for PP]

Outcome	Risk Difference* (95% CI)	P value for treatment effect
Dislocation within 1 year		
PJI within 1 year		
Reoperation within 1 year		
Mortality within 90 days		
Mortality within 1 year		
Composite of dislocation or mortality within 1 years		

*Based on KM estimates and standard errors

Figure 6.1 EQ-5D: Mobility at 1 year, by treatment, mITT observed cases.

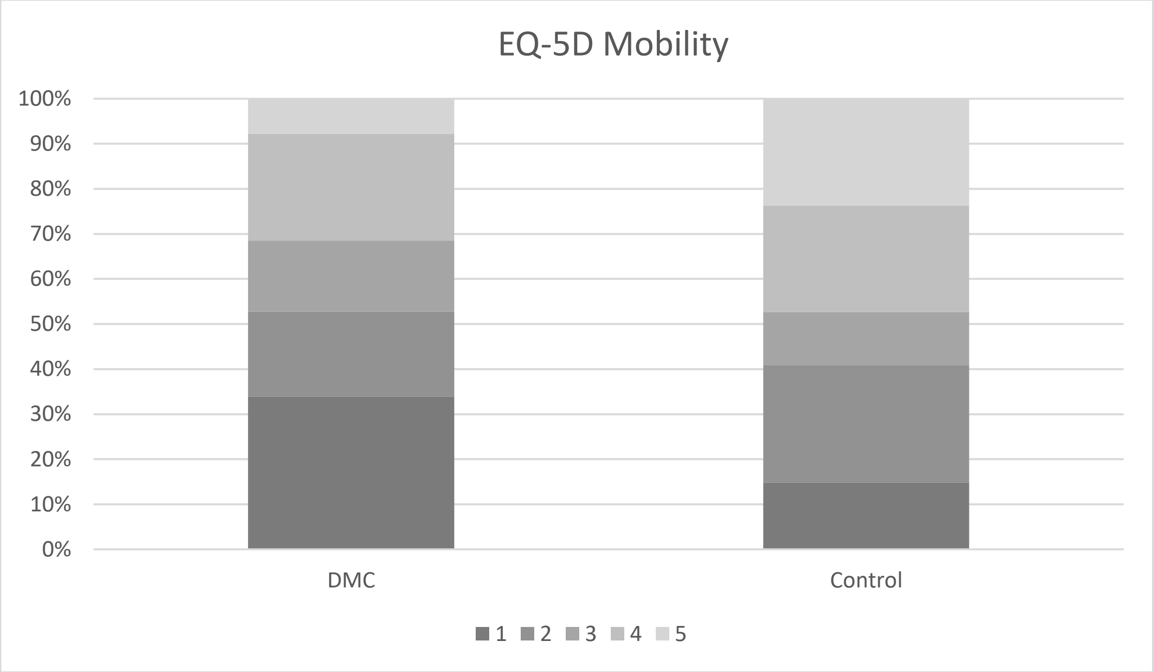

Figure 6.2 EQ-5D: Activities at 1 year, by treatment, mITT observed cases.

Figure 6.3 EQ-5D: Self-care at 1 year, by treatment, mITT observed cases.

Figure 6.4 EQ-5D: Pain at 1 year, by treatment, mITT observed cases.

Figure 6.5 EQ-5D: Anxiety at 1 year, by treatment, mITT observed cases.

Table 11. EQ-5D domain scores at 1 year

Domain	Level	mITT		PP		Observed cases	
		DMC<br>N	Control<br>N	DMC<br>N	Control<br>N	DMC<br>N	Control<br>N
Mobility	1						
	2						
	3						
	4						
	5						
	Deceased						
	Missing						
Usual Activities	1						
	2						
	3						
	4						
	5						
	Deceased						
	Missing						
Self-care	1						
	2						
	3						
	4						
	5						
	Deceased						
	Missing						
Pain & Discomfort	1						
	2						
	3						
	4						
	5						
	Deceased						
	Missing						
Anxiety & Depression	1						
	2						
	3						
	4						
	5						
	Deceased						
	Missing						

Table 12. Statistical analysis of EQ-5D domain scores at 1 year, mITT.

Domain	Model	OR[2] (95% CI)	P-value
Mobility	Model 1[3]		
	Model 2[4]		
Usual Activities	Model 1		
	Model 2		
Self-care	Model 1		
	Model 2		
Pain & Discomfort	Model 1		
	Model 2		
Anxiety & Depression	Model 1		
	Model 2		

Table 17. VAS at 1 year, mITT.

Variable	Statistic	DMC<br>N	Control<br>N	Total<br>N
VAS	n			
	Median (Q1 – Q3)			
	Mean (SD)			
	Min - Max			
EQ-5D Index	n			
	Median (Q1 – Q3)			
	Mean (SD)			
	Min - Max			

[2] OR for higher domain scores
[3] Model 1 includes randomised treatment and baseline score. Missing baseline score imputed using the median, and missing outcome scores due to death set to 6. No other imputation of missing outcome scores.
[4] Model 2 includes randomised treatment and baseline score. Missing outcome scores due to death set to 6, observed cases only for baseline scores and other missing outcome scores.

Table 18.1. Statistical analysis of VAS at 1 year.

Outcome	mITT		PP	
	OR* (95% CI)	P-value	OR (95% CI)	P-value
VAS				

**OR from proportional odds logistic regression model.*

Table 18.2. Statistical analysis of EQ-5D index at 1 year.

Outcome	mITT		PP	
	Est* (95% CI)	P-value	Est (95% CI)	P-value
EQ-5D Index				

** Treatment effect from linear regression model*

Figure 7 ECDF plot of VAS change from baseline, mITT.

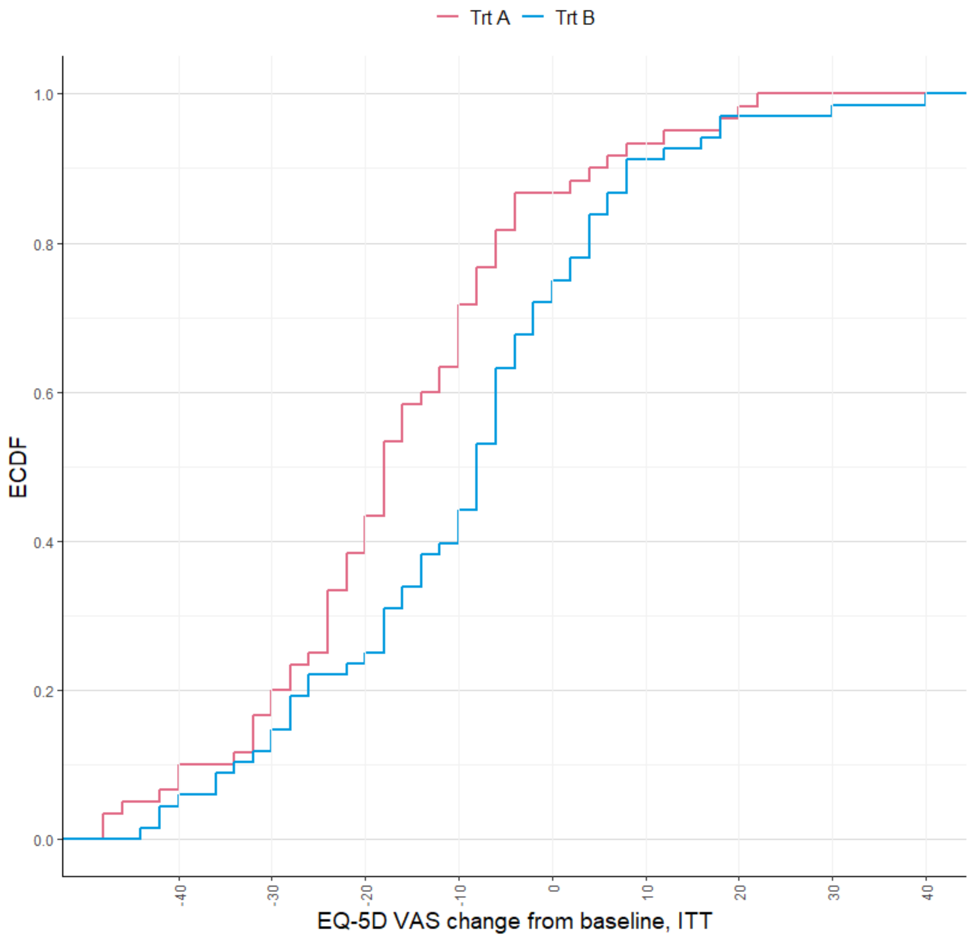

Figure 5.2 ECDF plot of VAS change from baseline, PP.

Listing 1. Withdrawals.

Listing 2. Patients excluded from the mITT.

Listing 3. Protocol deviations.

Appendix 2 − Derived Variables

Statistical Analysis Plan: Derived variables

Study Code: DUALITY

Study Title: The DUALITY trial—a register-based, randomised controlled trial to investigate dual mobility cups in hip fracture patients

Based on protocol version and date: 3.3, 26-FEB-2025

Biostatistician: Tatevik Ghukasyan Lakic

Reviewed and approved by Biostatistician (UCR);

Name: Ollie Östlund Signature: Date:

Approved by Sponsor's Representative;

Name: Nils Hailer Signature: Date:

SAP version: final 1.0

1. Introduction

This document is an attachment to the Statistical Analysis Plan (DUALITY final SAP v 1.0.docx) and aims to provide a detailed description of variable derivations. It includes all variables used in the tables and figures of the DUALITY final SAP (OS) v 1.0.docx document and includes both the Swedish and UK cohorts.

Variable label	Variable name in adb	Variable levels	Variable in Swedish data	Variable in UK data
Age	age	numeric	I_SCREE_INJ_AGE, Inj_Age	bho_dob
Sex	sex	Female, Male	I_SCREE_INJ_SEX, SEX	ps_sex
BMI	bmi	numeric	HP_BMI	w12_ab_height<br>w12_ab_weight
ASA class	asa	numeric	HP_ASA	bho_gradeasa

Variable label	Variable name in adb	Variable in Swedish data	Variable in UK data
Cup diameter	cup_diam	HP_AcetCup_CupSize	w12_ab_cup_outer
Femoral head diameter	femhead_diam	HP_FemCaput	w12_ab_head_diam
Combined offset	comb_offset	HP_fem_stem_offset, HP_CaputLength	w12_ab_stem_offset<br>w12_ab_head_len

Surgical approach (variable name in adb: approach)		
**Variable value in adb**	**Based on (variable name in raw data)**	**Description (variable value in raw data)**
Direct lateral	HP_Incision	Direkt lateralt snitt i rygglâge (Hardinge)
		Direkt lateralt snitt med trochanterosteotomi
		Direkt lateralt snitt i sidolâge (Gammer)
		Watson-Jones (original)
		Direkt anteriort snitt (DAA)
	w12_ab_approach	Lateral (Hardinge)
	w12_ab_approach_oth	Anterolateral
		Modified Hardinge -capsulotomy
		Modified Hardinge T Capsulotomy
		modified hardinge, T Capsulotomy
Posterior	HP_Incision	Bakre snitt i sidolâge (Moore)
	w12_ab_approach	Posterior
	w12_ab_approach_oth	posterio-lateral
		postero-lateral

Femoral neck length (variable name in adb: femstemoffset)		
**Variable value in adb**	**Based on (variable name in raw data)**	**Description (variable value in raw data)**
Standard	HP_fem_stem_offset	33.5 mm offset
		37.5 mm offset
		Standard för aktuell modell
		Lateraliserad
	w12_ab_stem_offset	<44
Extended	HP_fem_stem_offset	44 mm offset
		Extra offset
		Varus
	w12_ab_stem_offset	44-49
Extra extended	HP_fem_stem_offset	50 mm offset
	w12_ab_stem_offset	50

Manufacturer of cup (variable name in adb: cupmanu)	
**Based on (variable name in raw data)**	**Description (variable value in raw data)**
HP_acetcup_supplier	Smith & Nephew
	Zimmer Biomet
	Stryker
	Link Sweden
	DePuy Johnson & Johnson
	Ortotech
	Fischer Medical

More levels will be available in final data, all levels will be listed

Manufacturer of stem (variable name in adb: stemmanu)	
**Based on (variable name in raw data)**	**Description (variable value in raw data)**
HP_Fem_stem_supplier	Link Sweden
	Stryker
	Zimmer Biomet
	DePuy Johnson & Johnson

More levels will be available in final data, all levels will be listed

**Type of stem (variable name in adb: stemtype)**		
**Variable value in adb**	**Based on (variable name in raw data)**	**Description (variable value in raw data)**
Exeter	HP_fem_stem_name	Exeter standard
	w12_ab_stem	Exeter V40
		Exeter
	w12_ab_stem_oth	Stryker
		Stryker v40
MS-30	HP_fem_stem_name	MS-30 polerad
SPII	HP_fem_stem_name	SPII Revision (>150mm)
		SPII standard
CPT	w12_ab_stem	CPT CoCr Stem
	w12_ab_stem_oth	CPT Hip System
		Zimmer CPT
		Zimmer CPT 12/14
		Zimmer CPT 12/14 hip stem
		Zimmer CPT 12/14 stem
C-Stem	w12_ab_stem	C-Stem AMT Cemented Stem
		C-Stem Cemented Stem
Corail	HP_fem_stem_name	Corail coxa vara
		Corail standard
	w12_ab_stem	Corail
Taperfit	w12_ab_stem	Taperfit Cemented Stem
	w12_ab_stem_oth	CORIN Taperfit
		Corin Taperfit
Other	w12_ab_stem	Accolade II
		Accolade
		Polarstem Cementless
		Avenir Muller Cementless
		AMIStem-C
		Quadra-S
	w12_ab_stem_oth	Depuy cemented stem standard
	HP_fem_stem_name	Wagner Cone

**Type of cement – femur (variable name in adb: femstemcem)**		
**Variable value in adb**	**Based on (variable name in raw data)**	**Description (variable value in raw data)**
Cementless	HP_FemStemCemType	Cementfritt
	w12_ab_stemcem_yn	0 (No)
PALACOS	HP_ FemStemCemType	66017569 PALACOS R+G 2x40 NE
		66017772 PALACOS R+G 1x60 INT
		66044273 PALACOS R+G PRO 55
		66044274 PALACOS R+G PRO 75
	w12_ab_stemcem_heraeus	Palacos Antibiotic
		Palacos Fast Setting
		Palacos
COPAL	HP_ FemStemCemType	66017790 COPAL G+C 1x40 INT
		66038973 COPAL G+V 1x40 INT
	w12_ab_stemcem_heraeus	Copal G+C
		Copal G+V
Refobacin	HP_FemStemCemType	3003920002-3 RefobacinÂ® Bone Cement R 2 x 20
		3003940001-3 RefobacinÂ® Bone Cement R 1 x 40
		3003940002-3 RefobacinÂ® Bone Cement R 2 x 40
		3011630001-3 RefobacinÂ® Revision 1 x 40
	w12_ab_stemcem_zimmer	Biomet Refobacin
Optipac	HP_FemStemCemType	4710500394-3 Optipac 40 RefobacinÂ® Bone Cement R
		4711500396-3 Optipac 60 RefobacinÂ® Bone Cement R
		4712500398-3 Optipac 80 RefobacinÂ® Bone Cement R
		4730501163-3 Optipac 40 Refobacin® Revision
		4711500396-1 Optipac 60 Refobacin® Bone Cement R
		4712500398-1 Optipac 80 Refobacin® Bone Cement R
	w12_ab_stemcem_zimmer	Biomet Optipac Refobacin
		Biomet Optipac Refobacin Plus
Simplex	w12_ab_stemcem_manu	Stryker
Other	w12_ab_stemcem_manu	DePuy
	w12_ab_stemcem_depuy	CMW1 Plain Cement
	w12_ab_stemcem_heraeus	Palamed Antibiotic

Type of cement – cup (variable name in adb: acetcupcem)		
**Variable value in adb**	**Based on (variable name in raw data)**	**Description (variable value in raw data)**
Cementless	HP_ AcetCupCemType	Cementfritt
	w12_ab_cupcem_yn	0 (No)
PALACOS	HP_ AcetCupCemType	66017569 PALACOS R+G 2x40 NE
		66044273 PALACOS R+G PRO 55
		66044274 PALACOS R+G PRO 75
	w12_ab_cupcem_heraeus	Palacos Antibiotic
		Palacos Fast Setting
		Palacos
COPAL	HP_ AcetCupCemType	66017790 COPAL G+C 1x40 INT
		66038973 COPAL G+V 1x40 INT
	w12_ab_cupcem_heraeus	Copal G+C
Refobacin	HP_ AcetCupCemType	3003920002-3 RefobacinÂ® Bone Cement R 2 x 20
		3003940001-3 RefobacinÂ® Bone Cement R 1 x 40
		3003940002-3 RefobacinÂ® Bone Cement R 2 x 40
		3011630001-3 RefobacinÂ® Revision 1 x 40
Optipac	HP_ AcetCupCemType	4710500394-3 Optipac 40 RefobacinÂ® Bone Cement R
		4711500396-3 Optipac 60 RefobacinÂ® Bone Cement R
		4712500398-3 Optipac 80 RefobacinÂ® Bone Cement R
		4710500394-1 Optipac 40 RefobacinÂ® Bone Cement R
Other	w12_ab_cupcem_manu	DePuy
		Stryker

2. Time-to-event outcomes

2.1 Dislocation:

Swedish cohort: Dislocations will primarily be obtained from NPR, supplemented by SLR and the clinical event adjudication (CEA) file provided by monitors. Only events occurring after the primary hip surgery date will be counted.
From SLR the following variables will be used: HR_KVA1 HR_KVA1OtherOpenSurg HR_KVA2 HR_KVA3 HR_KVA4 HR_ICD1 HR_ICD2 HR_ICD3 for identifying events, and the variable HR_SurgDate will be used as the event date.
From NPR all diagnosis code variables (DIA1-DIA30) and all procedure code variables (op1-op30) will be used, regardless of position. If a match is found in any diagnosis code only, the hospital admission date (INDATUM) will be used as the event date. If a match is found in a procedure code, the corresponding procedure date (OPD) will be used instead.
In addition, events identified through clinical event adjudication will be included, as provided in a separate file by the study monitors. In cases where multiple data sources report differing event dates, the earliest available date will be selected.

The codes below will be used for dislocation outcomes:

Outcome	Diagnosis codes	Procedure codes
Dislocation	M24.3, M24.4, M24.4F, S73.0, T93.3	NFH00, NFH02, NFH20, FH21, NFH22

UK cohort: Data on event outcomes will be obtained from “Complication Form”. A complication is treated as dislocation if:

a) the variable com_complication (Following treatment, which of the following complications occurred?) gets value ***13*** – *Dislocation*, **or**
b) the variable com_opreason (What was the principal reason that further surgery was needed?) gets value **1 -** *Reduction of dislocation*.

The corresponding com_eventdate (Date of Event) will be used as the event date.

2.2 Periprosthetic joint infection (PJI)

Swedish cohort: PJI will be obtained from NPR, supplemented by SLR. Only events occurring after the primary hip surgery date will be counted.
From SLR the following variables will be used: HR_KVA1 HR_KVA1OtherOpenSurg HR_KVA2 HR_KVA3 HR_KVA4 HR_ICD1 HR_ICD2 HR_ICD3 for identifying events, and the variable HR_SurgDate will be used as the event date.
From NPR all diagnosis code variables (DIA1-DIA30) and all procedure code variables (op1-op30) will be used, regardless of position. If a match is found in any diagnosis code only, the hospital admission date (INDATUM) will be used as the event date. If a match is found in a procedure code, the corresponding procedure date (OPD) will be used instead.
In cases where multiple data sources report differing event dates, the earliest available date will be selected.

The codes below will be used for PJI outcomes:

Outcome	Diagnosis code	Procedure code
PJI	M00.0, M00.0F, M00.1, M00.2, M00.2F, M00.8, M00.8F, M00.9, M00.9F, M86.0F, M86.1F, M86.6, M86.6F, T81.4, T84.5, T84.5F, T84.5X, T84.7, T84.7F, T846F[1]	NFSx, NFA12, TNF05, TNF10

UK cohort: Data on PJI outcome will be obtained from "Complication Form". A complication is treated as PJI if:

a) the variable com_complication (Following treatment, which of the following complications occurred?) gets value ***14*** **-** *Wound infection*, or
b) The variable com_opreason (What was the principal reason that further surgery was needed?) gets value **2** - *Washout or debridement*.

The corresponding com_eventdate (Date of Event) will be used as the event date.

[1] Added while preparing the SAP

2.3 Any reoperation

Swedish cohort: Any reoperation will primarily be obtained from NPR, supplemented by SLR and the clinical event adjudication (CEA) file provided by monitors. Only events occurring after the primary hip surgery date will be counted.
From SLR the following variables will be used: HR_KVA1 HR_KVA1OtherOpenSurg HR_KVA2 HR_KVA3 HR_KVA4 HR_ICD1 HR_ICD2 HR_ICD3 for identifying events, and the variable HR_SurgDate will be used as the event date.
From NPR all procedure code variables (op1-op30) will be used, regardless of position, and the date of corresponding procedure (OPD) will be considered the event date.
In cases where multiple data sources report differing event dates, the earliest available date will be selected.

The codes below will be used for any reoperation outcomes:

**Outcome**	**Procedure code**
**Any reoperation**	NFH02, FH21, NFH22 (dislocations with open reduction[2])<br><br>NFSx, NFA12, TNF05, TNF10 (PJI)<br><br>NFA00-22, NFA31-32, NFCx, NFF01–12, NFL09–19, NFL39–49, NFL69–99, NFM09–29, NFM49, NFM79–99, NFTx, NFWx

UK cohort: Data on this outcome will be obtained from "Complication Form". A complication is treated as Any reoperation if the variable com_complication (Following treatment, which of the following complications occurred?)

a) gets any of the values ***13, 14 or 12*** - *Additional surgery related to the hip fracture (including intra-operative)*, and at the same time
b) the variable com_surgery (Did the participant have additional surgery related to this complication?) gets the value ***1*** - *Yes* and
c) the variable com_surgerydate (Please state date of the surgery) is non-missing.

The corresponding com_surgerydate will be used as the event date.

[2] Note: the codes NFH00 and NFH20 are removed while preparing the SAP

2.4 Death

Swedish cohort – variable “DateOfDeath”.

UK cohort – variable “dth_eventdate”.

3. PROMs

For the Swedish cohort, the PROMs at baseline will be obtained from SFR, and PROMs at 1 year will primarily be obtained from SLR. In case of missing values in SLR but available in SFR, the values for SFR will be used.

For the EQ-5D utility index the NICE recommended UK algorithms will be used both for Swedish and UK cohorts. For sensitivity analyses, also Swedish valuation will be used.

Signature page

This document has been electronically signed using eduSign.

eduSign

Validate signed documents at: https://validator.edusign.sunet.se

SUNET
